# Structure Activity Relationship of Brevenal Hydrazide Derivatives

**DOI:** 10.3390/md12041839

**Published:** 2014-03-28

**Authors:** Allan Goodman, Jennifer R. McCall, Henry M. Jacocks, Alysha Thompson, Daniel Baden, William M. Abraham, Andrea Bourdelais

**Affiliations:** 1 Center for Marine Science, University of North Carolina Wilmington (UNCW), 5600 Marvin K. Moss Lane, Wilmington, NC 28409, USA; E-Mails: goodmana@uncw.edu (A.G.); mccalljr@uncw.edu (J.R.M.); hjacocks@ec.rr.com (H.M.J.); amthomp2@ncsu.edu (A.T.); baden@uncw.edu (D.B.); 2 Department of Research, Mount Sinai Medical Center, 4300 Alton Road, Miami Beach, FL 33140, USA; E-Mail: william.abraham@msmc.com

**Keywords:** brevenal, brevetoxin, derivatives, structure activity relationship, cystic fibrosis, drug development

## Abstract

Brevenal is a ladder frame polyether produced by the dinoflagellate *Karenia brevis*. This organism is also responsible for the production of the neurotoxic compounds known as brevetoxins. Ingestion or inhalation of the brevetoxins leads to adverse effects such as gastrointestinal maladies and bronchoconstriction. Brevenal shows antagonistic behavior to the brevetoxins and shows beneficial attributes when administered alone. For example, in an asthmatic sheep model, brevenal has been shown to increase tracheal mucosal velocity, an attribute which has led to its development as a potential treatment for Cystic Fibrosis. The mechanism of action of brevenal is poorly understood and the exact binding site has not been elucidated. In an attempt to further understand the mechanism of action of brevenal and potentially develop a second generation drug candidate, a series of brevenal derivatives were prepared through modification of the aldehyde moiety. These derivatives include aliphatic, aromatic and heteroaromatic hydrazide derivatives. The brevenal derivatives were tested using *in vitro* synaptosome binding assays to determine the ability of the compounds to displace brevetoxin and brevenal from their native receptors. A sheep inhalation model was used to determine if instillation of the brevenal derivatives resulted in bronchoconstriction. Only small modifications were tolerated, with larger moieties leading to loss of affinity for the brevenal receptor and bronchoconstriction in the sheep model.

## 1. Introduction

**Figure 1:**
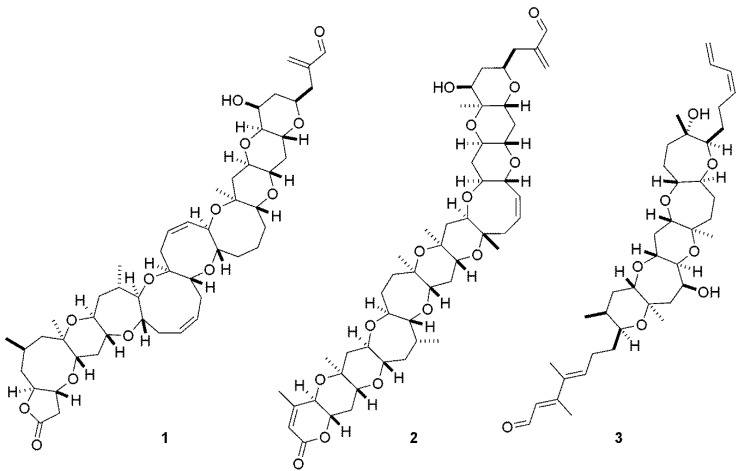
Chemical structures of brevetoxins PbTx-1 (**1**) and PbTx-2 (**2**) and the antagonist, brevenal (**3**).

The marine dinoflagellate *Karenia brevis* is associated with the phenomenon known as Florida Red Tide and produces a variety of ladder frame polyether compounds (LFPs), including the highly neurotoxic brevetoxins (**1**, **2**) (Figure 1) [1]. It has long been established that the brevetoxins elicit their effects through activation of site five of voltage gated sodium channels [2,3]. Activation of these sodium channels leads to neurological effects including respiratory difficulty, gastrointestinal maladies and sensation of temperature reversal.

In addition to these toxins, *K. brevis* produces brevenal (**3**) (Figure 1), a compound shown to possess antagonistic activity to the brevetoxins and other site five activators such as ciguatoxin [4,5,6,7]. In the sheep model of asthma, brevenal was able to both inhibit and reverse brevetoxin induced bronchoconstriction [8].

Furthermore, when administered alone, brevenal was found to increase tracheal mucosal velocity. This finding mirrored that of clinical drugs used in the treatment of cystic fibrosis and spurred the development of brevenal as a potential treatment for this disease. Recently, work completed in our laboratory has shown that brevenal binds to a site that is distinctly different, but linked to site five of voltage gated sodium channels (VGSCs) [9]. In this work, it was shown that brevenal was able to displace brevetoxin (**2**) from the site five on VSGCs, but brevetoxin was unable to displace brevenal from its binding site in reciprocal studies, leading to the conclusion that brevenal is an allostere for brevetoxin. 

In an attempt to further understand the mechanism of action of brevenal and potentially develop a second generation drug candidate, a series of brevenal derivatives were prepared through modification of the aldehyde moiety. These derivatives include aliphatic, aromatic and heteroaromatic derivatives. The brevenal derivatives were tested in *in vitro* synaptosome binding assays to determine the ability of the semi-synthetic compounds to displace brevetoxins and brevenal from their native receptors. 

**Scheme 1:**
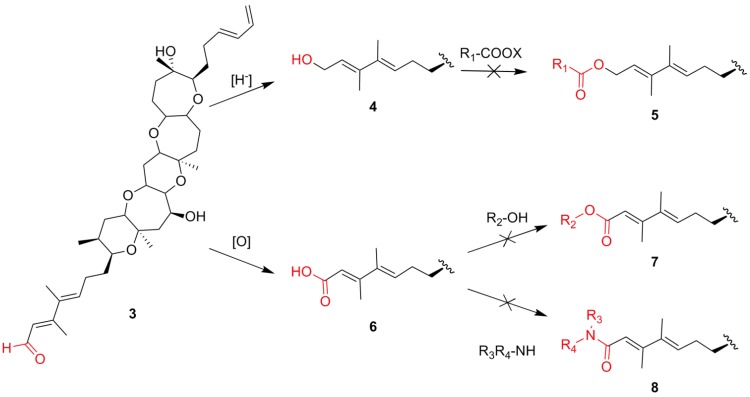
Synthetic strategy in an attempt to prepare ester and amide derivatives of brevenal.

Brevenal is more or less devoid of functional “handles” for SAR study, with the exception of the aldehyde moiety, which is the only functional group that can be readily modified and utilized from an SAR point of view. Various transformations can be performed on aldehyde functionalities including reduction, oxidation and reductive aminations to produce the alcohol, acid and amine derivatives, respectively (Scheme 1). All three of these aldehyde products can, potentially, be further derivatized to generate a wide range of compounds for SAR studies, and all three of these transformations were attempted. Reduction of the brevenal to give the alcohol (**4**) was achieved in high yield. However, attempts to prepare ester derivatives (**5**) were disappointing, as it was found that the alcohol (**4**) was unreactive towards any acids or acid derivatives including acid chlorides and anhydrides, even under extended reaction times and elevated temperatures. Oxidation of the aldehyde to the acid (**6**) required prolonged reaction times and was poor yielding. Even more frustrating, attempts to synthesize esters (**7**) or amides (**8**) were unsuccessful as the acid also proved unreactive and was not useful for SAR studies. Perhaps the most utilized modification of an aldehyde group is to perform reductive amination reactions to create amines. Previous attempts within our laboratory to perform this transformation with brevenal had proven unsuccessful. 

**Scheme 2:**
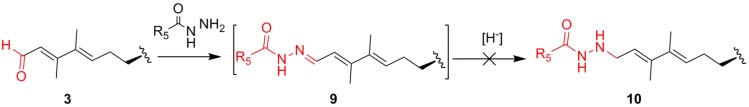
Attempted reductive amination type synthesis of hydrazide derivatives (**10**) led to stable Schiff base intermediates (**9**).

However, during reactions to prepare fluorescently labeled LFPs for development of a fluorescence based receptor binding assay [10], attachment of fluorescent hydrazides was found to be achievable in a rapid and high yielding manner (Scheme 2). Somewhat unexpectedly, the Schiff base intermediate (**9**) produced by the reaction of the aldehyde with the hydrazide was highly stable and was not susceptible to reduction with various borohydride reducing reagents such as sodium borohydride, cyanoborohydride, *etc*. to give the hydrazide derivatives (**10**). This stability likely arises from conjugation with the butadiene moiety. Indeed, cell uptake studies of fluorescent hydrazone brevenal conjugates indicate that the complexes stay intact intracellularly for at least 48 hours. Due to brevenal’s limited reactivity, a series of substituted hydrazide “Schiff base” derivatives (**9**) were prepared and studied for their ability to displace brevenal and brevetoxin from their respective receptors (Table 1, Table 2 and Table 3). 

**Table 1:** Aliphatic and small unsubstituted cyclic brevenal derivatives.

Compound	R	*K_i_*_(brevenal)_ (nM) ^a^	*K_i_*_(brevetoxin)_ (nM) ^a^		Compound	R	*K_i_*_(brevenal)_ (nM) ^a^	*K_i_*_(brevetoxin)_ (nM) ^a^
**3**	Brevenal	137 ± 16	1133 ± 153		**14**	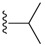	486 ± 85	300 ± 67
**11**	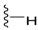	114 ± 61	DNB ^b^		**15**	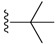	1195 ± 263	162 ± 22
**12**	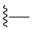	995 ± 292	496 ± 229		**16**	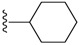	534 ± 31	DNB ^b^
**13**	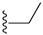	328 ± 59	163 ± 48					

^a^ Mean and SEM of at least 3 runs; ^b^ Does not bind.

**Table 2:** Aromatic and heteroaromatic derivatives of brevenal.

Compound	R	*K_i_*_(brevenal)_ (nM) ^a^	*K_i_*_(brevetoxin)_ (nM) ^a^		Compound	R	*K_i_*_(brevenal)_ (nM) ^a^	*K_i_*_(brevetoxin)_ (nM) ^a^
**3**	Brevenal	137 ± 16	1133 ± 153		**20**	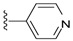	620 ± 239	75 ± 38
**17**	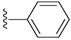	584 ± 104	DNB ^b^		**21**	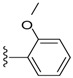	570 ± 33	DNB ^b^
**18**	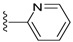	727 ± 149	DNB ^b^		**22**	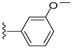	1037 ± 391	8 ± 3
**19**	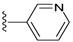	412 ± 148	>5000		**23**	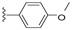	837 ± 163	DNB ^b^

^a^ Mean and SEM of at least 3 runs; ^b^ Does not bind.

**Table 3:** *Meta*-substituted phenyl derivatives of brevenal.

Compound	R	*K_i_*_(brevenal)_ (nM) ^a^	*K_i_*_(brevetoxin)_ (nM) ^a^		Compound	R	*K_i_*_(brevenal)_ (nM) ^a^	*K_i_*_(brevetoxin)_ (nM) ^a^
**3**	Brevenal	137 ± 16	1133 ± 153		**29**	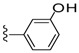	692 ± 135	>5000
**22**	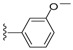	1037 ± 391	8 ± 3		**30**	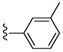	1018 ± 79	732 ± 287
**24**	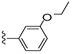	639 ± 131	DNB ^b^		**31**	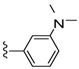	1745 ± 421	DNB ^b^
**25**	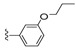	525 ± 133	DNB^b^		**32**	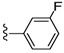	455 ± 155	440 ± 108
**26**	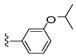	1081 ± 167	479 ± 140		**33**	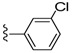	884 ± 445	109 ± 79
**27**	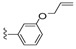	500 ± 142	253 ± 98		**34**	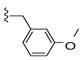	331 ± 97	23 ± 9
**28**	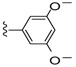	551 ± 224	>5000		**35**	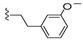	231 ± 37	66 ± 39

^a^ Mean and SEM of at least 3 runs; ^b^ Does not bind.

## 2. Results and Discussion

The first set of derivatives comprised of small aliphatic and unsubstituted cyclic substituents on the hydrazide (**11**–**16**) (Table 1). As can be seen in Table 1, addition of a formyl hydrazide moiety (**11**) is well tolerated and appears to be equipotent in affinity for the brevenal receptor compared to brevenal, while concomitantly being deleterious to brevetoxin displacement. Interestingly, introduction of a methyl substituent (**12**) led to an almost seven-fold decrease in affinity for the brevenal receptor. Increasing the size of the substituent (**12**–**16**) did not appear to regain any of the affinity for the brevenal receptor. However, small aliphatic substituents such as ethyl (**13**) or *t*-butyl groups (**15**) allow displacement of brevetoxin from its receptor in almost seven-fold lower concentrations compared to brevenal. 

The second set of derivatives looked at the inclusion of heterocyclic and electron rich aromatic substituents (**17**–**23**) (Table 2). From this data it can be seen that all modifications lead to decreased affinity for the brevenal receptor. However, when tested in the brevetoxin assay, the 3-methoxyphenyl analog (**22**) showed a more than 140-fold increase in potency for displacing the toxin compared to brevenal. This was somewhat confounding, given that the compound also suffered a seven-fold decrease in affinity for the brevenal receptor. 

Based on the results obtained for the series of compounds described in Table 2, a series of compounds with various substituents at the 3-position of the phenyl group was prepared (Table 3). Deviation from the methoxy substituted compound (**22**) with other ether compounds (**24**–**27**) led to compounds with unchanged or marginally better affinity for the brevenal receptor. Similarly, removal of the methyl group to give the phenol (**29**) also led to marginally higher affinity for the brevenal receptor. The effects on brevetoxin displacement were much more impacted by these modifications. None of the oxygen substituted compounds were able to displace brevetoxin at concentrations comparable to the lead compound **22**. Similarly, replacement of the methoxy substituent with a methyl (**30**), dimethylamino (**31**) or fluorine (**32**) group again led to little to no improvement in affinity for the brevenal receptor and was detrimental to brevetoxin displacement compared to **22**. Substitution of the methoxy group with a chloro moiety (**33**) led to a slight increase in affinity for the brevenal receptor, while retaining a modest ability to displace brevetoxin. Extension of the linker between the hydrazide carbonyl and the 3-methoxyphenyl substituent from 0 carbons (**22**) to 1 carbon (**34**) led to a significant increase in affinity for the brevenal receptor, while simultaneously reducing the potency for brevetoxin displacement. Interestingly, further extension of the linker from 1 carbon (**34**) to 2 carbons (**35**) continued this trend with improved binding to the brevenal receptor with concomitant loss of ability to displace brevetoxin.

From the SAR generated for the brevenal receptor, it appears that most modifications of the aldehyde portion of brevenal are not well tolerated. Indeed, any substitution larger than a formyl hydrazide substituent leads to decreased affinity for the brevenal receptor compared to brevenal. This indicates that the receptor for brevenal is highly specialized to this ladder frame polyether. Interestingly, inclusion of “remote” substituents such as the 3-methoxyphenylethyl hydrazide (**35**) led to a compound with minor loss in affinity for the brevenal receptor. Possible explanations for this retention in affinity for the brevenal receptor could be that either the methoxyphenyl group is extended beyond areas of the brevenal receptor that lead to negative interactions seen with the shorter hydrazides or that any adverse effects are being negated by creation of additional interactions.

The SAR for displacement of brevetoxin appears to be very different in nature to that exhibited for the brevenal receptor. For instance, the data for a compound such as the 3-methoxyphenylhydrazide **22** is confounding in that it is capable of displacing brevetoxin at very low concentrations (8 nM), but exhibits low affinity for the brevenal receptor (1 μM). If the displacement of brevetoxin is through allosteric interactions, then the compound should exhibit higher affinity for its native receptor, as in the case of brevenal. One explanation for this might be that the modifications made to brevenal enable the new molecule to bind at site five of voltage gated sodium channels, thereby directly competing with brevetoxin for its binding site. This hypothesis is somewhat supported by bronchoconstriction studies in sheep. This assay is used to determine if compounds have pulmonary irritant effects when inhaled [9]. The irritant effects, as evidenced by an increase in pulmonary resistance (R_L_), are for the most part short-lived, but are important because if a compound causes bronchoconstriction it is unlikely that it could be developed as an inhaled drug. To evaluate the current compounds, sheep were given 20 breaths of compounds **11**, **19**, **22** or **35** at doses of 10, 30 or 100 pg/mL. Compound **11**, with similar binding characteristics as brevenal, produced only a minimal 26% ± 2% (mean ± SD, *n* = 3) increase in R_L_ at 100 pg/mL. Further increases in concentration up to 50 μg /mL did not alter this response (22% ± 3%, *n* = 3). These results are consistent with those seen for brevenal. This finding suggests that the formyl hydrazide derivative is binding to the receptor in a similar manner as brevenal. However, for compounds **19**, **22** and **35**, significant increases in R_L_ were observed compared to compound **11** at the 100 pg/mL dose (R_L_ = 81% ± 4%, 96% ± 7% and 83% ± 4% respectively, mean ± SD, *n* = 3). While not as severe as results observed for brevetoxin (R_L_ = 221% ± 21% at 10 pg/mL) [9], these compounds are significantly more bronchoconstrictive than brevenal (no effects observed up to 50 μg/mL) [11]. Thus, based on these results, only compound **11** would be a candidate for development into an inhaled drug. The bronchoconstriction findings are highly consistent with the receptor affinity data generated for compound **22**. The results for compound **35** are less clear, as this compound retains fairly good affinity (231 nM) for the brevenal receptor compared to brevenal (137 nM). However, the fact that it still has a roughly two-fold higher affinity for the brevetoxin receptor (66 nM) may lead to the negative effects associated with brevetoxin receptor activation overriding any positive effects elicited by brevenal receptor activation. Even more puzzling though is the bronchoconstriction caused by compound **19**. Since this compound appears to have higher affinity for the brevenal receptor (412 nM) than brevetoxin displacement (1001 nM), it would be expected to have an opposite result to that observed for compound **35**. At this time, we can offer no explanation for this result.

## 3. Experimental Section

### 3.1. Brevenal Isolation

Brevenal was purified from cultures of *Karenia brevis* (Wilson strain) in similar fashion as previously described [4,5]. Briefly, *K. brevis* cultures were homogenized in the presence of chloroform. The chloroform layer was collected and evaporated under reduced pressure. The residue was partitioned between petroleum ether and a methanol-water mixture to remove pigments and lipid debris. The methanol-water fraction was evaporated under reduced pressure. The crude product was fractionated using counter-current chromatography using a water, methanol and acetonitrile mixture as the stationary phase, and a pentane-methylene chloride mixture as the mobile (descending) phase. The brevenal rich fractions were combined and evaporated before being subjected to a second counter current chromatography purification using a mixture of methanol, water and glyme as the stationary phase and xylene and pentane as the mobile (ascending) phase. The brevenal rich fractions were evaporated. The crude product was subjected to HPLC (Phenomonex phenyl-hexyl column: Isocratic elution, 96:4 methanol:water with detection at 215 and 290 nm). A final purification of the brevenal was achieved through HPLC (Dynamax C18; isocratic elution, 89:11 methanol:water with detection at 215 and 290 nm).

### 3.2. Hydrazide Intermediates

Hydrazides used in the synthesis of the following brevenal analogs were purchased from the following vendors and used as received: Hydrazides for compounds **11**, **21**, **29, 30** and intermediates used in the synthesis of compounds **28**, **31** and **33** were purchased from Alfa Aesar (Ward Hill, MA, USA); hydrazides for compounds **12**, **13**, **17**, **19**, **22**, **32** and intermediates used in the synthesis of compounds **34** and **35** were purchased from Sigma Aldrich, (St. Louis, MO, USA); hydrazides for compounds **15** and **20** were purchased from Acros, (Geel, Belgium); hydrazides for compounds **14**, **16** and **23** were purchased from Oakwood Chemicals, (Columbia, SC, USA); hydrazide for compound **18** and the hydroxybenzoic acid intermediate used in the synthesis of compounds **24**–**27** were purchased from TCI America (Portland, OR, USA). 

**Scheme 3:**
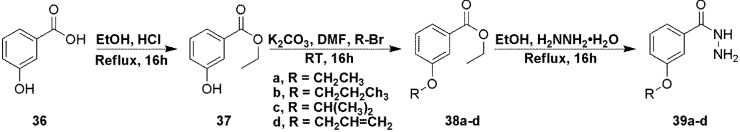
Preparation of hydrazide intermediates **39a**–**d**.

Hydrazides used in the synthesis of compounds **24**–**27** were prepared from 3-hydroxybenzoic acid (**36**) in three steps as shown in Scheme 3.

**Scheme 4:**
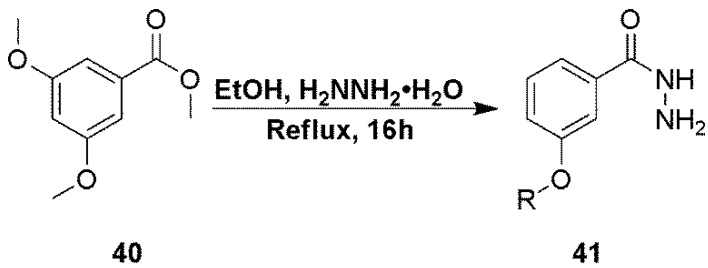
Preparation of hydrazide intermediate **41**.

3,5-Dimethoxyphenylbenzhydrazide (**41**), used in the preparation of compound **31** was prepared in a one step process from the methyl ester (**40**) as described in Scheme 4.

**Scheme 5:**
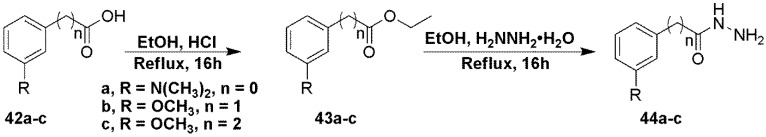
Preparation of hydrazide intermediates **44a-d**.

The hydrazides used in the synthesis of target compounds **31** and **33**–**35** were prepared from their acids in two steps as shown in Scheme 5.

### 3.3. Analytical Techniques

#### 3.3.1. LCMS

A low resolution LC/MS method was used to confirm the mass (M + 1) of all intermediates and products. The compounds were run under acidic conditions with the mobile phase consisting of acetonitrile (Honeywell Burdick and Jackson, Muskegon, MI, USA)–water (Fisher Scientific, Fair Lawn, NJ, USA)–formic acid (Fisher Scientific, Fair Lawn, NJ, USA) (80/20/0.1, v/v/v) at 100 μL/min over 3 min using an Agilent 1100 LC system equipped with a binary pump, autosampler and degasser (Agilent Technologies, Santa Clara, CA, USA) coupled to 2000 QTRAP mass spectrometer via an eletrospray ion (ESI) source (Turbospray) (Applied Biosystems, Foster City, CA, USA). The parameters for MS detection were scan type, Q1 MS scanning from 115 to 1700 amu, positive mode; cycle time, 1.0 s; duration, 2.965 min; cycles, 177; delay time, 0.0 s; scan mode, profile; step size, 0.1 amu; resolution Q1, unit; settling time, 0.0 ms; pause between mass ranges, 5.007 ms; curtain gas, 25 psi; ion spray voltage, 5000 V (positive); temperature, 300 °C; ion source gas 1, 35 psi; ion source gas 2, 50 psi; interface heater, on; declustering potential, 100 V; entrance potential, 11; and collision cell potential range, 8.9–63 V. Analyst software v1.4.1 (AB Sciex, Framingham, MA, USA) was used to run the following for the entire MS method: instrument control, data acquisition, and analysis.

#### 3.3.2. HRMS

Verification of target compound structures was verified using HRMS and ^1^H NMR. Prior to HRMS, separations were performed using an Agilent Eclipse plus C_18_ column (1.8 mm, 2.1 × 50 mm) (Agilent Technologies, Santa Clara, CA, USA) protected by an EC C_18_ guard column (2.7 μm, 2.1 × 5 mm) (Agilent Technologies, Santa Clara, CA, USA). An isocratic mobile phase gradient was used consisting of 80% MPB. Mobile phase A: 100% water (Fisher Scientific, Fair Lawn, NJ, USA) + 0.1% formic acid (Fisher Scientific, Fair Lawn, NJ, USA); mobile phase B: 100% LCMS Acetonitrile (Honeywell Burdick and Jackson, Muskegon, MI, USA) + 0.1% formic acid (Fisher Scientific, Fair Lawn, NJ, USA) attached to an Infinity 1290 UPLC compromising of a binary pump, a degasser, a diode array detector, thermostatted column compartment, and autosampler. HRMS of target compounds was performed on a Bruker micrOTOF-Q II HRMS (Bruker Biospin, Billerica, MA, USA) using an ESI source set in positive mode, using a dry heater setting of 200 °C, capillary voltage of 4000 V, nebulizer pressure of 3.0 Bar, and drying gas flow of 10.0 L/min. The end plate offset was set to −500 V, the collision cell RF was set to 60.0 Vpp and the scan with was set from 50 to 3000 *m/z*. The whole system was run by Hystar 3.2 (Bruker Biospin, Billerica, MA, USA) and the data were samples were processed and analyzed using Compass (Bruker Biospin, Billerica, MA, USA).

#### 3.3.3. NMR

^1^H NMR of all compounds was performed using a Bruker AVACE I 500 MHz instrument (Bruker Biospin, Billerica, MA fitted with a 1.7 mm TXI probe-head (Bruker Biospin, Billerica, MA, USA) optimized for ^1^H observations. The Bruker standard ZG pulse sequence was used for all compounds. Acquisition and processing of NMR data was performed using Topspin 2.1 patch level 6. One dimensional ^1^H NMR spectra were acquired for all compounds at 298 K and optimized for lock parameters, tune and match parameters, setting of receiver gain and shimming. All NMR solvents were purchased from Sigma Aldrich, St. Louis, MO, USA.

### 3.4. Receptor Binding Assays

Detailed descriptions of the methods used in this study can be found in [10]. 

#### Fluorescent-Ligand Binding Assays

To determine the inhibition of binding of the fluorescent ligand, competition binding experiments were performed as previously described [11]. Serial dilutions (1:10) of various competitors, ranging in concentration from 1 × 10^−12^ M to 1 × 10^−5^ M, were assayed for inhibition of binding of the fluorescent ligand (1 nM final concentration). The percent total binding curves were analyzed by non-linear regression analysis by GraphPad Prism v4.03 (GraphPad Software, Inc., La Jolla, CA, USA). Curves were analyzed by non-linear regression analysis, and equilibrium inhibition constants (*K_i_*) were determined using the *K_d_* value for the fluorescent ligands obtained in saturation experiments. Results presented are the mean ± SEM of at least three runs for each compound.

### 3.5. Sheep Bronchoconstriction Assay

Detailed descriptions of the methods used in this study can be found in [8]. 

#### 3.5.1. Animals

Adult ewes were used for this study. Animals were conscious, supported in a cart and intubated during the course of the experiments. All instrumentation was performed under local anesthesia. The study was conducted at Mount Sinai Medical Center under the approval of the Mount Sinai Medical Center Animal Research Committee. Pulmonary Resistance: Breath by breath measurements of pulmonary resistance (R_L_) were measured by the esophageal balloon technique. Analysis of 5–10 breaths was used to determine R_L_.

#### 3.5.2. Aerosols

Aerosols were generated using a Raindrop medication nebulizer. To control aerosol delivery a dosimetry system activated by a piston respirator was used. Nebulized aerosols were delivered directly into the tracheal tube only during inspiration at a tidal volume of 500 mL and at a frequency of 20 breaths/min. 

#### 3.5.3. Agents

Compounds were diluted to final experimental concentrations in 30% ETOH:70% 0.9% NaCl. 

#### 3.5.4. Protocol Airway Responses

Baseline R_L_ was measured and then the sheep were challenged with 20 breaths of compound. R_L_ was measured after each delivered concentration to assess the bronchoconstrictor activity. Values in the text are reported as mean ± SD increases in R_L_ over baseline for *n* = 3/group. A One Way Analysis of Variance followed by Student-Newman-Keuls Method to detect pairwise differences was used to determine if compounds produced a significant increase in R_L._

### 3.6. Synthetic Procedures

#### 3.6.1. Brevenal Derivatives

In a typical reaction, brevenal was dissolved in DMF and the hydrazide (2 eq) was added, followed by addition of a catalytic amount of tungstophosphoric acid. The reaction mixture was heated at 60 °C for 4 h. The solvents were evaporated under vacuum and the residue was taken up in methanol. The mixture was filtered through a 0.2 μm nylon filter and subjected to purification by HPLC. Desired products were positively identified by HRMS mass spectrometry and NMR. Spectroscopic data collected for all compounds can be found in the supplementary data document.

**Compound 11** was prepared by reaction of brevenal with formylhydrazide to give the product as a white solid in 71% yield. ^1^H NMR (CD_3_OD), δ 0.89 (t, *J* = 6 Hz, 1H), 0.96 (d, *J* = 6 Hz, 3H), 1.03 (s, 3H), 1.12 (s, 3H), 1.18 (s, 3H), 1.34 (m, 6H), 1.50 (m, 2H), 1.62 (m, 4H), 1.75 (m, 6H), 1.86 (m, 5H), 2.03 (m, 5H), 2.21 (m, 3H), 2.36 (m, 1H), 3.24 (m, 2H), 3.33 (m, 2H), 3.54 (br s, 1H), 3.71 (m, 1H), 3.96 (s, 1H), 4.06 (dd, *J* = 11 Hz and 5 Hz, 1H), 5.08 (d, *J* = 10 Hz, 1H), 5.18 (d, *J* = 17 Hz, 1H), 5.44 (q, 1H), 5.90 (dt, *J* = 16 Hz and 7 Hz, 1H), 6.03 (t, *J* = 11 Hz, 1H), 6.20 (d, *J* = 9 Hz, 1H), 6.69 (m, 1H), 8.10 (d, *J* = 10 Hz, 1H), 8.56 (s, 1H); HRMS calculated for C_40_H_63_N_2_O_8_ (M + H)^+^, 699.4579; found 699.4580.

**Compound 12** was prepared by reaction of brevenal with acetylhydrazide to give the product as a white solid in 52% yield. ^1^H NMR (CD_3_OD), δ 0.91 (t, *J* = 6 Hz, 1H), 0.97 (d, *J* = 7 Hz, 3H), 1.05 (s, 3H), 1.13 (s, 3H), 1.20 (s, 3H), 1.31 (m, 6H), 1.50 (m, 2H), 1.64 (m, 4H), 1.75 (m, 6H), 1.86 (m, 5H), 2.04 (m, 5H), 2.21 (m, 3H), 2.33 (m, 1H), 3.25 (m, 2H), 3.35 (m, 2H), 3.54 (m, 1H), 3.64 (s, 3H), 3.69 (t, 1H), 3.97 (m, 1H), 4.08 (m, 1H), 4.21 (m, 1H), 5.09 (d, *J* = 10 Hz, 1H), 5.19 (d, *J* = 17 Hz, 1H), 5.45 (m, 1H), 5.90 (m, 1H), 6.05 (m, 1H), 6.69 (m, 1H), 8.19 (d, *J* = 10 Hz, 1H); HRMS calculated for C_41_H_65_N_2_O_8_ (M + H)^+^, 713.4735; found 713.4729.

**Compound 13** was prepared by reaction of brevenal with propanoylhydrazide to give the product as a white solid in 39% yield. ^1^H NMR (CD_3_OD), δ 0.95 (d, *J* = 7 Hz, 3H), 1.03 (s, 3H), 1.12 (s, 3H), 1.16 (m, 5H), 1.27 (m, 5H), 1.36 (m, 2H), 1.50 (m, 2H), 1.64 (m, 4H), 1.76 (m, 6H), 1.86 (d, 5H), 2.03 (m, 5H), 2.14 (m, 1H), 2.25 (m, 4H), 2.35 (m, 1H), 2.63 (m, 1H), 3.23 (m, 2H), 3.32 (m, 2H), 3.53 (m, 1H), 3.70 (m, 1H), 3.95 (m, 1H), 4.06 (m, 1H), 5.07 (d, *J* = 10 Hz, 1H), 5.17 (dd, *J* = 16 Hz and 2 Hz, 1H), 5.43 (m, 1H), 5.86 (m, 1H), 6.02 (m, 1H), 6.32 (d, *J* = 10 Hz, 1H), 6.68 (m, 1H), 8.19 (d, *J* = 10 Hz, 1H); HRMS calculated for C_42_H_67_N_2_O_8_ (M + H)^+^, 727.4892; found 727.4885.

**Compound 14** was prepared by reaction of brevenal with isobutyrohydrazide to give the product as a white solid in 64% yield. ^1^H NMR (CD_3_OD), δ 0.69 (s, 1H), 0.89 (t, *J* = 7 Hz, 3H), 0.95 (d, *J* = 7 Hz, 3H), 1.02 (s, 3H), 1.10 (s, 3H), 1.14 (s, 3H), 1.16 (s, 3H), 1.17 (s, 3H), 1.62 (m, 4H), 1.74 (m, 6H), 1.86 (m, 5H), 2.02 (m, 5H), 2.24 (m, 3H), 2.31 (m, 3H), 3.24 (m, 2H), 3.31 (m, 4H), 3.52 (m, 1H), 3.67 (m, 3H), 3.95 (m, 1H), 4.18 (dd, *J* = 10 Hz and 5 Hz, 2H), 5.07 (d, *J* = 10 Hz, 1H), 5.17 (d, *J* = 16 Hz, 1H), 5.87 (m, 1H), 6.02 (m, 1H), 6.31 (d, *J* = 10 Hz, 1H), 6.67 (m, 1H), 8.22 (d, *J* = 9 Hz, 1H); HRMS calculated for C_43_H_69_N_2_O_8_ (M + H)^+^, 741.5048; found 741.5055.

**Compound 15** was prepared by reaction of brevenal with pivaloylhydrazide to give the product as a white solid in 84% yield. ^1^H NMR (CD_3_OD), δ 0.95 (d, *J* = 7 Hz, 3H), 1.02 (s, 3H), 1.11 (s, 3H), 1.18 (s, 3H), 1.23 (s, 9H), 1.28 (m, 2H), 1.35 (m, 3H), 1.50 (m, 2H), 1.62 (m, 3H), 1.74 (m, 6H), 1.84 (m, 5H), 2.03 (m, 6H), 2.23 (m, 3H), 3.23 (m, 2H), 3.30 (m, 5H), 3.53 (m, 1H), 3.70 (m, 1H), 3.96 (t, *J* = 4 Hz, 1H), 4.05 (m, 1H), 5.07 (d, *J* = 10 Hz, 1H), 5.17 (d, *J* = 16 Hz, 1H), 5.43 (m, 1H), 5.88 (t, *J* = 7 Hz, 1H), 6.02 (t, *J* = 11 Hz, 1H), 6.33 (d, *J* = 10 Hz, 1H), 6.68 (dt, *J* = 17 Hz and 10 Hz, 1H), 8.39 (d, *J* = 10 Hz, 1H); HRMS calculated for C_44_H_71_N_2_O_8_ (M + H)^+^, 755.5205; found 755.5226.

**Compound 16** was prepared by reaction of brevenal with cyclohexanoylhydrazide to give the product as a white solid in 55% yield. ^1^H NMR (CD_3_OD), δ 0.93 (d, 3H), 1.01 (s, 3H), 1.10 (s, 3H), 1.16 (s, 3H), 1.32 (m, 9H), 1.48 (m, 4H), 1.61 (m, 4H), 1.70–1.80 (m, 9H), 1.84 (m, 7H), 2.01 (m, 7H), 2.21 (m, 5H), 3.21 (m, 2H), 3.51 (m, 1H), 3.67 (m, 1H), 3.93 (m, 2H), 4.03 (m, 1H), 5.05 (d, *J* = 11 Hz, 1H), 5.15 (d, *J* = 17 Hz, 1H), 5.41 (m, 1H), 5.85 (m, 1H), 6.00 (t, *J* = 12 Hz, 1H), 6.30 (d, *J* = 10 Hz, 1H), 6.67 (m, 1H), 8.18 (d, *J* = 10 Hz, 1H); HRMS calculated for C_46_H_73_N_2_O_8_ (M + H)^+^, 781.5361; found 781.5360.

**Compound 17** was prepared by reaction of brevenal with benzoylhydrazide to give the product as a white solid in 45% yield. ^1^H NMR (CD_3_OD), δ 0.95 (d, *J* = 7 Hz, 3H), 1.02 (s, 3H), 1.11 (s, 3H), 1.18 (s, 3H), 1.30 (m, 3H), 1.36 (m, 3H), 1.50 (m, 2H), 1.61 (m, 3H), 1.76 (m, 6H), 1.87 (m, 5H), 2.04 (m, 6H), 2.25 (m, 4H), 3.23 (m, 2H), 3.33 (m, 3H), 3.54 (m, 1H), 3.70 (m, 1H), 3.96 (s, 1H), 4.06 (m, 1H), 5.07 (d, *J* = 11 Hz, 1H), 5.17 (dd, *J* = 17 Hz and 2 Hz, 1H), 5.42 (m, 1H), 5.92 (m, 1H), 6.01 (t, *J* = 11 Hz, 1H), 6.40 (d, *J* = 10 Hz, 1H), 6.67 (m, 1H), 7.49 (t, *J* = 7 Hz, 2H), 7.59 (t, *J* = 7 Hz, 1H), 7.87 (d, *J* = 7 Hz, 2H), 8.47 (d, *J* = 10 Hz, 1H); HRMS calculated for C_46_H_67_N_2_O_8_ (M + H)^+^, 775.4892; found 775.4892.

**Compound 18** was prepared by reaction of brevenal with picalinoylhydrazide to give the product as a pale yellow solid in 43% yield. ^1^H NMR (CD_3_OD), δ 0.96 (d, *J* = 6 Hz, 3H), 1.02 (s, 3H), 1.11 (s, 3H), 1.18 (s, 3H), 1.29 (m, 6H), 1.37 (m, 3H), 1.62 (m, 3H), 1.76 (m, 5H), 1.87 (m, 5H), 2.05 (m, 6H), 2.13 (m, 2H), 2.24 (m, 3H), 3.24 (m, 2H), 3.32 (m, 2H), 3.54 (m, 1H), 3.71 (m, 1H), 3.96 (m, 1H), 4.06 (m, 1H), 5.07 (d, *J* = 10 Hz, 1H), 5.17 (d, *J* = 18 Hz, 1H), 5.42 (m, 1H), 5.94 (m, 1H), 6.02 (m, 1H), 6.42 (d, *J* = 9 Hz, 1H), 6.68 (m, 1H), 7.57 (m, 1H), 7.97 (m, 1H), 8.15 (m, 1H), 8.60 (d, *J* = 10 Hz, 1H), 8.65 (d, *J* = 5 Hz, 1H); HRMS calculated for C_45_H_66_N_3_O_8_ (M+H)^+^, 776.4844; found 776.4830.

**Compound 19** was prepared by reaction of brevenal with nicotinoylhydrazide to give the product as a pale yellow solid in 32% yield. ^1^H NMR (CD_3_OD), δ 0.95 (d, *J* = 7 Hz, 3H), 1.02 (s, 3H), 1.11 (s, 3H), 1.18 (s, 3H), 1.37 (m, 2H), 1.50 (m, 2H), 1.62 (m, 3H), 1.75 (m, 7H), 1.85 (m, 5H), 2.02 (m, 6H), 2.15 (m, 1H), 2.25 (m, 3H), 2.35 (m, 1H), 3.24 (m, 2H), 3.32 (m, 5H), 3.53 (m, 1H), 3.70 (m, 1H), 3.96 (s, 1H), 4.05 (m, 1H), 5.07 (d, *J* = 10 Hz, 1H), 5.17 (d, *J* = 16 Hz, 1H), 5.43 (m, 1H), 5.94 (t, *J* = 8 Hz, 1H), 6.02 (t, *J* = 11 Hz, 1H), 6.40 (d, *J* = 10 Hz, 1H), 6.68 (m, 1H), 7.57 (dd, *J* = 8 Hz and 5 Hz, 1H), 8.30 (d, *J* = 8 Hz, 1H), 8.47 (d, *J* = 10 Hz, 1H), 8.71 (d, *J* = 5 Hz, 1H), 9.03 (s, 1H); HRMS calculated for C_45_H_66_N_3_O_8_ (M + H)^+^, 776.4844; found 776.4849.

**Compound 20** was prepared by reaction of brevenal with isonicatinoylhydrazide to give the product as a pale yellow solid in 84% yield. ^1^H NMR (CD_3_OD), δ 0.94 (d, *J* = 7 Hz, 3H), 1.01 (s, 3H), 1.10 (s, 3H), 1.17 (s, 3H), 1.26 (m, 5H), 1.35 (m, 3H), 1.50 (m, 2H), 1.60 (m, 3H), 1.74 (m, 6H), 1.84 (m, 4H), 2.01 (m, 5H), 2.12 (m, 1H), 2.23 (m, 2H), 2.32 (m, 1H), 3.22 (m, 2H), 3.31 (m, 3H), 3.52 (m, 1H), 3.69 (m, 1H), 3.94 (t, *J* = 4 Hz, 1H), 4.04 (m, 1H), 5.05 (d, *J* = 10 Hz, 1H), 5.15 (d, *J* = 17 Hz, 1H), 5.41 (m, 1H), 5.93 (t, *J* = 7 Hz, 1H), 6.00 (t, *J* = 11 Hz, 1H), 6.39 (d, *J* = 9 Hz, 1H), 6.67 (m, 1H), 7.83 (d, *J* = 5 Hz, 2H), 8.49 (d, *J* = 7 Hz, 1H), 8.70 (d, *J* = 5 Hz, 2H); HRMS calculated for C_45_H_66_N_3_O_8_ (M + H)^+^, 776.4844; found 776.4839.

**Compound 21** was prepared by reaction of brevenal with 2-methoxybenzoylhydrazide to give the product as a pale yellow solid in 78% yield. ^1^H NMR (CD_3_OD), δ 0.92 (d, *J* = 7 Hz, 3H), 0.99 (s, 3H), 1.08 (s, 3H), 1.15 (s, 3H), 1.27 (m, 6H), 1.35 (m, 2H), 1.46 (m, 2H), 1.59 (m, 4H), 1.72 (m, 5H), 1.84 (m, 5H), 1.98 (m, 5H), 2.12 (m, 1H), 2.21 (m, 2H), 2.31 (m, 1H), 3.20 (m, 1H), 3.29 (m, 3H), 3.50 (m, 1H), 3.68 (m, 1H), 3.93 (m, 4H), 4.02 (m, 1H), 5.04 (d, *J* = 10 Hz, 1H), 5.13 (d, *J* = 17 Hz, 1H), 5.40 (m, 1H), 5.88 (t, *J* = 7 Hz, 1H), 5.99 (t, *J* = 11 Hz, 1H), 6.37 (d, *J* = 10 Hz, 1H), 6.64 (m, 1H), 7.03 (t, *J* = 7 Hz, 1H), 7.11 (d, *J* = 8 Hz, 1H), 7.48 (m, 1H), 7.82 (dd, *J* = 8 Hz and 2 Hz, 1H), 8.36 (d, *J* = 10 Hz, 1H); HRMS calculated for C_47_H_69_N_2_O_9_ (M + H)^+^, 805.4998; found 805.5025.

**Compound 22** was prepared by reaction of brevenal with 3-methoxybenzoylhydrazide to give the product as a pale yellow solid in 58% yield. ^1^H NMR (CD_3_OD), δ 0.94 (d, *J* = 7 Hz, 3H), 1.01 (s, 3H), 1.10 (s, 3H), 1.17 (s, 3H), 1.25 (s, 8H), 1.49 (m, 2H), 1.61 (m, 4H), 1.73 (m, 5H), 1.84 (m, 3H), 2.01 (m, 5H), 2.13 (m, 1H), 2.23 (m, 3H), 2.33 (m, 1H), 3.21 (m, 2H), 3.31 (m, 3H), 3.52 (m, 1H), 3.70 (m, 1H), 3.83 (s, 3H), 3.94 (m, 1H), 4.03 (m, 1H), 4.55 (m, 1H), 5.06 (d, *J* = 10 Hz, 1H), 5.15 (d, *J* = 15 Hz, 1H), 5.41 (m, 1H), 5.90 (t, *J* = 8 Hz, 1H), 6.00 (t, *J* = 11 Hz, 1H), 6.39 (d, *J* = 10 Hz, 1H), 6.67 (m, 1H), 7.11 (dd, *J* = 8 Hz and 2 Hz, 1H), 7.37 (t, *J* = 8 Hz, 1H), 7.43 (m, 2H), 8.46 (d, *J* = 9 Hz, 1H); HRMS calculated for C_47_H_69_N_2_O_9_ (M + H)^+^, 805.4998; found 805.5007.

**Compound 23** was prepared by reaction of brevenal with 4-methoxybenzoylhydrazide to give the product as a pale yellow solid in 88% yield. ^1^H NMR (CD_3_OD), δ 0.92 (d, *J* = 7 Hz, 3H), 0.99 (s, 3H), 1.08 (s, 3H), 1.15 (s, 3H), 1.23 (m, 7H), 1.33 (m, 2H), 1.48 (m, 2H), 1.59 (m, 3H), 1.73 (m, 5H), 1.82 (m, 4H), 2.00 (m, 5H), 2.11 (m, 1H), 2.20 (m, 2H), 2.30 (m, 1H), 3.21 (m, 2H), 3.29 (m, 3H), 3.50 (m, 1H), 3.67 (m, 1H), 3.81 (s, 3H), 3.92 (t, *J* = 4 Hz, 1H), 4.02 (m, 1H), 5.04 (d, *J* = 10 Hz, 1H), 5.13 (dd, *J* = 17 Hz and 2 Hz, 1H), 5.40 (m, 1H), 5.87 (t, *J* = 7 Hz, 1H), 5.99 (t, *J* = 11 Hz, 1H), 6.37 (d, *J* = 10 Hz, 1H), 6.64 (m, 1H), 6.97 (d, *J* = 9 Hz, 2H), 7.83 (d, *J* = 9 Hz, 2H), 8.42 (d, *J* = 10 Hz, 1H); HRMS calculated for C_47_H_69_N_2_O_9_ (M + H)^+^, 805.4998; found 805.5009.

**Compound 24** was prepared by reaction of brevenal with 3-ethoxybenzoylhydrazide (**39a**) to give the product as a white solid in 41% yield. ^1^H NMR (CD_3_OD), δ 0.86 (t, *J* = 7 Hz, 2H), 0.94 (d, *J* = 7 Hz, 3H), 1.01 (s, 3H), 1.09 (s, 3H), 1.16 (s, 3H), 1.27 (m, 9H), 1.38 (t, *J* = 7 Hz, 3H), 1.49 (m, 1H), 1.59 (m, 3H), 1.74 (m, 4H), 1.83 (m, 4H), 2.00 (m, 6H), 2.13 (m, 2H), 2.24 (m, 2H), 2.34 (m, 1H), 3.22 (m, 2H), 3.31 (m, 2H), 3.52 (m, 1H), 3.69 (m, 1H), 3.94 (m, 1H), 4.05 (m, 2H), 5.05 (d, *J* = 10 Hz, 1H), 5.15 (dd, *J* = 17 Hz and 1 Hz, 1H), 5.41 (m, 1H), 5.90 (m, 1H), 6.01 (m, 1H), 6.39 (d, *J* = 10 Hz, 1H), 6.66 (m, 1H), 7.08 (d, *J* = 8 Hz, 1H), 7.36 (t, *J* = 8 Hz, 1H), 7.41 (m, 2H), 8.45 (d, *J* = 10 Hz, 1H); HRMS calculated for C_48_H_71_N_2_O_9_ (M + H)^+^, 819.5154; found 819.5159.

**Compound 25** was prepared by reaction of brevenal with 3-propoxybenzoylhydrazide (**39b**) to give the product as a white solid in 64% yield. ^1^H NMR (CD_3_OD), δ 0.86 (t, *J* = 7 Hz, 2H), 0.94 (d, *J* = 7 Hz, 3H), 1.02 (m, 5H), 1.09 (s, 3H), 1.17 (s, 3H), 1.27 (m, 9H), 1.48 (m, 1H), 1.59 (m, 3H), 1.75 (m, 6H), 1.84 (m, 4H), 2.01 (m, 5H), 2.13 (m, 2H), 2.22 (m, 2H), 2.33 (m, 1H), 3.22 (m, 2H), 3.31 (m, 2H), 3.52 (m, 1H), 3.70 (m, 1H), 3.96 (m, 3H), 4.03 (m, 1H), 5.05 (d, *J* = 10 Hz, 1H), 5.15 (d, *J* = 16 Hz, 1H), 5.30 (t, *J* = 5 Hz, 1H), 5.40 (m, 1H), 5.90 (m, 1H), 6.00 (t, *J* = 11 Hz, 1H), 6.39 (d, *J* = 9 Hz, 1H), 6.67 (m, 1H), 7.09 (d, *J* = 8 Hz, 1H), 7.36 (t, *J* = 8 Hz, 1H), 7.41 (m, 2H), 8.46 (d, *J* = 10 Hz, 1H); HRMS calculated for C_49_H_73_N_2_O_9_ (M + H)^+^, 833.5311; found 833.5321.

**Compound 26** was prepared by reaction of brevenal with 3-isopropoxybenzoylhydrazide (**39c**) to give the product as a white solid in 41% yield. ^1^H NMR (CD_3_OD), δ 0.86 (t, *J* = 7 Hz, 2H), 0.94 (m, 3H), 1.01 (s, 3H), 1.10 (s, 3H), 1.16 (s, 3H), 1.25 (m, 7H), 1.30 (m, 8H), 1.49 (m, 1H), 1.59 (m, 3H), 1.72 (m, 4H), 1.83 (m, 4H), 2.00 (m, 5H), 2.13 (m, 2H), 2.22 (m, 2H), 3.22 (m, 2H), 3.30 (m, 2H), 3.52 (m, 1H), 3.68 (m, 1H), 3.94 (m, 1H), 4.04 (m, 1H), 4.64 (m, 1H), 5.05 (d, *J* = 10 Hz, 1H), 5.15 (d, *J* = 17 Hz, 1H), 5.31 (t, *J* = 5 Hz, 1H), 5.42 (m, 1H), 5.90 (t, *J* = 7 Hz, 1H), 6.00 (t, *J* = 10 Hz, 1H), 6.38 (d, *J* = 11 Hz, 1H), 6.66 (m, 1H), 7.08 (dd, *J* = 7 Hz and 2 Hz, 1H), 7.35 (t, *J* = 9 Hz, 1H), 7.40 (m, 2H), 8.45 (d, *J* = 10 Hz, 1H); HRMS calculated for C_49_H_73_N_2_O_9_ (M + H)^+^, 833.5311; found 833.5294.

**Compound 27** was prepared by reaction of brevenal with 3-(allyloxy)benzoylhydrazide (**39d**) to give the product as a white solid in 77% yield. ^1^H NMR (CD_3_OD), δ 0.94 (d, *J* = 6 Hz, 3H), 1.01 (s, 3H), 1.09 (s, 3H), 1.16 (s, 3H), 1.27 (m, 3H), 1.34 (m, 2H), 1.49 (m, 2H), 1.60 (m, 4H), 1.73 (m, 6H), 1.83 (m, 4H), 2.00 (m, 6H), 2.12 (m, 2H), 2.23 (m, 3H), 2.33 (m, 1H), 3.22 (m, 2H), 3.30 (m, 2H), 3.52 (m, 1H), 3.69 (m, 1H), 3.94 (m, 1H), 4.04 (m, 1H), 4.58 (d, *J* = 4 Hz, 2H), 5.05 (d, *J* = 9 Hz, 1H), 5.15 (d, *J* = 17 Hz, 1H), 5.23 (d, *J* = 11 Hz, 1H), 5.41 (m, 2H), 5.90 (t, *J* = 7 Hz, 1H), 6.04 (m, 2H), 6.38 (d, *J* = 10 Hz, 1H), 6.67 (m, 1H), 7.12 (d, *J* = 7 Hz, 1H), 7.37 (t, *J* = 6 Hz, 1H), 7.42 (br s, 2H), 8.45 (d, *J* = 10 Hz, 1H); HRMS calculated for C_49_H_71_N_2_O_9_ (M + H)^+^, 831.5154; found 831.5169.

**Compound 28** was prepared by reaction of brevenal with 3,5-dimethoxybenzoylhydrazide (**41**) to give the product as a pale yellow solid in 78% yield. ^1^H NMR (CD_3_OD), δ 0.92 (d, *J* = 7 Hz, 3H), 0.99 (s, 3H), 1.08 (s, 3H), 1.15 (s, 3H), 1.25 (m, 4H), 1.32 (m, 2H), 1.48 (m, 2H), 1.58 (m, 4H), 1.71 (m, 6H), 1.82 (m, 4H), 1.99 (m, 6H), 2.13 (m, 2H), 2.22 (m, 2H), 2.32 (m, 1H), 3.20 (m, 2H), 3.29 (m, 2H), 3.50 (m, 1H), 3.65 (m, 1H), 3.79 (s, 6H), 3.92 (br s, 1H), 4.02 (m, 1H), 5.04 (d, *J* = 10 Hz, 1H), 5.14 (d, 1H), 5.40 (m, 1H), 5.89 (m, 1H), 5.99 (t, *J* = 11 Hz, 1H), 6.36 (d, *J* = 10 Hz, 1H), 6.64 (m, 2H), 7.00 (s, 2H), 8.44 (d, *J* = 9 Hz, 1H); HRMS calculated for C_48_H_71_N_2_O_10_ (M + H)^+^, 835.5103; found 835.5109.

**Compound 29** was prepared by reaction of brevenal with 3-hydroxybenzoylhydrazide to give the product as a white solid in 36% yield. ^1^H NMR (CD_3_OD), δ 0.98 (d, *J* = 7 Hz, 3H), 1.05 (s, 3H), 1.14 (s, 3H), 1.21 (s, 3H), 1.32 (m, 9H), 1.39 (m, 2H), 1.54 (m, 2H), 1.65 (m, 4H), 1.77 (m, 5H), 1.88 (m, 4H), 2.05 (m, 5H), 2.29 (m, 4H), 3.26 (m, 2H), 3.35 (m, 2H), 3.56 (m, 1H), 3.72 (m, 1H), 3.98 (t, *J* = 4 Hz, 1H), 4.08 (m, 1H), 5.10 (d, *J* = 10 Hz, 1H), 5.19 (d, *J* = 18 Hz, 1H), 5.45 (m, 1H), 5.94 (m, 1H), 6.04 (t, *J* = 10 Hz, 1H), 6.42 (d, *J* = 10 Hz, 1H), 6.70 (m, 1H), 7.00 (m, 1H), 7.32 (m, 2H), 8.48 (t, *J* = 10 Hz, 1H); HRMS calculated for C_46_H_67_N_2_O_9_ (M + H)^+^, 791.4841; found 791.4818.

**Compound 30** was prepared by reaction of brevenal with 3-methylbenzoylhydrazide to give the product as a white solid in 78% yield. ^1^H NMR (CD_3_OD), δ 0.98 (d, *J* = 7 Hz, 3H), 1.05 (s, 3H), 1.14 (s, 3H), 1.21 (s, 3H), 1.30 (m, 3H), 1.39 (m, 2H), 1.54 (m, 2H), 1.64 (m, 4H), 1.78 (m, 6H), 1.87 (m, 5H), 2.05 (m, 6H), 2.18 (m, 1H), 2.28 (m, 3H), 2.36 (m, 1H), 2.43 (s, 3H), 3.26 (m, 2H), 3.35 (m, 2H), 3.57 (m, 1H), 3.73 (m, 1H), 3.98 (t, *J* = 4 Hz, 1H), 4.08 (m, 1H), 5.10 (d, *J* = 10 Hz, 1H), 5.19 (dd, *J* = 17 Hz and 2 Hz, 1H), 5.47 (m, 1H), 5.94 (t, *J* = 7 Hz, 1H), 6.05 (t, *J* = 11 Hz, 1H), 6.43 (d, *J* = 10 Hz, 1H), 6.71 (m, 1H), 7.40 (m, 2H), 7.69 (d, *J* = 7 Hz, 1H), 7.73 (s, 1H), 8.50 (d, *J* = 10 Hz, 1H); HRMS calculated for C_47_H_69_N_2_O_8_ (M + H)^+^, 789.5048; found 789.5065.

**Compound 31** was prepared by reaction of brevenal with 3-dimethylaminobenzoylhydrazide (**44a**) to give the product as a white solid in 60% yield. ^1^H NMR (CD_3_OD), δ 0.98 (d, *J* = 7 Hz, 3H), 1.05 (s, 3H), 1.14 (s, 3H), 1.21 (s, 3H), 1.29 (m, 3H), 1.39 (m, 2H), 1.53 (m, 2H), 1.64 (m, 4H), 1.77 (m, 6H), 1.88 (m, 5H), 2.04 (m, 6H), 2.16 (m, 1H), 2.28 (m, 3H), 2.38 (m, 1H), 3.01 (s, 6H), 3.26 (m, 2H), 3.34 (m, 2H), 3.57 (m, 1H), 3.74 (m, 1H), 3.98 (t, *J* = 4 Hz, 1H), 4.08 (m, 1H), 5.10 (d, *J* = 10 Hz, 1H), 5.19 (dd, *J* = 17 Hz and 2 Hz, 1H), 5.46 (m, 1H), 5.94 (t, *J* = 7 Hz, 1H), 6.05 (t, *J* = 11 Hz, 1H), 6.43 (dd, *J* = 10 Hz and 3 Hz, 1H), 6.70 (m, 1H), 6.98 (dd, *J* = 8 Hz and 2 Hz, 1H), 7.19 (d, *J* = 8 Hz, 1H), 7.27 (br s, 1H), 7.31 (t, *J* = 8 Hz, 1H), 8.51 (br s, 1H); HRMS calculated for C_48_H_72_N_3_O_8_ (M + H)^+^, 818.5314; found 818.5334.

**Compound 32** was prepared by reaction of brevenal with 3-fluorobenzoylhydrazide to give the product as a pale yellow solid in 63% yield. ^1^H NMR (CD_3_OD), δ 0.94 (d, *J* = 7 Hz, 3H), 1.01 (s, 3H), 1.10 (s, 3H), 1.17 (s, 3H), 1.27 (m, 3H), 1.36 (m, 2H), 1.49 (m, 2H), 1.61 (m, 4H), 1.73 (m, 6H), 1.86 (m, 5H), 2.01 (m, 6H), 2.12 (m, 1H), 2.24 (m, 3H), 2.34 (m, 1H), 3.23 (m, 2H), 3.31 (m, 2H), 3.52 (m, 1H), 3.69 (m, 1H), 3.94 (t, *J* = 4 Hz, 1H), 4.04 (m, 1H), 5.06 (d, *J* = 10 Hz, 1H), 5.15 (dd, *J* = 17 Hz and 2 Hz, 1H), 5.41 (m, 1H), 5.91 (t, *J* = 7 Hz, 1H), 6.01 (t, *J* = 11 Hz, 1H), 6.39 (d, *J* = 10 Hz, 1H), 6.66 (dt, *J* = 17 Hz and 11 Hz, 1H), 7.30 (m, 1H), 7.50 (m, 1H), 7.61 (d, *J* = 8 Hz, 1H), 7.70 (d, *J* = 7 Hz, 1H), 8.46 (d, *J* = 10 Hz, 1H); HRMS calculated for C_46_H_66_FN_2_O_8_ (M + H)^+^, 793.4798; found 793.4807.

**Compound 33** was prepared by reaction of brevenal with 3-chlorobenzoylhydrazide (**44b**) to give the product as a white solid in 35% yield. ^1^H NMR (CD_3_OD), δ 0.98 (d, *J* = 7 Hz, 3H), 1.05 (s, 3H), 1.14 (s, 3H), 1.21 (s, 3H), 1.30 (m, 3H), 1.38 (m, 2H), 1.54 (m, 2H), 1.65 (m, 4H), 1.77 (m, 6H), 1.89 (m, 5H), 2.05 (m, 6H), 2.17 (m, 1H), 2.28 (m, 3H), 2.38 (m, 1H), 3.27 (m, 2H), 3.34 (m, 2H), 3.56 (m, 1H), 3.73 (m, 1H), 3.99 (t, *J* = 4 Hz, 1H), 4.08 (m, 1H), 5.09 (d, *J* = 10 Hz, 1H), 5.19 (d, *J* = 17 Hz, 1H), 5.45 (m, 1H), 5.96 (t, *J* = 7 Hz, 1H), 6.05 (t, *J* = 11 Hz, 1H), 6.43 (d, *J* = 9 Hz, 1H), 6.70 (m, 1H), 7.51 (t, *J* = 8 Hz, 1H), 7.61 (d, *J* = 7 Hz, 1H), 7.84 (d, *J* = 8 Hz, 1H), 7.93 (t, *J* = 2 Hz, 1H), 8.50 (d, *J* = 10 Hz, 1H); HRMS calculated for C_46_H_66_ClN_2_O_8_ (M + H)^+^, 809.4502; found 809.4497.

**Compound 34** was prepared by reaction of brevenal with 2-(3-methoxyphenyl)acetohydrazide (**44c**) to give the product as a white solid in 68% yield. ^1^H NMR (CD_3_OD), δ 0.97 (d, *J* = 7 Hz, 3H), 1.05 (s, 3H), 1.14 (s, 3H), 1.20 (s, 3H), 1.33 (m, 3H), 1.38 (m, 2H), 1.53 (m, 2H), 1.64 (m, 4H), 1.77 (m, 6H), 1.88 (m, 5H), 2.05 (m, 6H), 2.15 (m, 1H), 2.25 (m, 3H), 2.38 (m, 1H), 3.26 (m, 2H), 3.33 (m, 2H), 3.54 (m, 3H), 3.72 (m, 1H), 3.79 (s, 3H), 3.98 (d, *J* = 8 Hz, 1H), 4.08 (m, 1H), 5.10 (d, *J* = 10 Hz, 1H), 5.19 (dd, *J* = 17 Hz and 1 Hz, 1H), 5.45 (m, 1H), 5.91 (m, 1H), 6.05 (t, *J* = 11 Hz, 1H), 6.34 (d, *J* = 10 Hz, 1H), 6.70 (m, 1H), 6.81 (m, 1H), 6.90 (m, 2H), 7.21 (m, 1H), 8.27 (d, *J* = 10 Hz, 1H); HRMS calculated for C_48_H_71_N_2_O_9_ (M + H)^+^, 819.5154; found 819.5140.

**Compound 35** was prepared by reaction of brevenal with 3-(3-methoxyphenyl)propanehydrazide (**44d**) to give the product as a white solid in 50% yield. ^1^H NMR (CD_3_OD), δ 0.97 (d, *J* = 7 Hz, 3H), 1.05 (s, 3H), 1.14 (s, 3H), 1.20 (s, 3H), 1.27 (m, 2H), 1.39 (m, 3H), 1.52 (m, 2H), 1.65 (m, 4H), 1.76 (m, 6H), 1.87 (m, 5H), 2.04 (m, 6H), 2.16 (m, 1H), 2.26 (m, 3H), 2.39 (m, 1H), 2.54 (t, *J* = 8 Hz, 2H), 2.95 (m, 3H), 3.22 (m, 2H), 3.34 (m, 2H), 3.55 (m, 1H), 3.72 (m, 1H), 3.77 (s, 3H), 3.98 (dd, *J* = 5 Hz and 1 Hz, 1H), 4.08 (m, 1H), 5.10 (d, *J* = 10 Hz, 1H), 5.19 (dd, *J* = 17 Hz and 2 Hz, 1H), 5.47 (m, 1H), 5.89 (m, 1H), 6.05 (t, *J* = 11 Hz, 1H), 6.33 (d, *J* = 10 Hz, 1H), 6.72 (m, 1H), 6.80 (m, 2H), 7.18 (m, 1H), 8.16 (m, 1H); HRMS calculated for C_49_H_73_N_2_O_9_ (M + H)^+^, 833.5311; found 833.5289.

#### 3.6.2. Hydrazide Intermediates

**Compound 37**. To a solution of 3-hydroxybenzoic acid (5 g, 36.2 mmol) in ethanol (30 mL) was added cHCl (5 drops) and the mixture heated at reflux overnight. The solvents were evaporated to give the target compound as a white solid (6.02 g, 100%). ^1^H NMR (CD_3_OD), δ 1.37 (t, *J* = 7 Hz, 2H), 4.33 (q, *J* = 7 Hz, 3H), 7.00 (dt, *J* = 8 Hz and 3 Hz, 1H), 7.26 (dt, *J* = 8 Hz and 2 Hz, 1H), 7.42 (dt, *J* = 6 Hz and 2 Hz, 1H), 7.48 (dd, *J* = 6 Hz and 1 Hz, 1H).

**Compound 38a**. To a solution of compound 37 (1 g, 6.02 mmol) in DMF (10 mL) was added potassium carbonate (2.5 g, 18.05 mmol) and ethylbromide (2.24 mL, 30.09 mmol) and the mixture was stirred at room temperature overnight. The crude mixture was then poured into water and extracted with hexanes. The organic extracts were washed with water, brine, dried over anhydrous sodium sulfate, filtered and evaporated to give the desired product as a colorless oil (1.121 g, 95%). Product was used without further purification. ^1^H NMR (CDCl_3_), δ 1.40 (t, *J* = 7 Hz, 3H), 1.44 (t, *J* = 7 Hz, 3H), 4.08 (q, *J* = 7 Hz, 2H), 4.38 (q, *J* = 7 Hz, 2H), 7.09 (dd, *J* = 8 Hz and 2 Hz, 1H), 7.33 (t, *J* = 8 Hz, 1H), 7.56 (br s, 1H), 7.63 (d, *J* = 8 Hz, 1H).

**Compound 38b** was prepared in a similar manner as compound **38a** using 1-bromopropane to give the product as a colorless oil in 93% yield. ^1^H NMR (CDCl_3_), δ 1.02 (m, 6H), 1.80 (m, 4H), 4.35 (q, *J* = 7 Hz, 2H), 7.07 (dd, *J* = 8 Hz and 2 Hz, 1H), 7.31 (t, *J* = 8 Hz, 1H), 7.55 (d, *J* = 2 Hz, 1H), 7.61 (d, *J* = 8 Hz, 1H).

**Compound 38c** was prepared in a similar manner as compound **38a** using 2-bromopropane to give the product as a colorless oil in 86% yield. ^1^H NMR (CDCl_3_), δ 1.34 (t, *J* = 7 Hz, 6H), 1.37 (t, *J* = 7 Hz, 3H), 4.35 (q, *J* = 7 Hz, 2H), 4.59 (m, 1H), 7.05 (dd, *J* = 8 Hz and 3 Hz, 1H), 7.30 (m, 1H), 7.54 (br s, 1H), 7.59 (dd, *J* = 8 Hz and 4 Hz, 1H).

**Compound 38d** was prepared in a similar manner as compound **38a** using allylbromide to give the product as a pale yellow oil in 63% yield. ^1^H NMR (CDCl_3_), δ 1.38 (t, *J* = 7 Hz, 3H), 4.36 (q, *J* = 7 Hz, 2H), 4.57 (d, *J* = 5 Hz, 1H), 5.29 (m, 1H), 5.41 (m, 1H), 6.04 (m, 1H), 7.11 (dt, *J* = 9 Hz and 3 Hz, 1H), 7.33 (m, 1H), 7.58 (m, 1H), 7.64 (dd, *J* = 8 Hz and 3 Hz, 1H).

**Compound 39a**. To a solution of compound **38a** (500 mg, 2.57 mmol) in ethanol (5 mL) was added hydrazine hydrate (1.25 mL, 25.74 mmol) and the mixture was heated at reflux overnight. The solvents were evaporated and the crude mixture was partitioned between water and chloroform. The aqueous was extracted with chloroform and the combined organic extracts were washed with water and brine, dried over anhydrous sodium sulfate, filtered and evaporated to give the desired product as a white solid (310 mg, 66%). Product was used without any further purification. ^1^H NMR (CD_3_OD), δ 1.40 (t, *J* = 7 Hz, 3H), 4.08 (q, *J* = 7 Hz, 2H), 7.07 (dt, *J* = 7 Hz and 2 Hz, 1H), 7.34 (m, 3H); MS *m/z* = 181.9 (M + H)^+^.

**Compound 39b** was prepared in similar fashion to compound **39a**, using compound **38b** and was isolated as a white solid in 85% yield. ^1^H NMR (CD_3_OD), δ 1.06 (t, *J* = 8 Hz, 2H), 1.84 (m, 2H), 3.98 (q, *J* = 6 Hz, 2H), 7.06 (d, *J* = 7 Hz, 1H), 7.37 (m, 1H), 7.41 (m, 1H), 7.45 (m, 1H); MS *m/z* = 195.8 (M + H)^+^.

**Compound 39c** was prepared in similar fashion to compound **39a**, using compound **38c** and was isolated as a white solid in 75% yield. ^1^H NMR (CD_3_OD), δ 1.17 (br s, 6H), 4.60 (m, 1H), 6.83 (d, *J* = 5 Hz, 1H), 7.04 (m, 1H), 7.29 (m, 1H), 7.37 (m, 1H); MS *m/z* = 195.9 (M + H)^+^.

**Compound 39d** was prepared in similar fashion to compound **39a**, using compound **38d** and was isolated as a white solid in 88% yield. ^1^H NMR (CD_3_OD), δ 4.58 (m, 2H), 5.31 (m, 1H), 5.42 (m, 1H), 6.04 (m, 1H), 7.06 (m, 1H), 7.36 (m, 2H), 7.43 (m, 1H). ); MS *m/z* = 193.9 (M + H)^+^. 

**Compound 41**. To a solution of compound 40 (1 g, 5.10 mmol) in methanol (10 mL) was added hydrazine hydrate (5 mL, 102 mmol) and the mixture was heated at reflux overnight. The solvents were evaporated and the crude mixture by flash chromatography using a gradient mixture of methanol in dichloromethane to give the desired product as a pale yellow solid (310 mg, 66%). Product was used without any further purification. ^1^H NMR (CDCl_3_), δ 3.81 (s, 6H), 6.58 (s, 1H), 6.88 (m, 2H); MS *m/z* = 197.5 (M + H)^+^.

**Compound 44a**. To a solution of 3-(dimethylamino)benzoic acid (1 g, 6.05 mmol) in ethanol (10 mL) was added cHCl (5 drops) and the mixture heated at reflux overnight. The solvents were evaporated to give the target compound as a white solid. This compound was then dissolved in ethanol (10 mL) and hydrazine hydrate (5.89 mL, 121 mmol) was added and the mixture heated at reflux overnight. Solvents were evaporated and the product was purified by flash chromatography using a gradient mixture of ethyl acetate and hexanes to give the desire compound as a white solid (1.02 g, 93% over 2 steps). ^1^H NMR (CDCl_3_), δ 2.95 (s, 6H), 6.84 (d, *J* = 8 Hz, 1H), 6.99 (d, *J* = 7 Hz, 1H), 7.19 (m, *J* = 20 Hz, 1H), 7.26 (m, *J* = 6 Hz, 1H); MS *m/z* = 180.6 (M + H)^+^.

**Compound 44b**. To a solution of 3-chlorobenzoic acid (1 g, 6.39 mmol) in ethanol (10 mL) was added cHCl (5 drops) and the mixture heated at reflux overnight. The solvents were evaporated to give the target compound as a white solid. This compound was then dissolved in ethanol (10 mL) and hydrazine hydrate (6.21 mL, 128 mmol) was added and the mixture heated at reflux overnight. Solvents were evaporated and the product was purified by flash chromatography using a gradient mixture of ethyl acetate and hexanes to give the desire compound as a yellow solid (890 mg, 81% over 2 steps). ^1^H NMR (CDCl_3_), δ 7.41 (t, *J* = 7 Hz, 1H), 7.49 (d, *J* = 7 Hz, 1H), 7.73 (m, 1H), 7.86 (m, *J* = 4 Hz, 1H); MS *m/z* = 171.5, 173.5 (M + H)^+^.

**Compound 44c**. To a solution of 3-methoxyphenylacetic acid (1 g, 6.02 mmol) in ethanol (10 mL) was added cHCl (5 drops) and the mixture heated at reflux overnight. The solvents were evaporated to give the target compound as a white solid. This compound was then dissolved in ethanol (10 mL) and hydrazine hydrate (5.86 mL, 120 mmol) was added and the mixture heated at reflux overnight. Solvents were evaporated and the product was purified by flash chromatography using a gradient mixture of ethyl acetate and hexanes to give the desire compound as a white solid (1.03 g, 94% over 2 steps). ^1^H NMR (CDCl_3_), δ 3.79 (s, 3H), 3.83 (s, 2H), 6.83 (m, 4H); MS *m/z* = 181.5 (M + H)^+^.

**Compound 44d**. To a solution of 3-methoxyphenylpropanoic acid (1 g, 5.55 mmol) in ethanol (10 mL) was added cHCl (5 drops) and the mixture heated at reflux overnight. The solvents were evaporated to give the target compound as a white solid. This compound was then dissolved in ethanol (10 mL) and hydrazine hydrate (5.83 mL, 119 mmol) was added and the mixture heated at reflux overnight. Solvents were evaporated and the product was purified by flash chromatography using a gradient mixture of ethyl acetate and hexanes to give the desire compound as a white solid (1.10 g, 94% over 2 steps). ^1^H NMR (CDCl_3_), δ 2.55 (m, 1H), 2.86 (m, 1H), 2.97 (m, 2H), 3.78 (s, 3H), 6.73 (d, *J* = 2 Hz, 1H), 6.77 (m, 2H), 7.21 (m, 1H); MS *m/z* = 195.6 (M + H)^+^.

## 4. Conclusions

We report the preparation of a series of brevenal hydrazide derivatives in an attempt to explore structure-activity within the brevenal receptor. From this series, it appears that only small modifications are tolerated, with only the formyl hydrazide retaining good affinity for the brevenal receptor and exhibiting low adverse effects in the sheep bronchoconstriction assay. Additionally, with the in-house findings that the hydrazide derivatives of brevenal appear to have good stability using *in vitro* testing, this compound is a strong candidate as a back-up or second generation analog for brevenal in development as a treatment for pulmonary disorders. Increasing the size of the substituent leads to a dramatic reduction in affinity for the brevenal receptor. Compounds capable of displacing brevetoxin at concentrations much lower than those needed for brevenal have also been prepared. However, these highly potent compounds cause bronchoconstriction in the sheep model at low concentrations, suggesting that they may act directly at the site of action for brevetoxin and not through previously described allosteric modulation of the brevetoxin receptor. Further studies are required to fully understand the findings reported here, and will be presented in due course. 

## Supplementary Files

Supplementary File 1 Supplementary Information (PDF, 3269 KB)
