# Supplementary material for: Structure Activity Relationship of Brevenal Hydrazide Derivatives
Source: Mar Drugs. 2014 Mar 28;12(4):1839–58. doi: 10.3390/md12041839 (PMC4012454; doi:10.3390/md12041839)
Supplement: Supplementary File 1 — Supplementary Information (PDF, 3269 KB) [file marinedrugs-12-01839-s001.pdf]

Supplementary Information

Figure S1. ^{1}H NMR spectrum of compound **11**.
Figure S2. HRMS spectrum of compound **11**.
Figure S3. ^{1}H NMR spectrum of compound **12**.
Figure S4. HRMS spectrum of compound **12**.
Figure S5. ^{1}H NMR spectrum of compound **13**.
Figure S6. HRMS spectrum of compound **13**.
Figure S7. ^{1}H NMR spectrum of compound **14**.
Figure S8. HRMS spectrum of compound **14**.
Figure S9. ^{1}H NMR spectrum of compound **15**.
Figure S10. HRMS spectrum of compound **15**.
Figure S11. ^{1}H NMR spectrum of compound **16**.
Figure S12. HRMS spectrum of compound **16**.
Figure S13. ^{1}H NMR spectrum of compound **17**.
Figure S14. HRMS spectrum of compound **17**.
Figure S15. ^{1}H NMR spectrum of compound **18**.
Figure S16. HRMS spectrum of compound **18**.
Figure S17. ^{1}H NMR spectrum of compound **19**.
Figure S18. HRMS spectrum of compound **19**.
Figure S19. ^{1}H NMR spectrum of compound **20**.
Figure S20. HRMS spectrum of compound **20**.
Figure S21. ^{1}H NMR spectrum of compound **21**.
Figure S22. HRMS spectrum of compound **21**.
Figure S23. ^{1}H NMR spectrum of compound **22**.
Figure S24. HRMS spectrum of compound **22**.
Figure S25. ^{1}H NMR spectrum of compound **23**.
Figure S26. HRMS spectrum of compound **23**.
Figure S27. ^{1}H NMR spectrum of compound **24**.
Figure S28. HRMS spectrum of compound **24**.
Figure S29. ^{1}H NMR spectrum of compound **25**.
Figure S30. HRMS spectrum of compound **25**.
Figure S31. ^{1}H NMR spectrum of compound **26**.
Figure S32. HRMS spectrum of compound **26**.
Figure S33. ^{1}H NMR spectrum of compound **27**.
Figure S34. HRMS spectrum of compound **27**.
Figure S35. ^{1}H NMR spectrum of compound **28**.
Figure S36. HRMS spectrum of compound **28**.
Figure S37. ^{1}H NMR spectrum of compound **29**.
Figure S38. HRMS spectrum of compound **29**.
Figure S39. ^{1}H NMR spectrum of compound **30**.
Figure S40. HRMS spectrum of compound **30**.
Figure S41. ^{1}H NMR spectrum of compound **31**.

Figure S42. HRMS spectrum of compound **31**.
Figure S43. ^{1}H NMR spectrum of compound **32**.
Figure S44. HRMS spectrum of compound **32**.
Figure S45. ^{1}H NMR spectrum of compound **33**.
Figure S46. HRMS spectrum of compound **33**.
Figure S47. ^{1}H NMR spectrum of compound **34**.
Figure S48. HRMS spectrum of compound **34**.
Figure S49. ^{1}H NMR spectrum of compound **35**.
Figure S50. HRMS spectrum of compound **35**.
Figure S51. ^{1}H NMR spectrum of compound **37**.
Figure S52. ^{1}H NMR spectrum of compound **38a**.
Figure S53. ^{1}H NMR spectrum of compound **38b**.
Figure S54. ^{1}H NMR spectrum of compound **38c**.
Figure S55. ^{1}H NMR spectrum of compound **38d**.
Figure S56. ^{1}H NMR spectrum of compound **39a**.
Figure S57. TIC and MS spectrum of compound **39a**.
Figure S58. ^{1}H NMR spectrum of compound **39b**.
Figure S59. TIC and MS spectrum of compound **39b**.
Figure S60. ^{1}H NMR spectrum of compound **39c**.
Figure S61. TIC and MS spectrum of compound **39c**.
Figure S62. ^{1}H NMR spectrum of compound **39d**.
Figure S63. TIC and MS spectrum of compound **39d**.
Figure S64. ^{1}H NMR spectrum of compound **41**.
Figure S65. TIC and MS spectrum of compound **41**.
Figure S66. ^{1}H NMR spectrum of compound **44a**.
Figure S67. TIC and MS spectrum of compound **44a**.
Figure S68. ^{1}H NMR spectrum of compound **44b**.
Figure S69. TIC and MS spectrum of compound **44b**.
Figure S70. ^{1}H NMR spectrum of compound **44c**.
Figure S71. TIC and MS spectrum of compound **44c**.
Figure S72. ^{1}H NMR spectrum of compound **44d**.
Figure S73. TIC and MS spectrum of compound **44d**.

Figure S1. ^{1}H NMR spectrum of compound **11**.

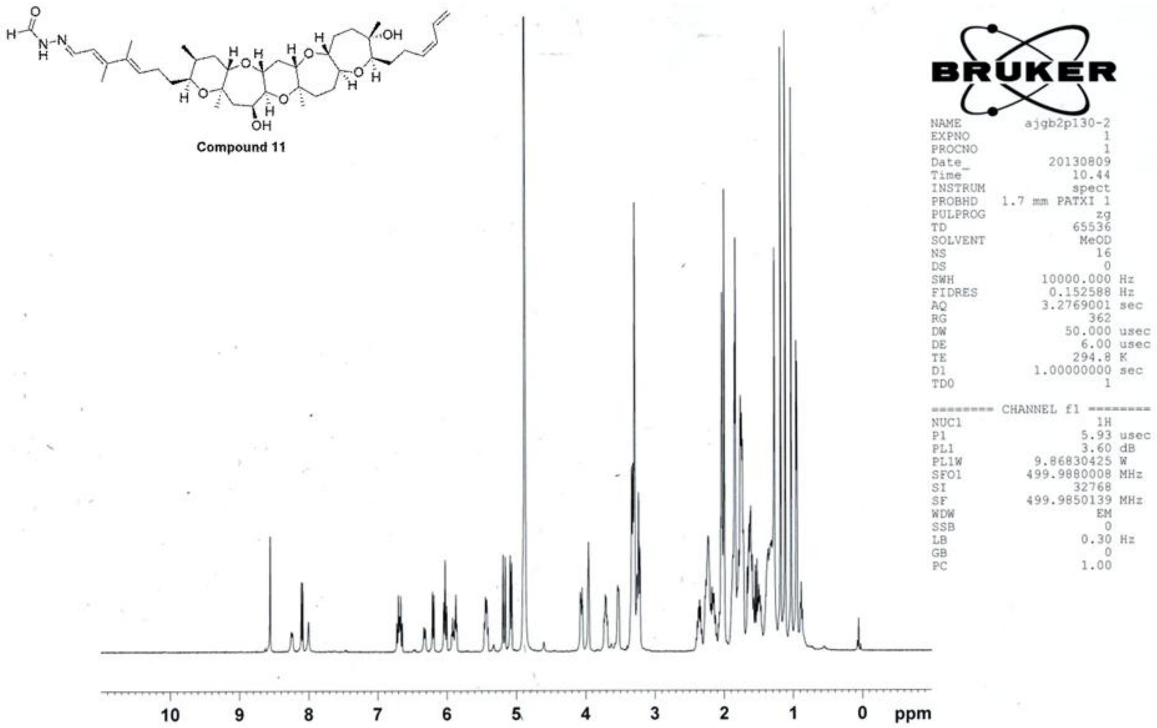

Figure S2. HRMS spectrum of compound **11**.

Mass Spectrum SmartFormula Report

Analysis Info

Analysis Name	D:\Data\Andrea\12_13_2013_brevenal cpds\130-3_P1-B-1_01_569.d	Acquisition Date	12/13/2013 12:13:43 PM
Method	brevenal.m	Operator	ADM
Sample Name	130-3	Instrument / Ser#	micrOTOF-Q II 10318
Comment			

Acquisition Parameter

Source Type	ESI	Ion Polarity	Positive	Set Nebulizer	3.0 Bar
Focus	Not active	Set Capillary	4000 V	Set Dry Heater	200 °C
Scan Begin	50 m/z	Set End Plate Offset	-500 V	Set Dry Gas	10.0 l/min
Scan End	3000 m/z	Set Collision Cell RF	600.0 Vpp	Set Divert Valve	Waste

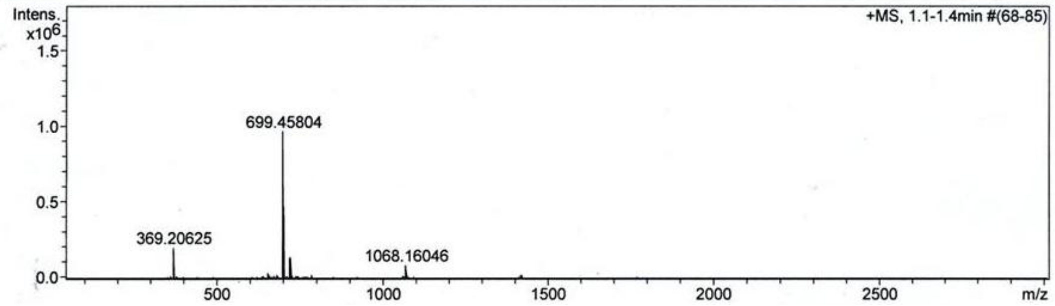

Figure S3. ^{1}H NMR spectrum of compound **12**.

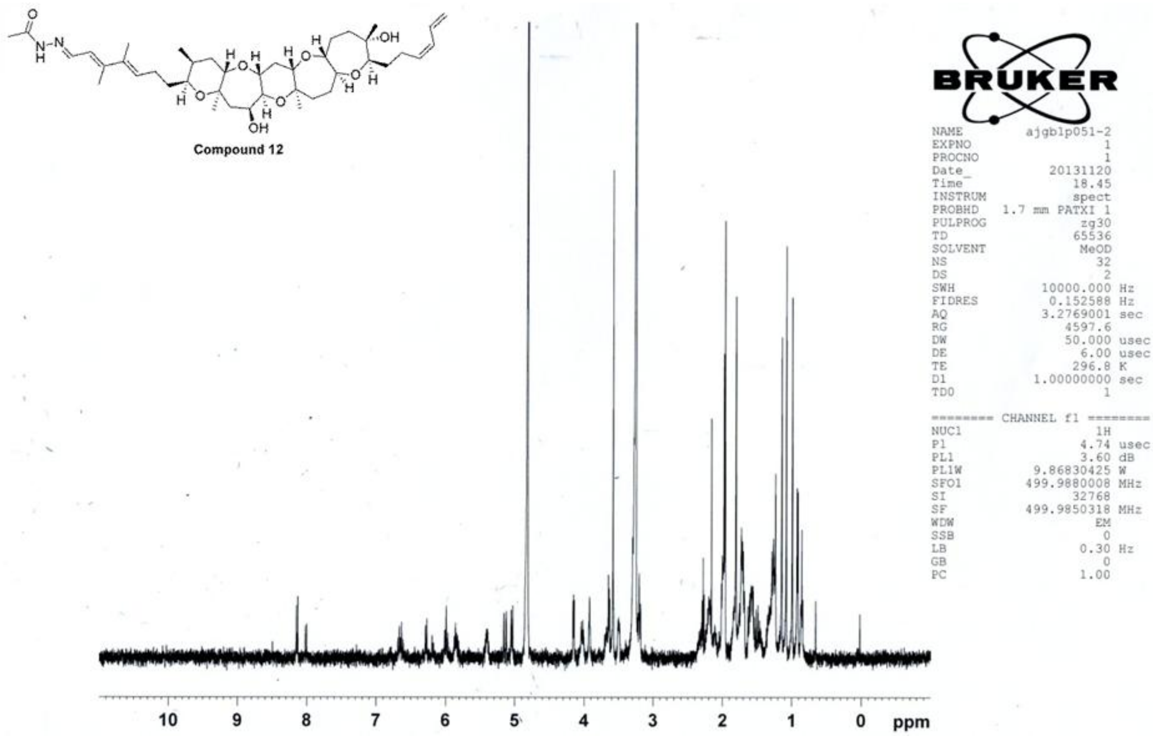

Figure S4. HRMS spectrum of compound **12**.

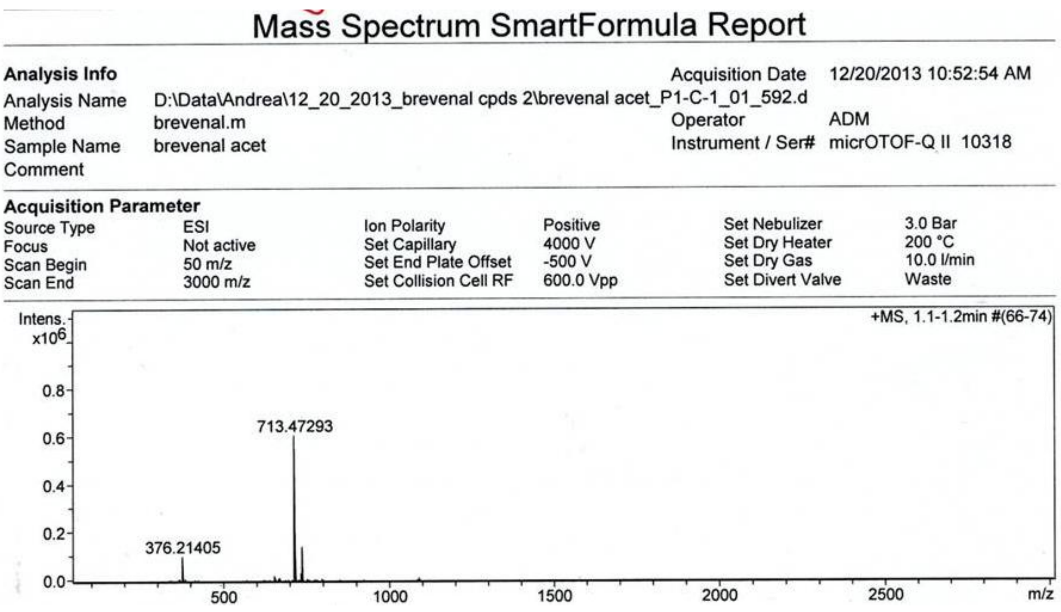

Figure S5. ^{1}H NMR spectrum of compound **13**.

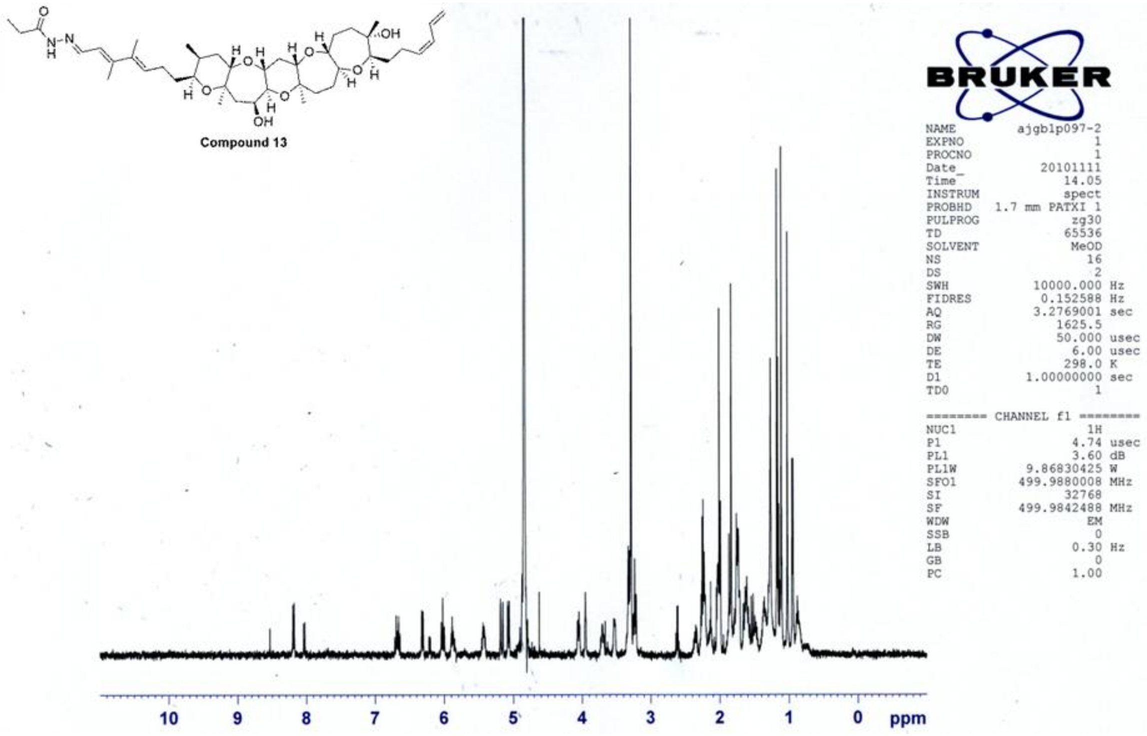

Figure S6. HRMS spectrum of compound **13**.

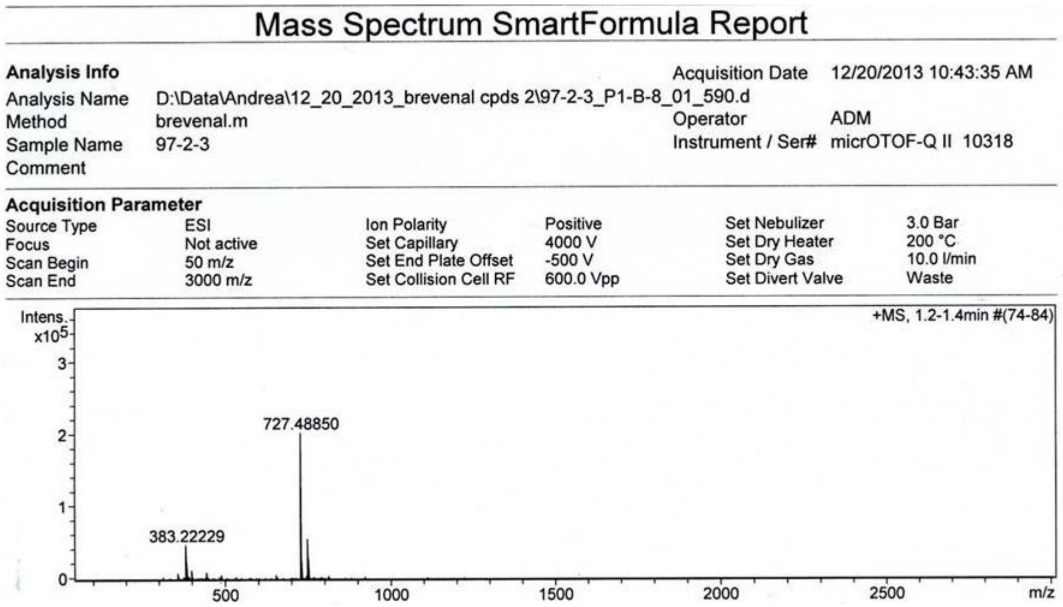

Figure S7. ^{1}H NMR spectrum of compound **14**.

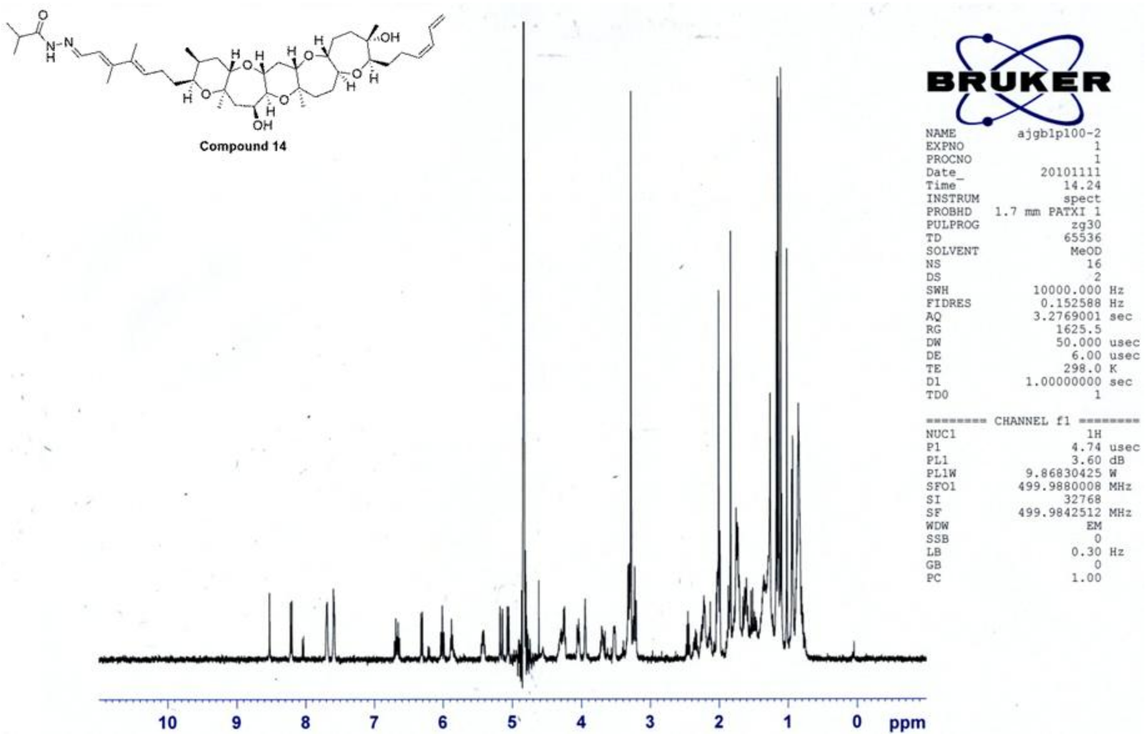

Figure S8. HRMS spectrum of compound **14**.

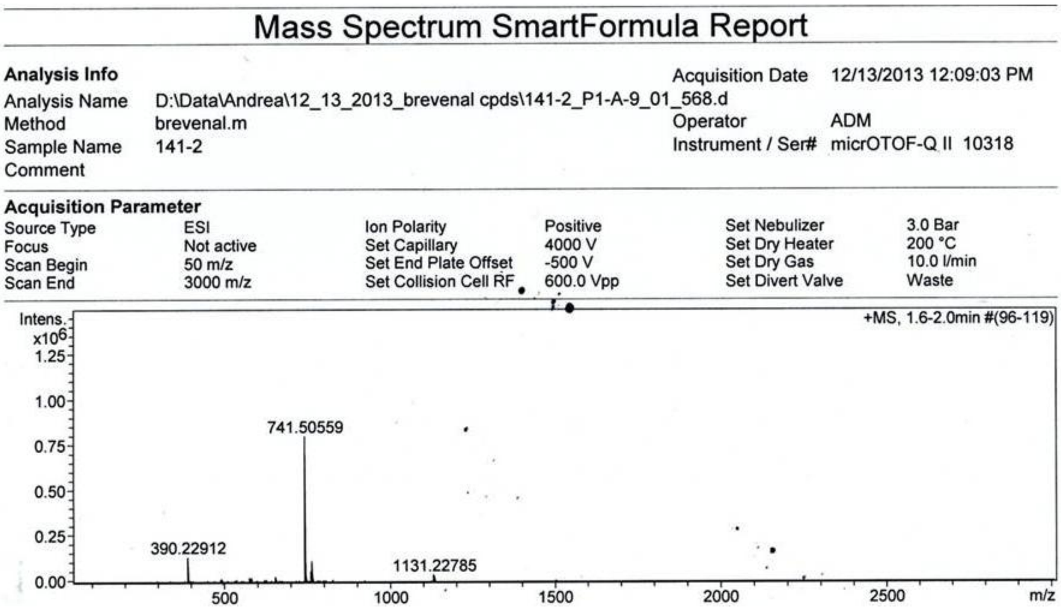

Figure S9. ^{1}H NMR spectrum of compound **15**.

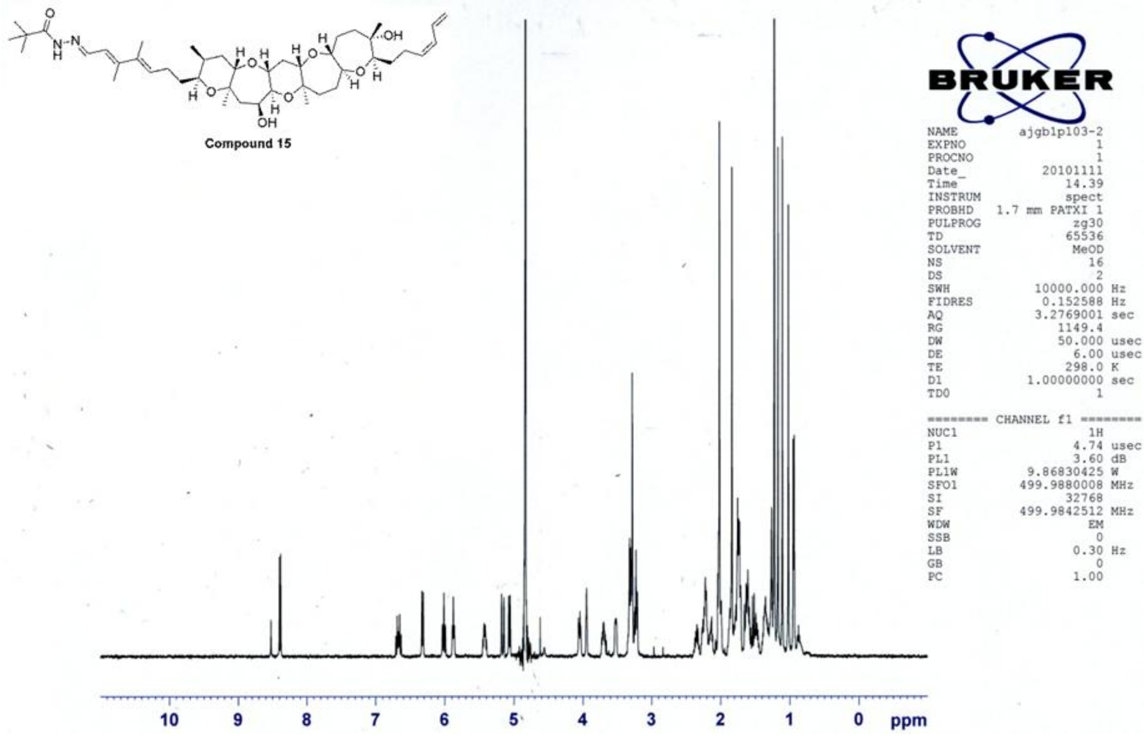

Figure S10. HRMS spectrum of compound **15**.

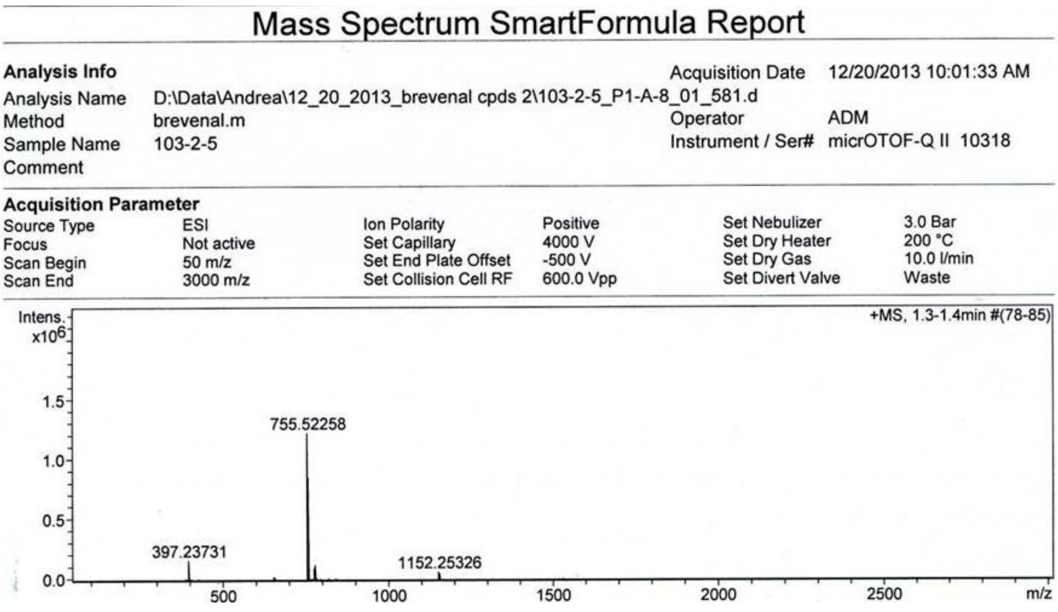

Figure S11. ^{1}H NMR spectrum of compound **16**.

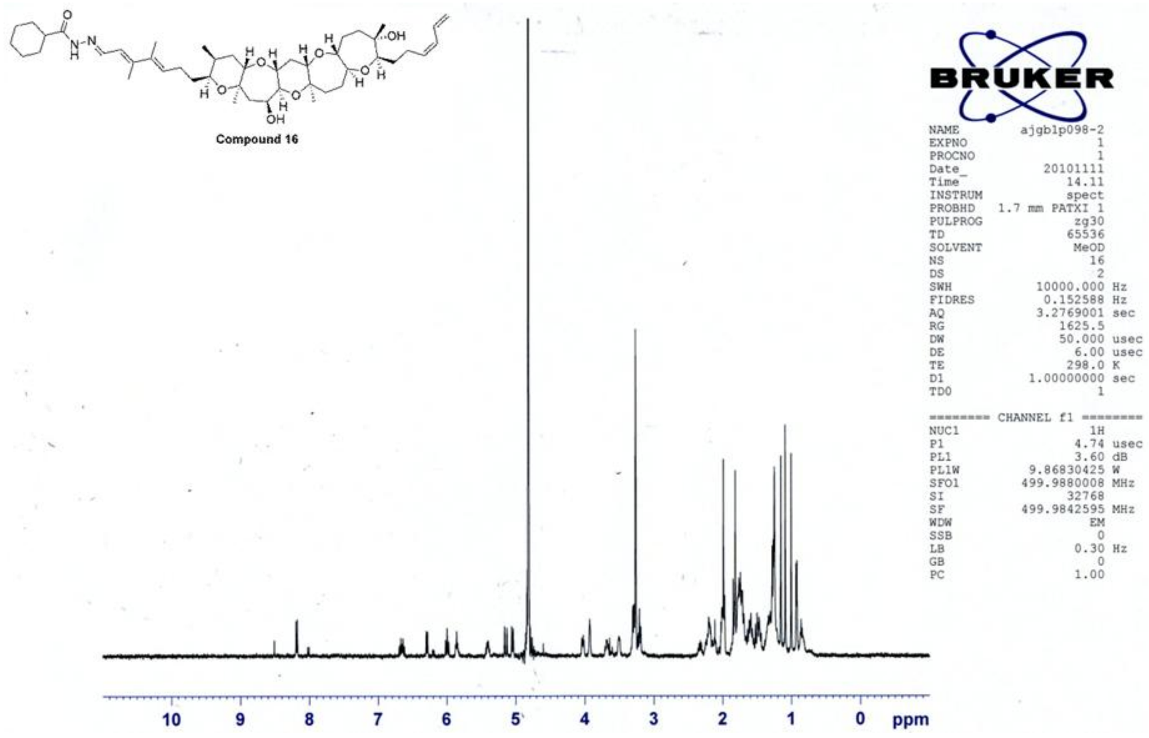

Figure S12. HRMS spectrum of compound **16**.

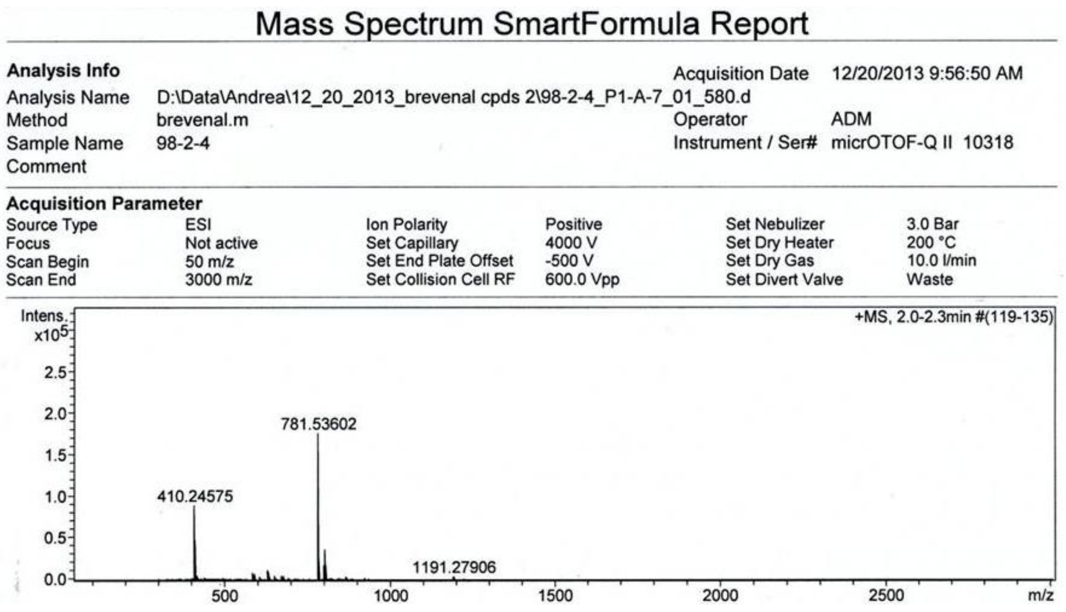

Mass Spectrum SmartFormula Report

Analysis Info

Analysis Name	D:\Data\Andrea\12_20_2013_brevenal cpds 2\98-2-4_P1-A-7_01_580.d	Acquisition Date	12/20/2013 9:56:50 AM
Method	brevenal.m	Operator	ADM
Sample Name	98-2-4	Instrument / Ser#	micrOTOF-Q II 10318
Comment			

Acquisition Parameter

Source Type	ESI	Ion Polarity	Positive	Set Nebulizer	3.0 Bar
Focus	Not active	Set Capillary	4000 V	Set Dry Heater	200 °C
Scan Begin	50 m/z	Set End Plate Offset	-500 V	Set Dry Gas	10.0 l/min
Scan End	3000 m/z	Set Collision Cell RF	600.0 Vpp	Set Divert Valve	Waste

Figure S13. ^{1}H NMR spectrum of compound **17**.

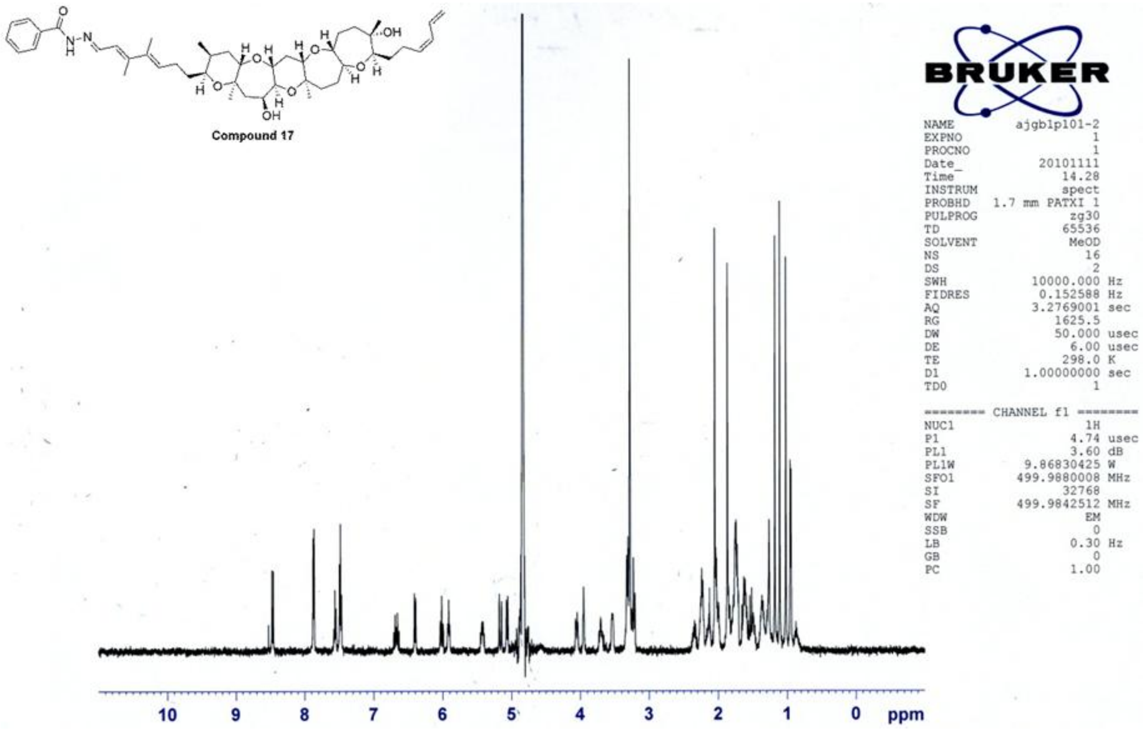

Figure S14. HRMS spectrum of compound **17**.

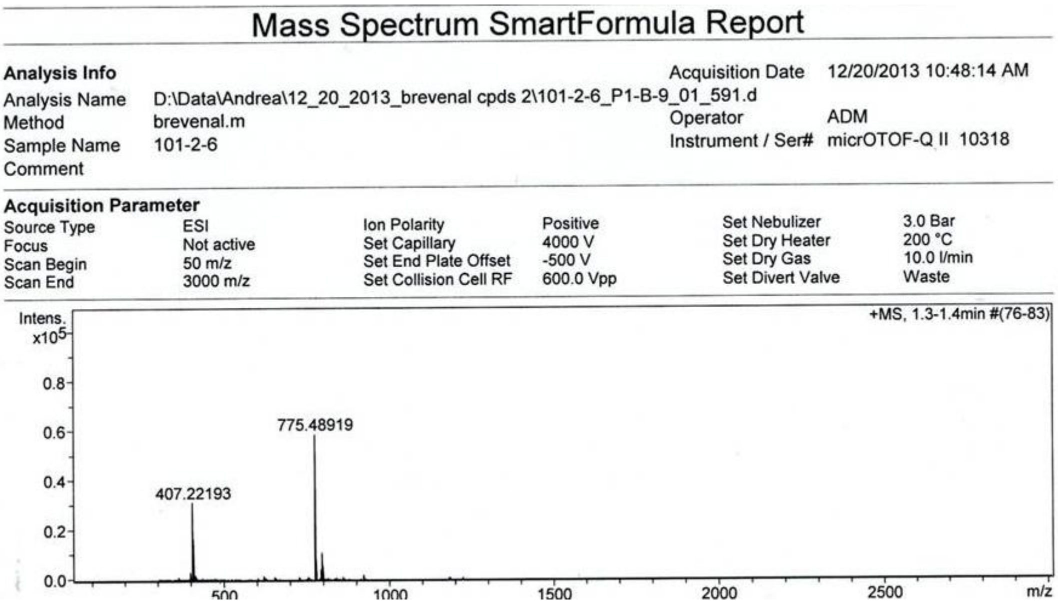

Figure S15. ^{1}H NMR spectrum of compound **18**.

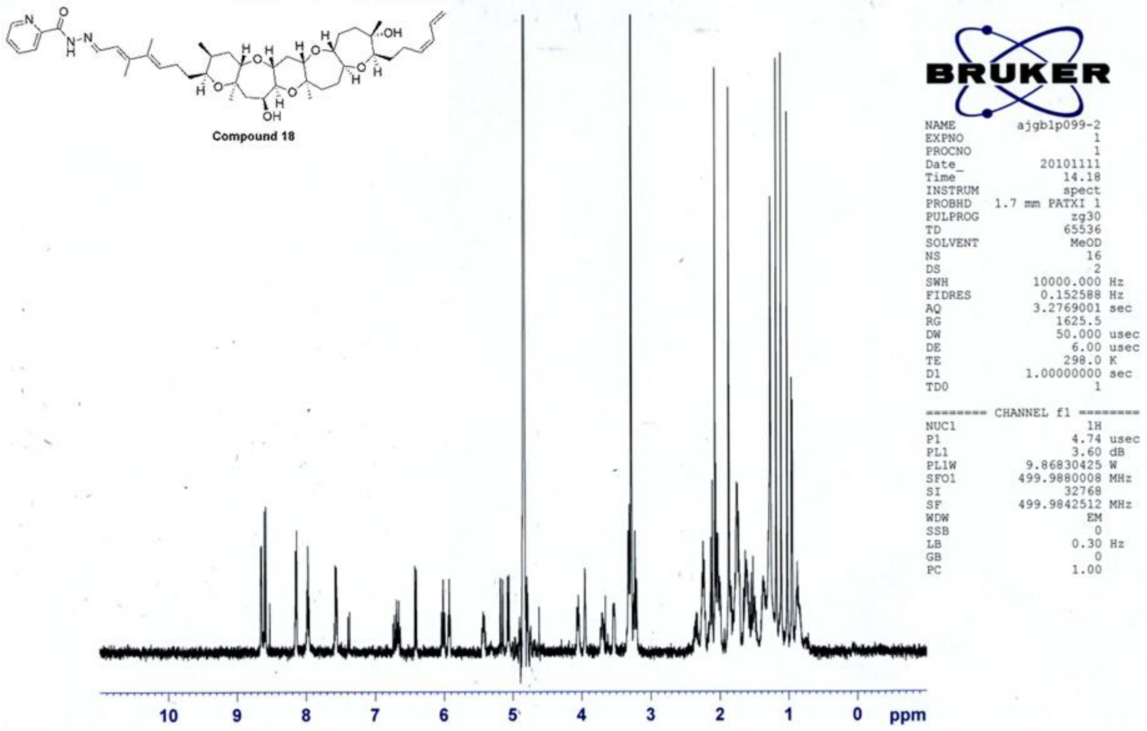

Figure S16. HRMS spectrum of compound **18**.

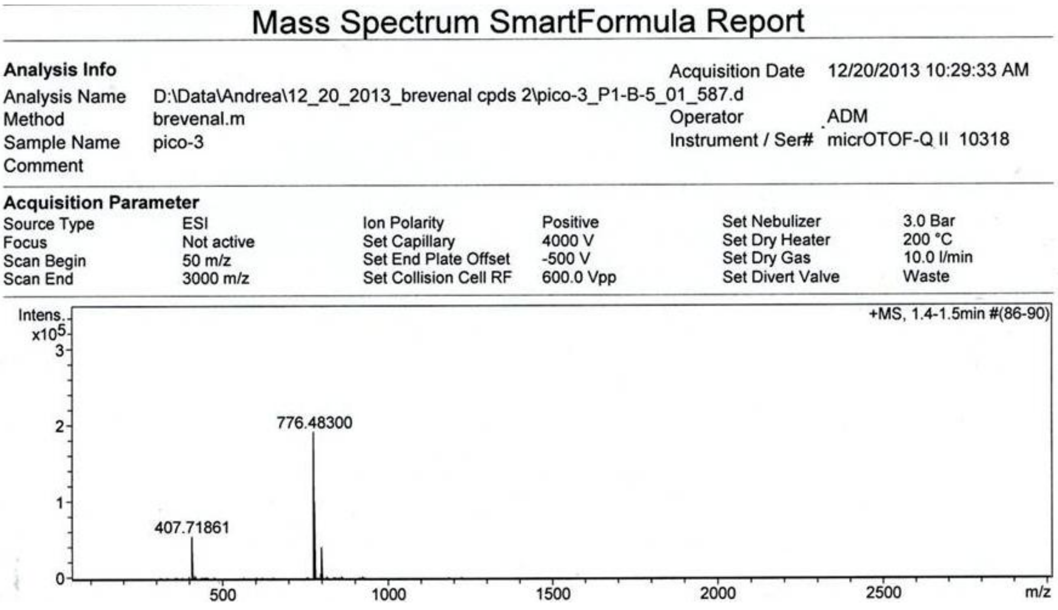

Figure S17. ^{1}H NMR spectrum of compound **19**.

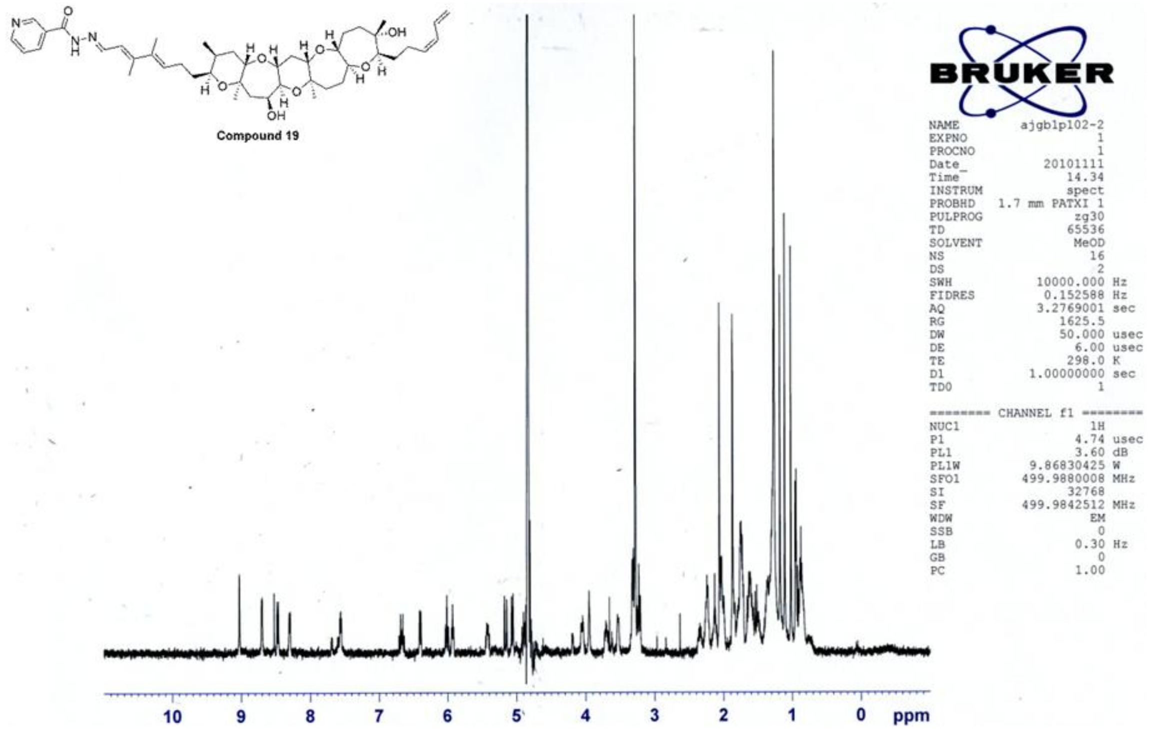

Figure S18. HRMS spectrum of compound **19**.

Mass Spectrum SmartFormula Report

Analysis Info

Analysis Name	D:\Data\Andrea\12_20_2013_brevenal cpds 2\142-2-4_P1-B-1_01_583.d	Acquisition Date	12/20/2013 10:10:51 AM
Method	brevenal.m	Operator	ADM
Sample Name	142-2-4	Instrument / Ser#	micrOTOF-Q II 10318
Comment			

Acquisition Parameter

Source Type	ESI	Ion Polarity	Positive	Set Nebulizer	3.0 Bar
Focus	Not active	Set Capillary	4000 V	Set Dry Heater	200 °C
Scan Begin	50 m/z	Set End Plate Offset	-500 V	Set Dry Gas	10.0 l/min
Scan End	3000 m/z	Set Collision Cell RF	600.0 Vpp	Set Divert Valve	Waste

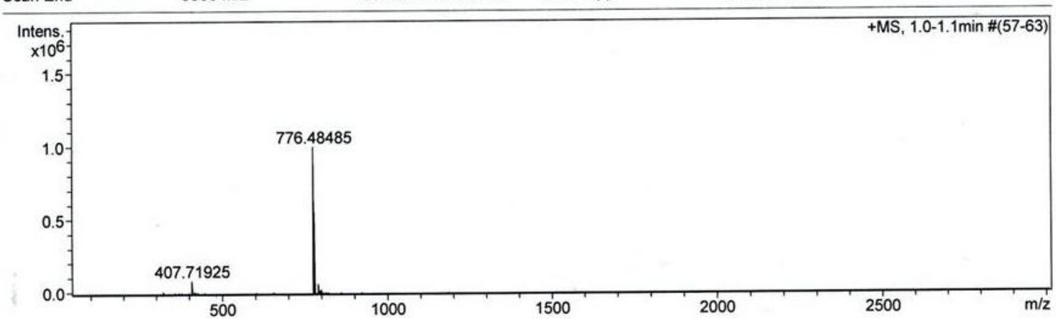

Figure S19. ^{1}H NMR spectrum of compound **20**.

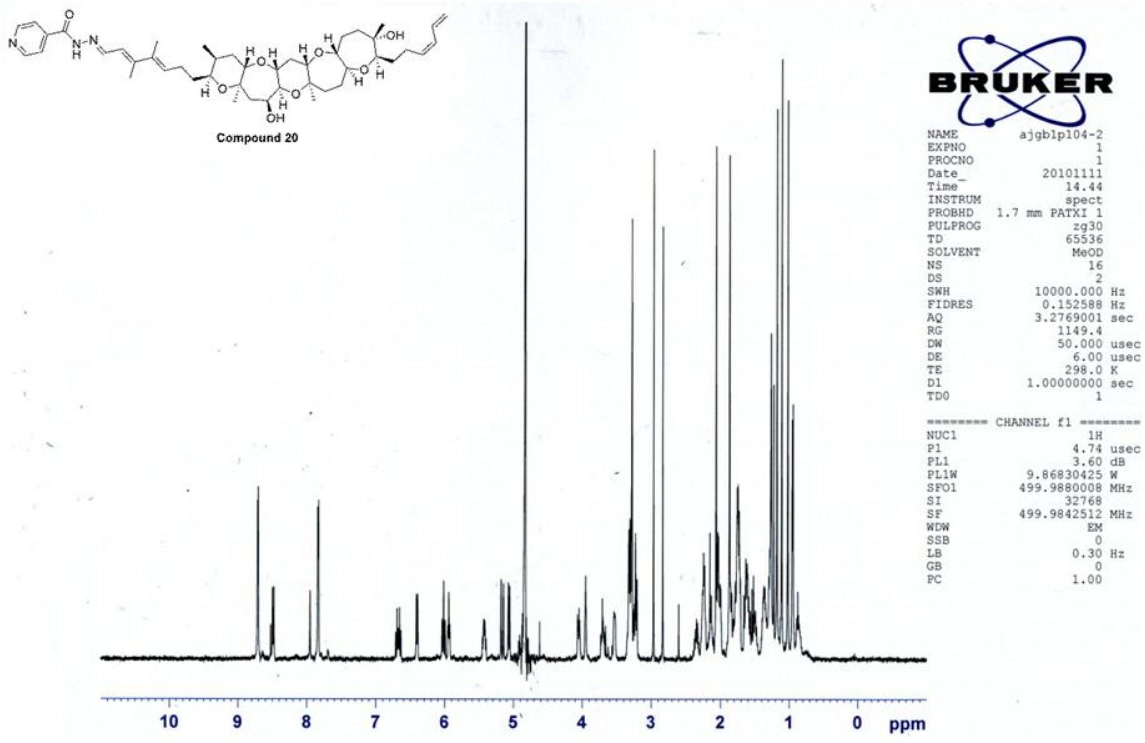

Figure S20. HRMS spectrum of compound **20**.

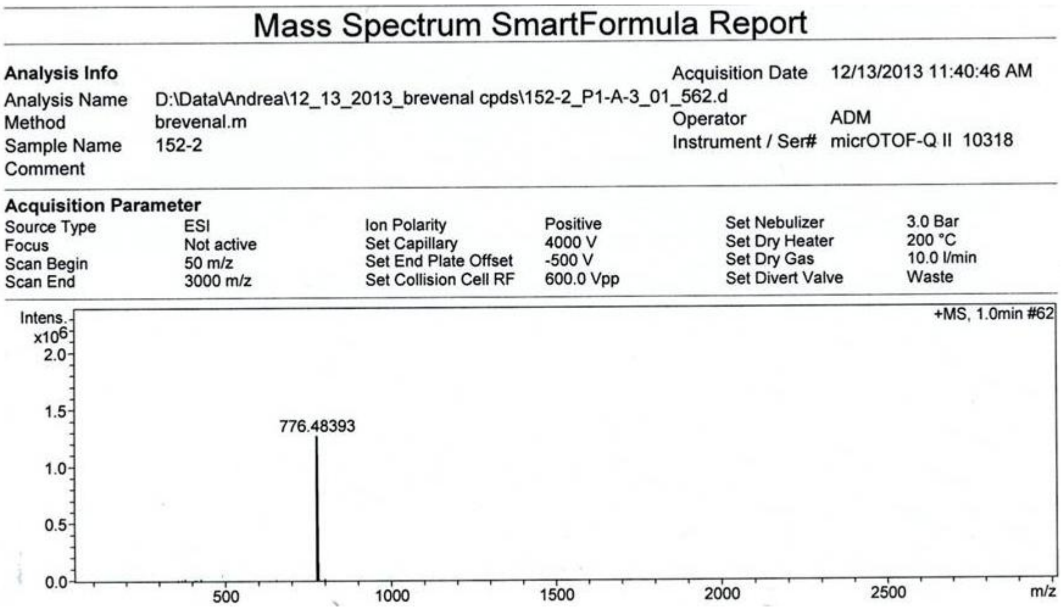

Figure S21. ^{1}H NMR spectrum of compound **21**.

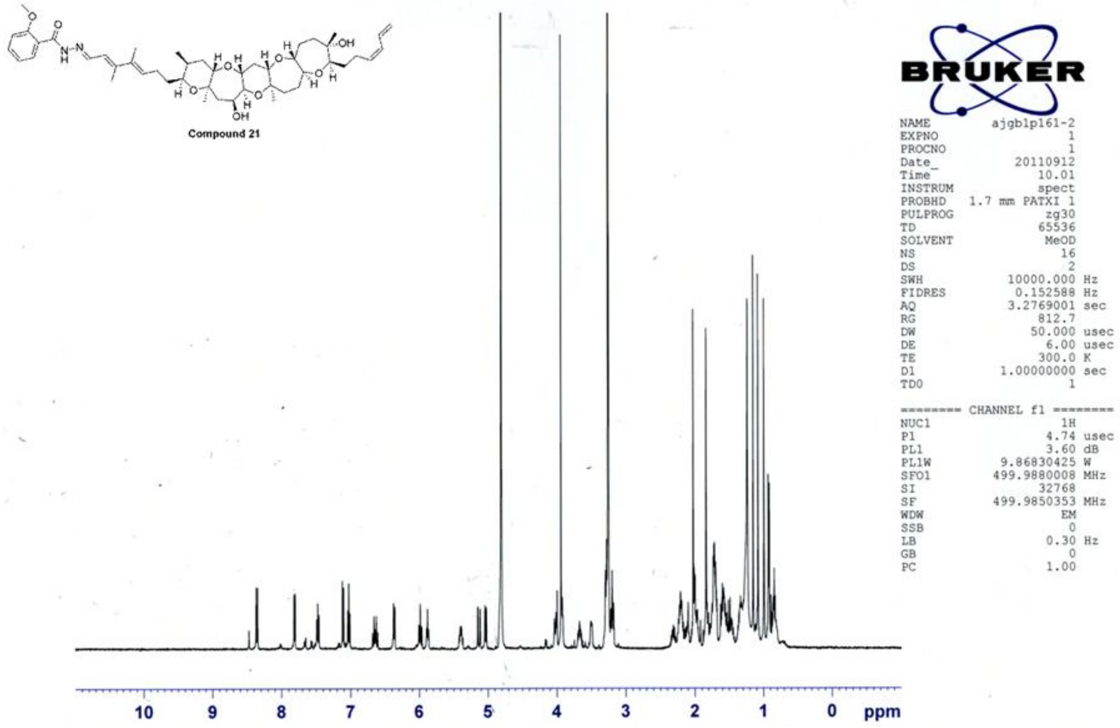

Figure S22. HRMS spectrum of compound **21**.

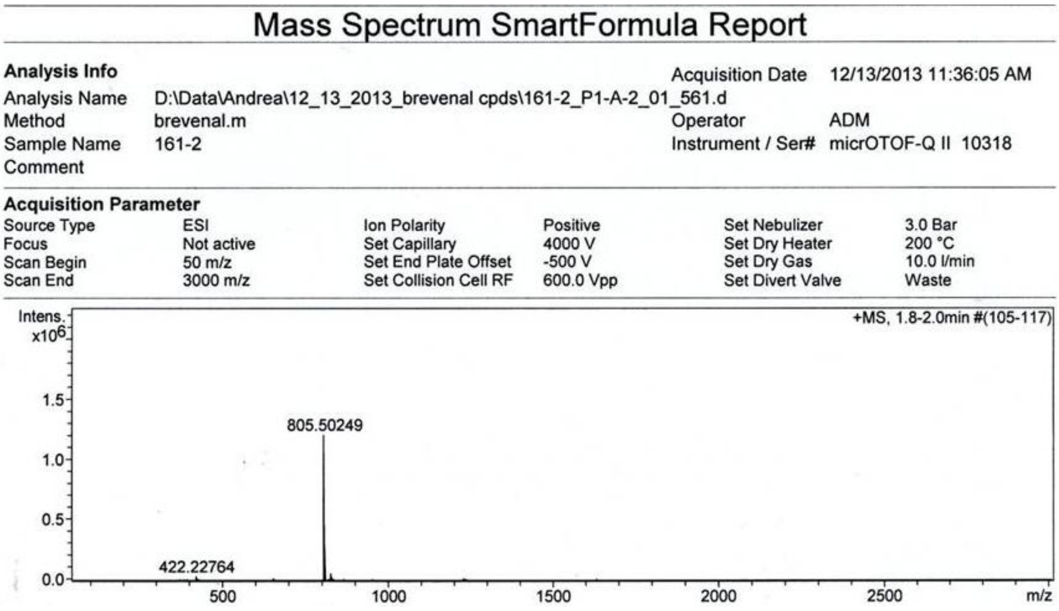

Figure S23. ^{1}H NMR spectrum of compound **22**.

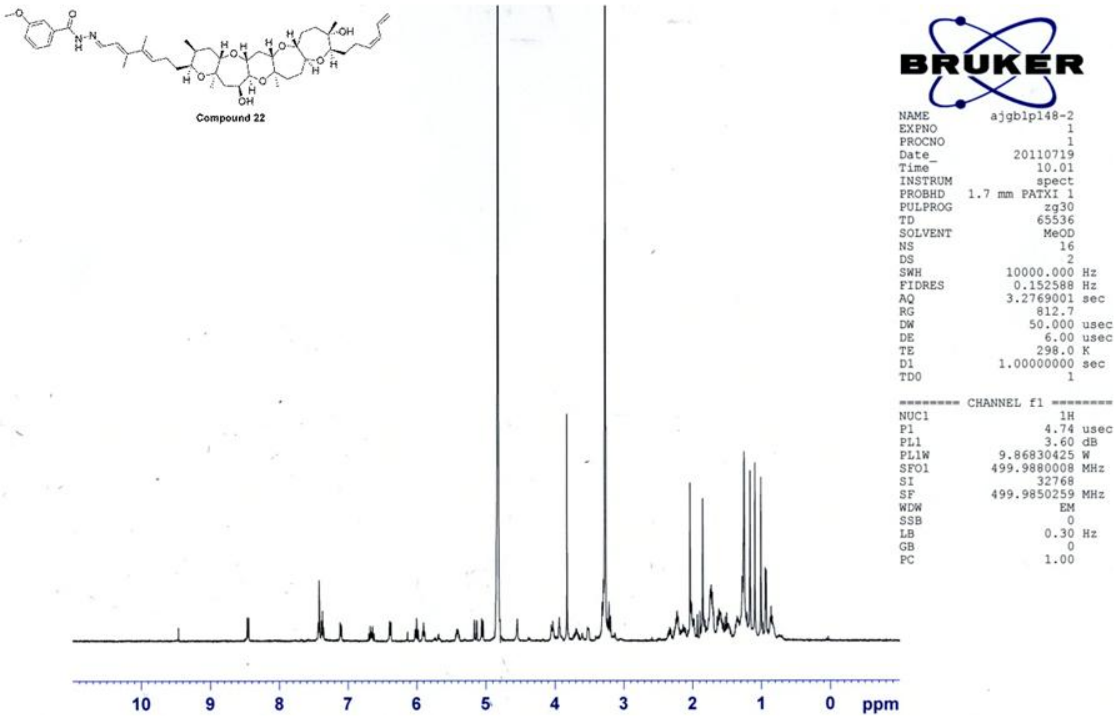

Figure S24. HRMS spectrum of compound **22**.

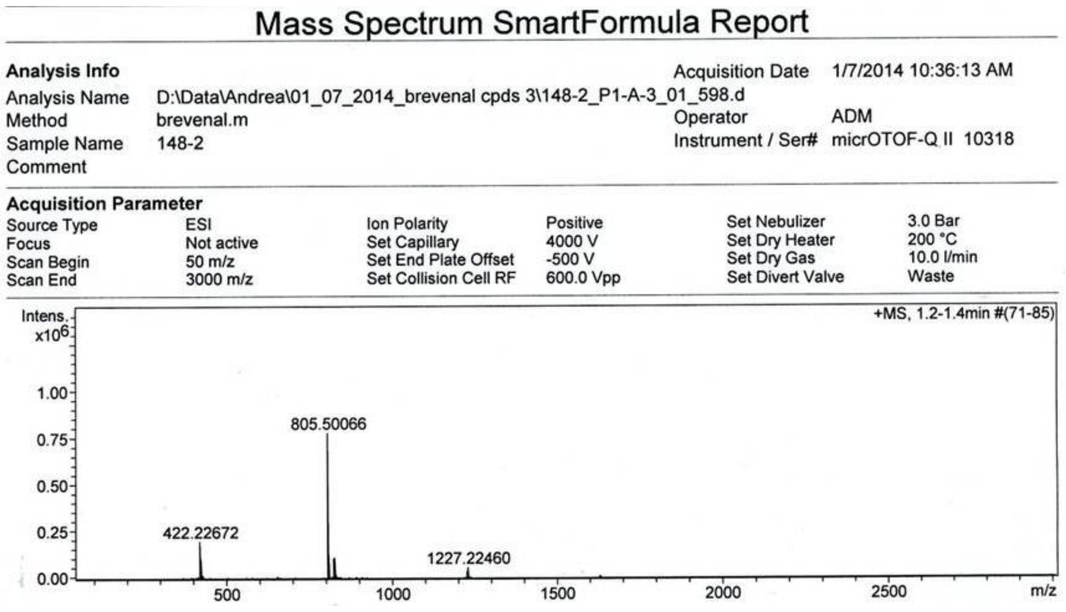

Figure S25. ^{1}H NMR spectrum of compound **23**.

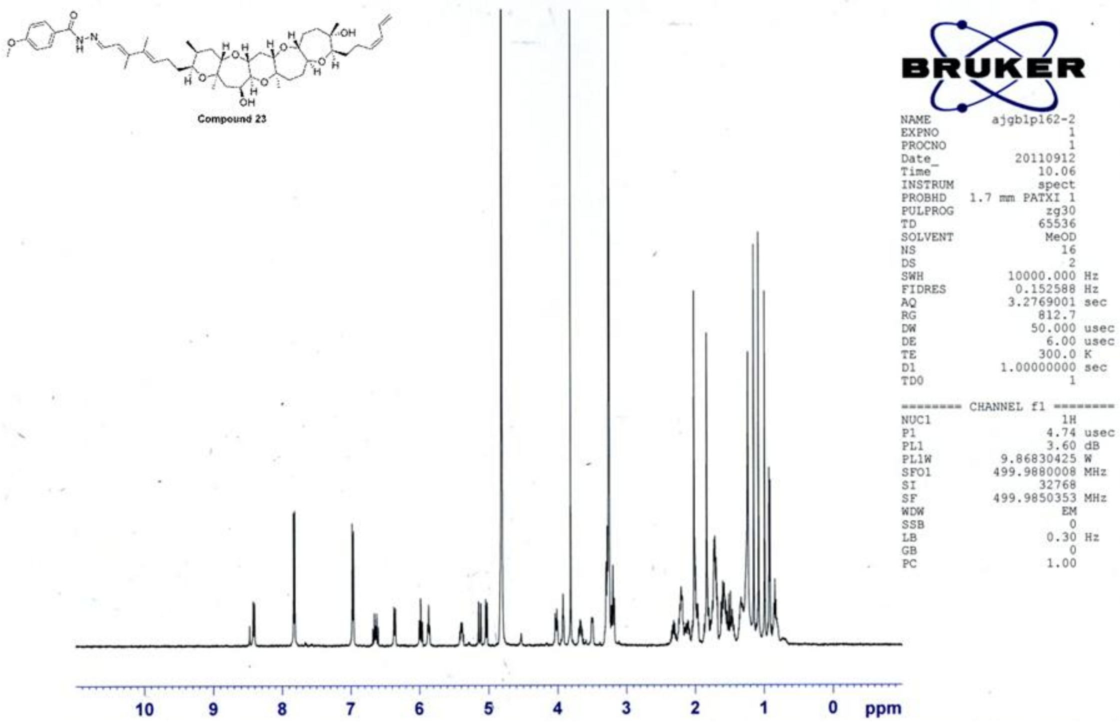

Figure S26. HRMS spectrum of compound **23**.

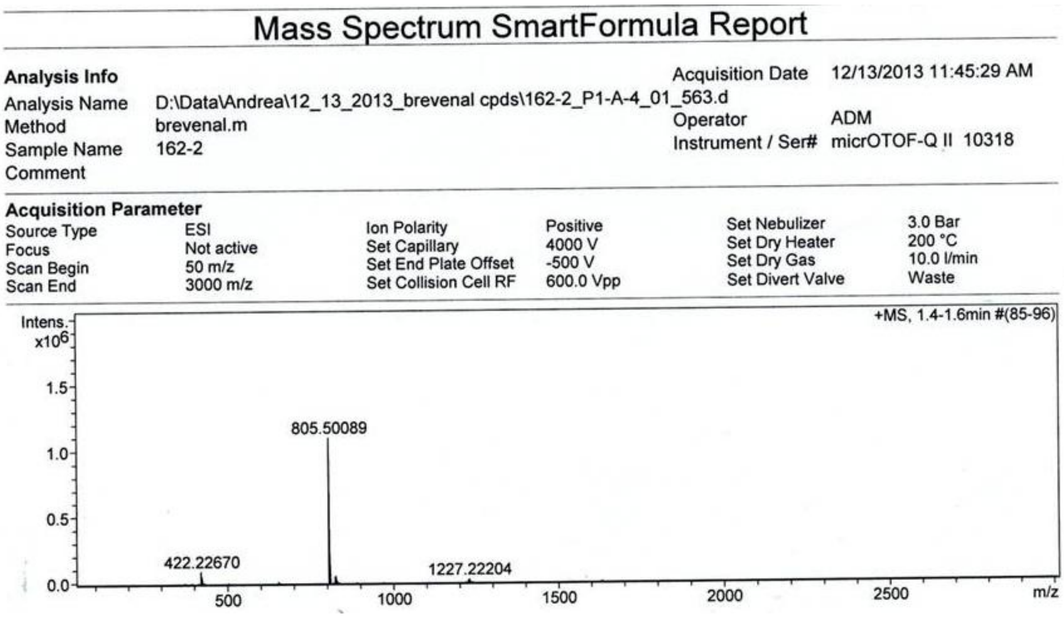
Mass Spectrum SmartFormula Report

**Analysis Info**		Acquisition Date	12/13/2013 11:45:29 AM
Analysis Name	D:\Data\Andrea\12_13_2013_brevenal cpds\162-2_P1-A-4_01_563.d		
Method	brevenal.m	Operator	ADM
Sample Name	162-2	Instrument / Ser#	micrOTOF-Q II 10318
Comment			

Acquisition Parameter

Source Type	ESI	Ion Polarity	Positive	Set Nebulizer	3.0 Bar
Focus	Not active	Set Capillary	4000 V	Set Dry Heater	200 °C
Scan Begin	50 m/z	Set End Plate Offset	-500 V	Set Dry Gas	10.0 l/min
Scan End	3000 m/z	Set Collision Cell RF	600.0 Vpp	Set Divert Valve	Waste

Figure S27. ^{1}H NMR spectrum of compound **24**.

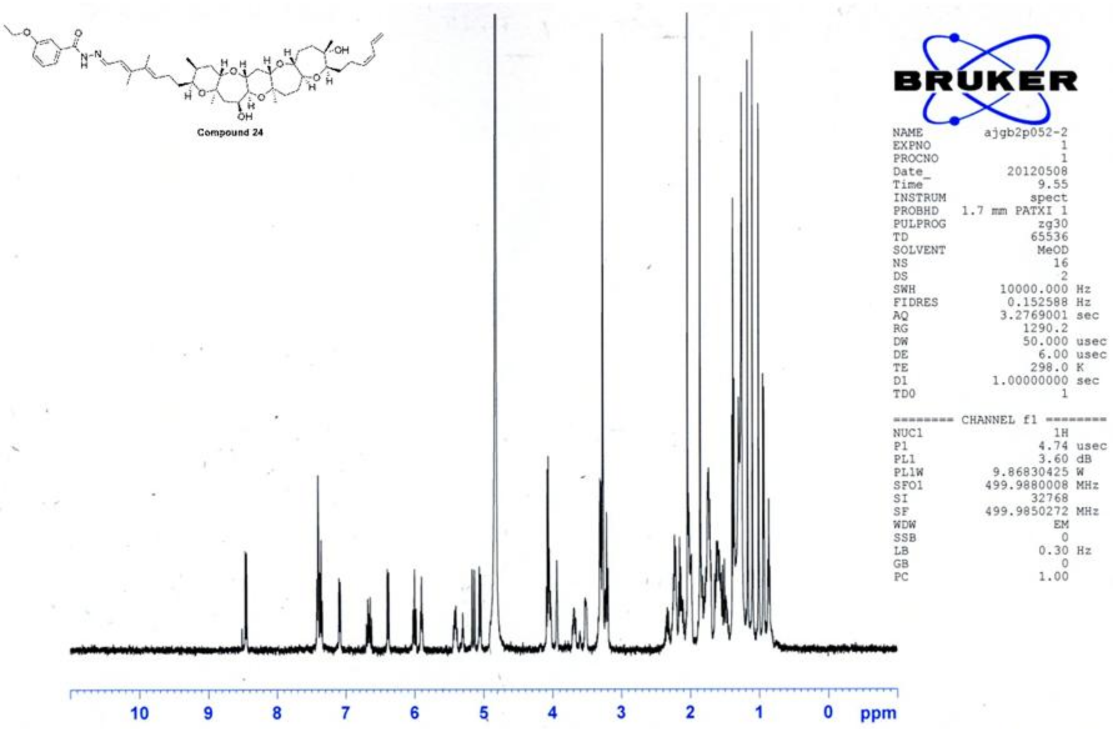

Figure S28. HRMS spectrum of compound **24**.

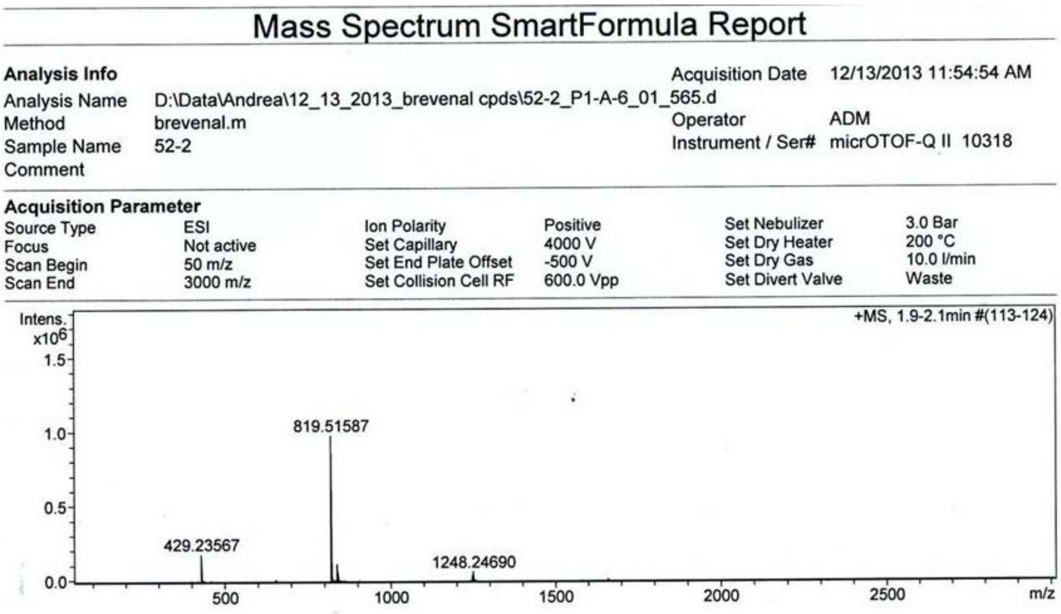
Mass Spectrum SmartFormula Report

Analysis Info

Analysis Name	D:\Data\Andrea\12_13_2013_brevenal cpds\52-2_P1-A-6_01_565.d	Acquisition Date	12/13/2013 11:54:54 AM
Method	brevenal.m	Operator	ADM
Sample Name	52-2	Instrument / Ser#	micrOTOF-Q II 10318
Comment			

Acquisition Parameter

Source Type	ESI	Ion Polarity	Positive	Set Nebulizer	3.0 Bar
Focus	Not active	Set Capillary	4000 V	Set Dry Heater	200 °C
Scan Begin	50 m/z	Set End Plate Offset	-500 V	Set Dry Gas	10.0 l/min
Scan End	3000 m/z	Set Collision Cell RF	600.0 Vpp	Set Divert Valve	Waste

Figure S29. ^{1}H NMR spectrum of compound **25**.

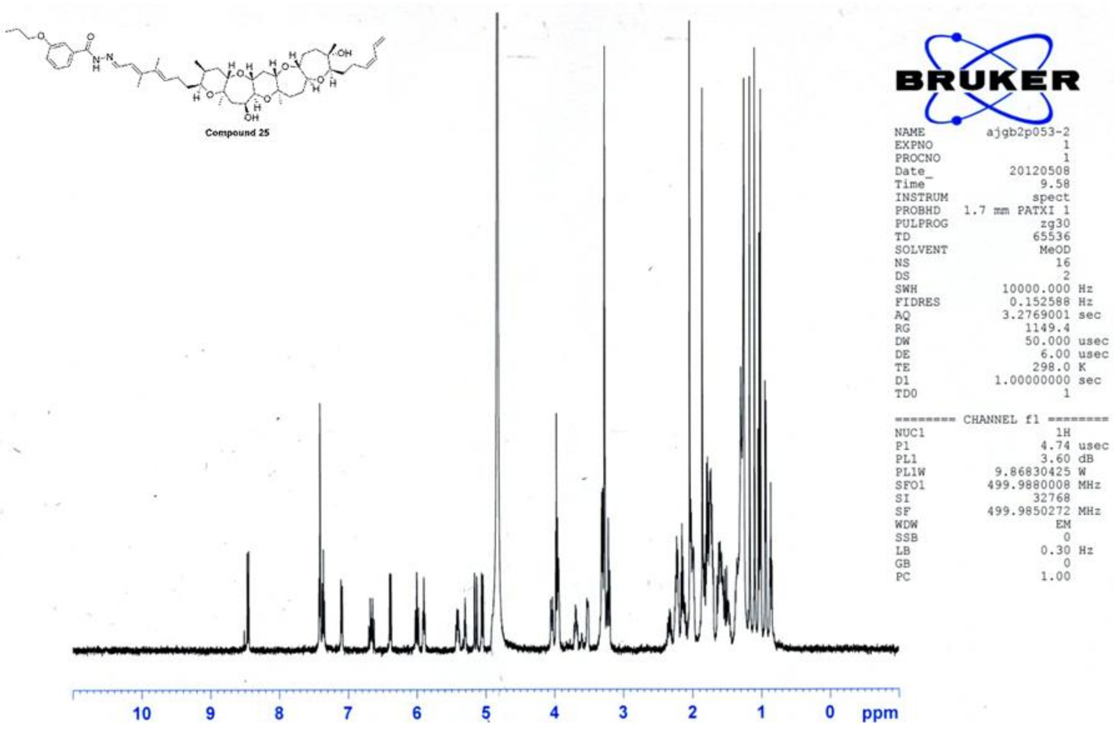

Figure S30. HRMS spectrum of compound **25**.

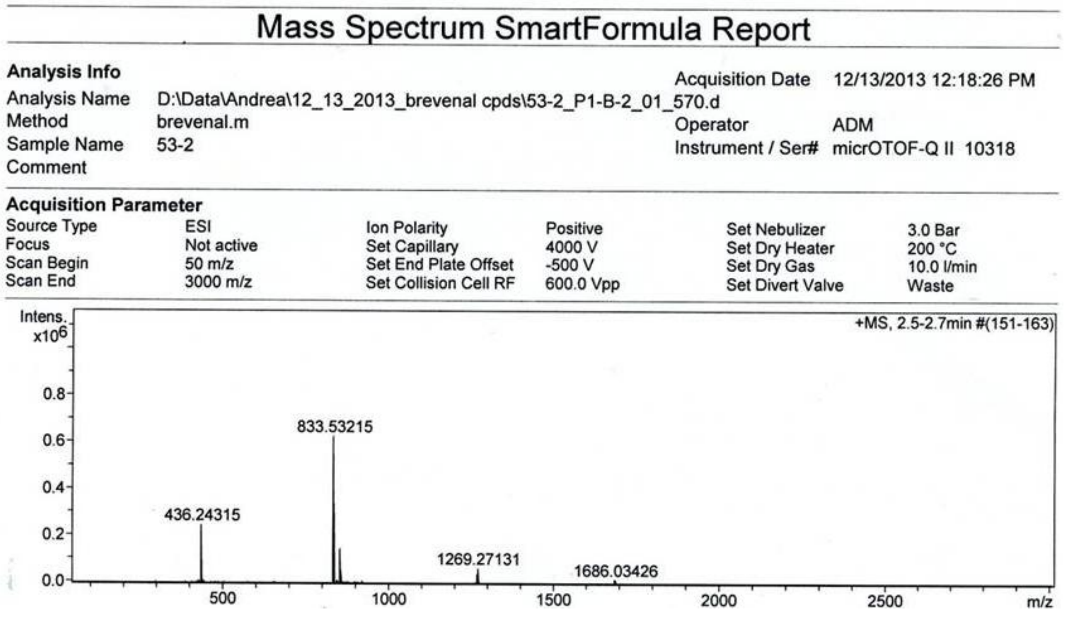

Figure S31. ^{1}H NMR spectrum of compound **26**.

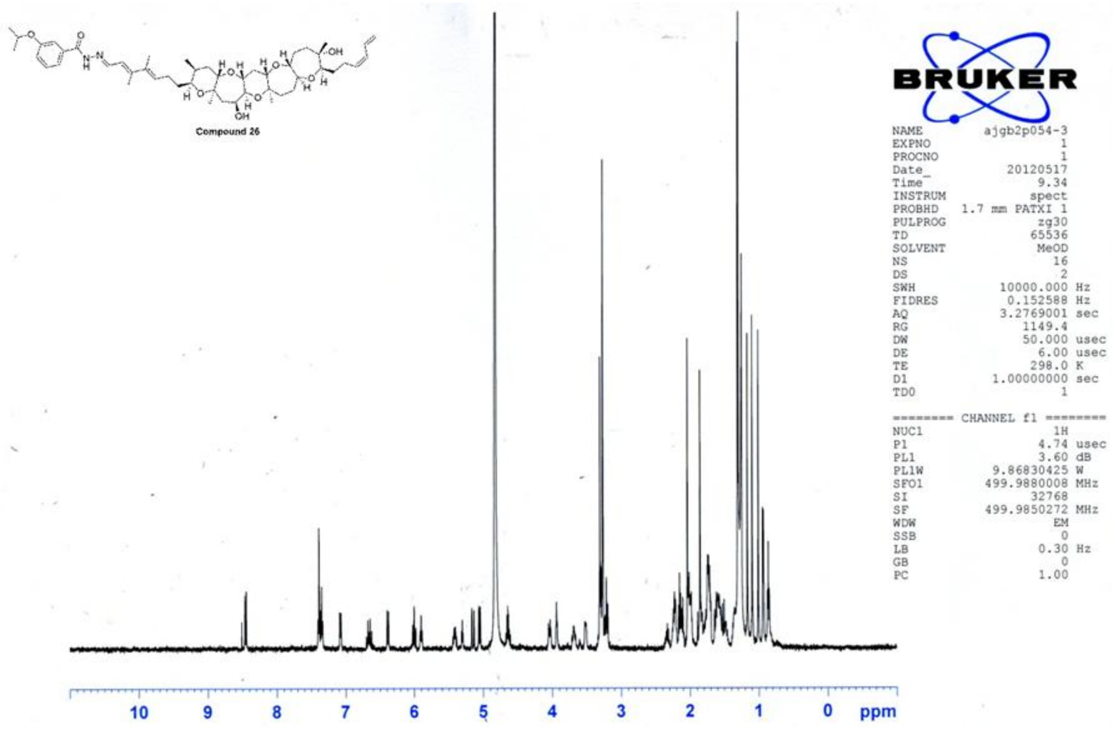

Figure S32. HRMS spectrum of compound **26**.

Mass Spectrum SmartFormula Report

Analysis Info

Analysis Name	D:\Data\Andrea\12_20_2013_brevenal cpds 2\54-2-3_P1-C-3_01_594.d	Acquisition Date	12/20/2013 11:02:16 AM
Method	brevenal.m	Operator	ADM
Sample Name	54-2-3	Instrument / Ser#	micrOTOF-Q II 10318
Comment			

Acquisition Parameter

Source Type	ESI	Ion Polarity	Positive	Set Nebulizer	3.0 Bar
Focus	Not active	Set Capillary	4000 V	Set Dry Heater	200 °C
Scan Begin	50 m/z	Set End Plate Offset	-500 V	Set Dry Gas	10.0 l/min
Scan End	3000 m/z	Set Collision Cell RF	600.0 Vpp	Set Divert Valve	Waste

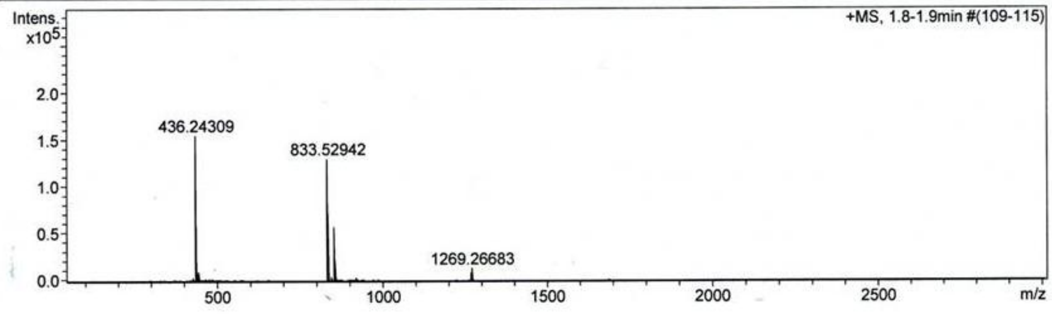

Figure S33. ^{1}H NMR spectrum of compound **27**.

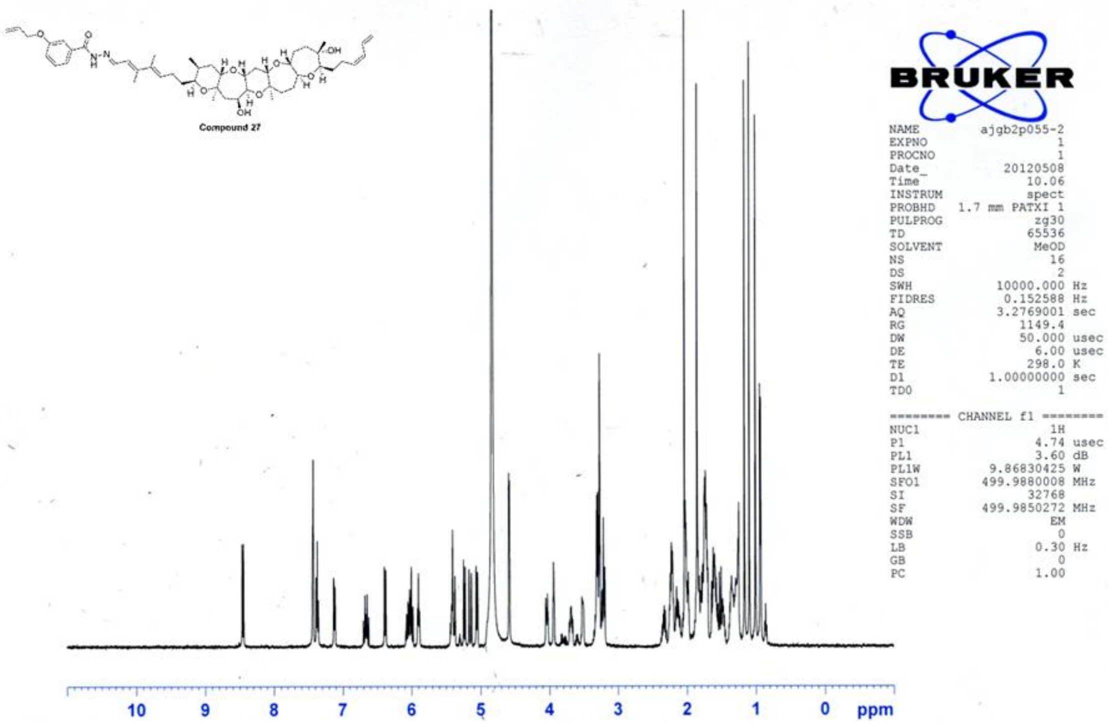

Figure S34. HRMS spectrum of compound **27**.

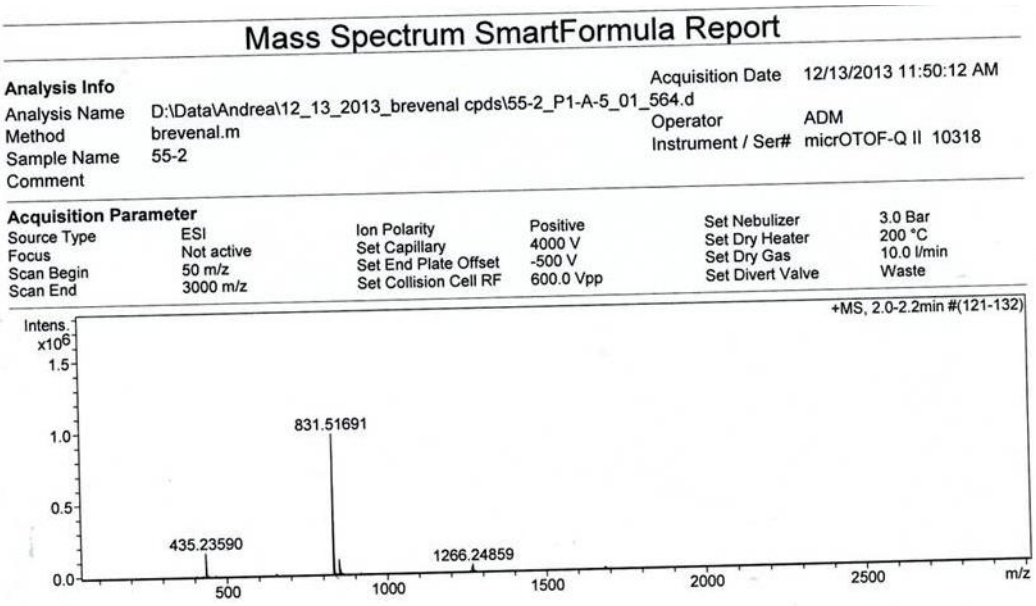

Figure S35. ^{1}H NMR spectrum of compound **28**.

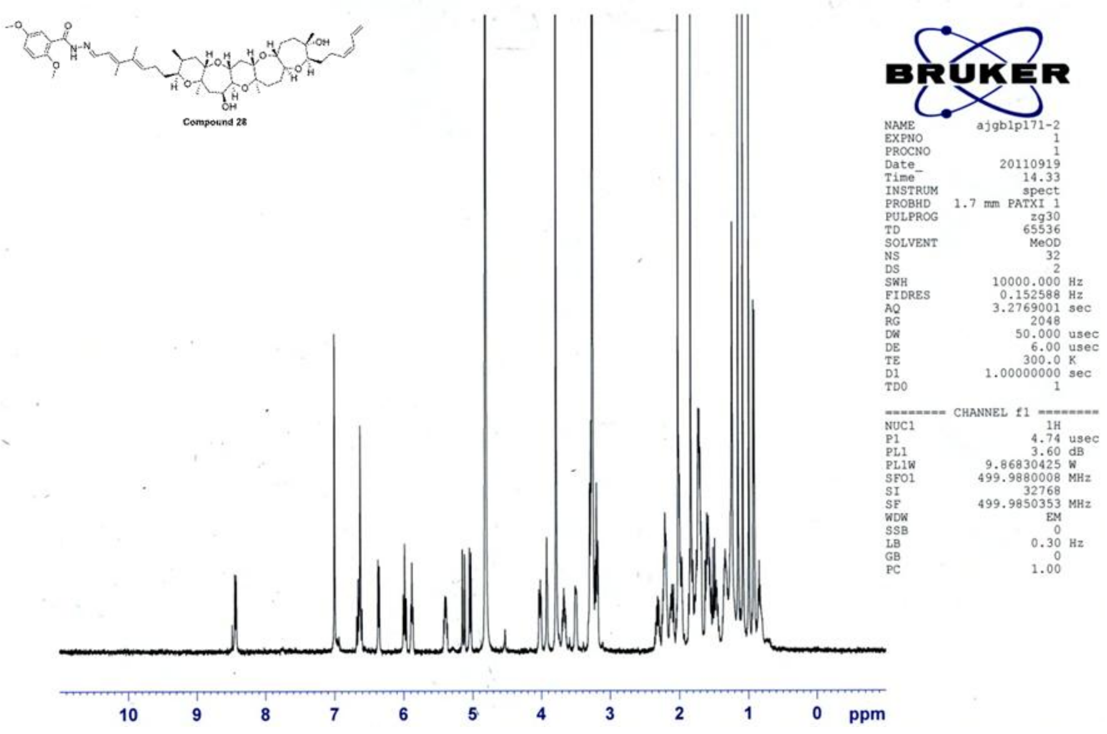

Figure S36. HRMS spectrum of compound **28**.

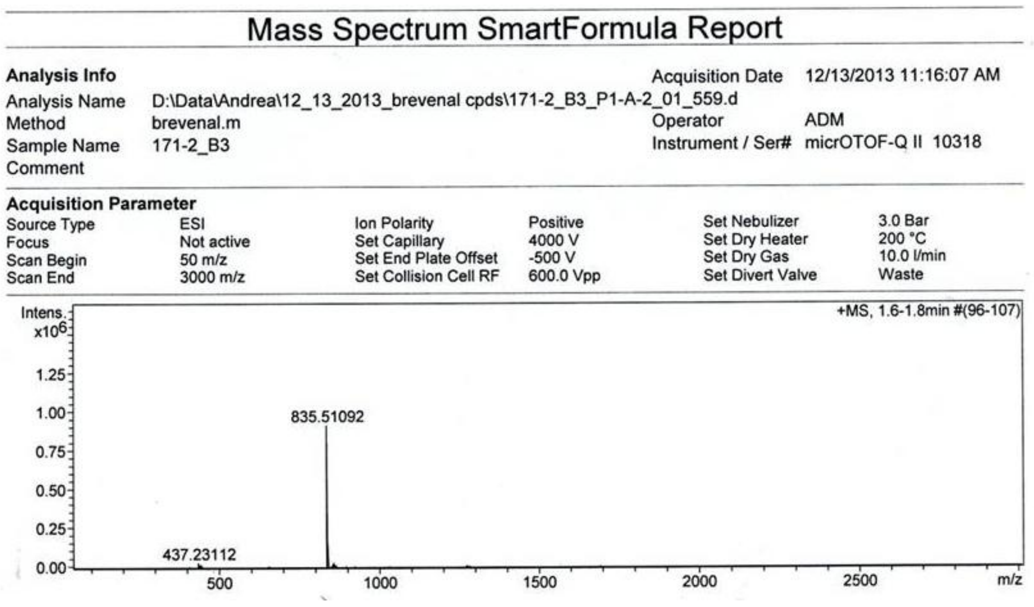

Figure S37. ^{1}H NMR spectrum of compound **29**.

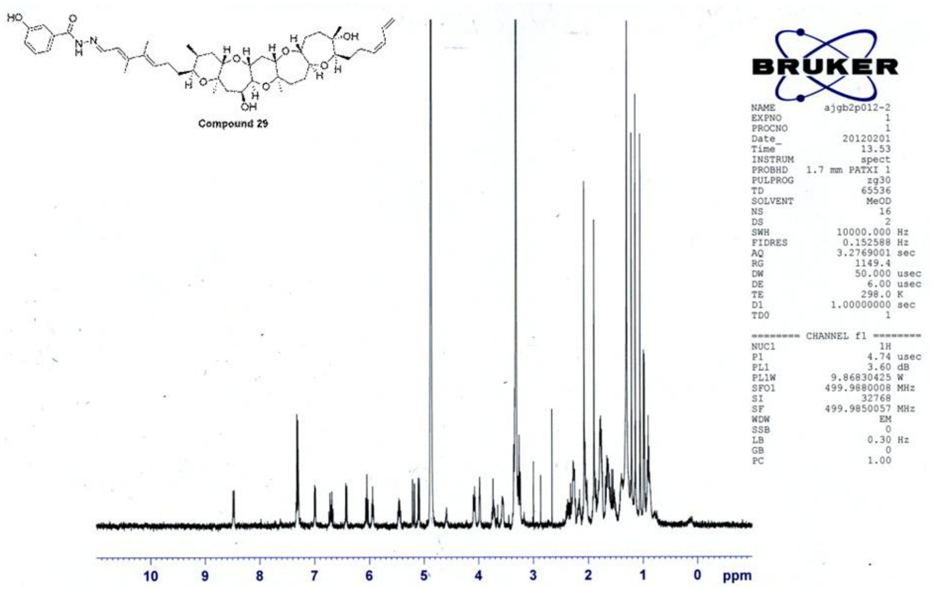

Figure S38. HRMS spectrum of compound **29**.

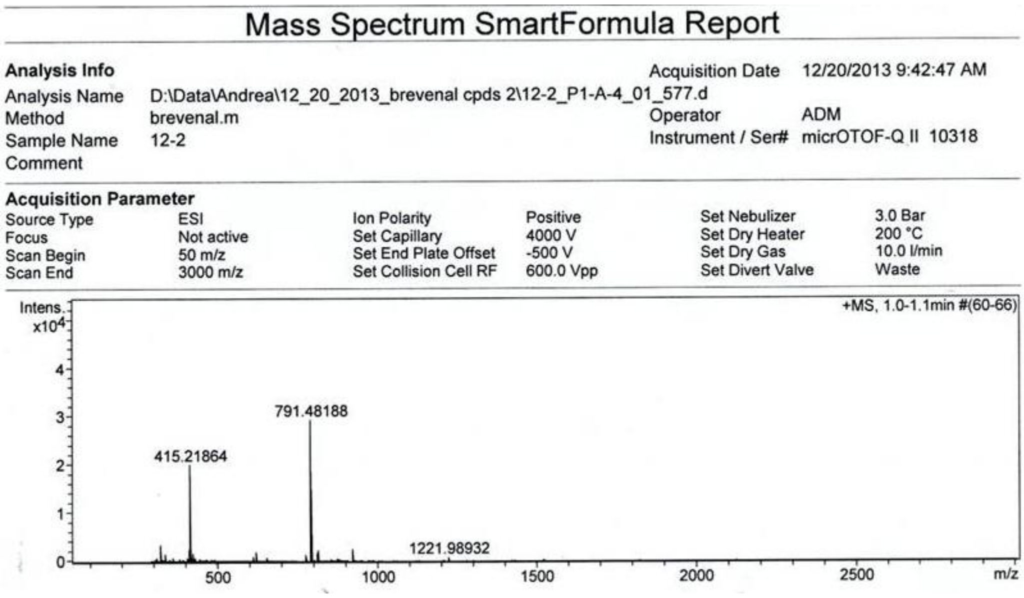

Figure S39. ^{1}H NMR spectrum of compound **30**.

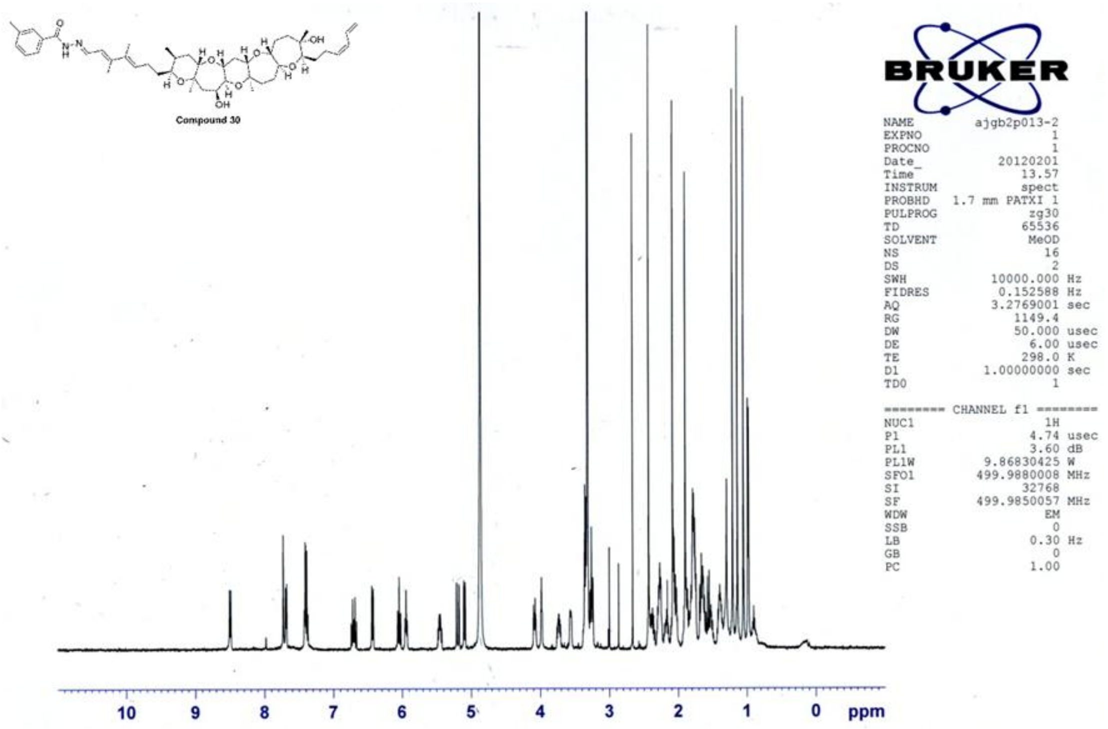

Figure S40. HRMS spectrum of compound **30**.

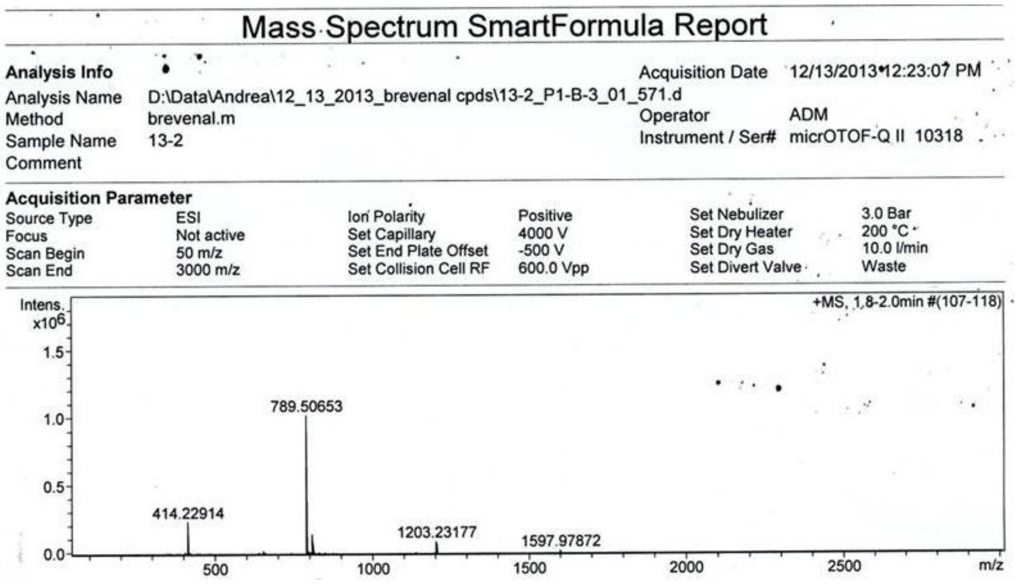

Figure S41. ^{1}H NMR spectrum of compound **31**.

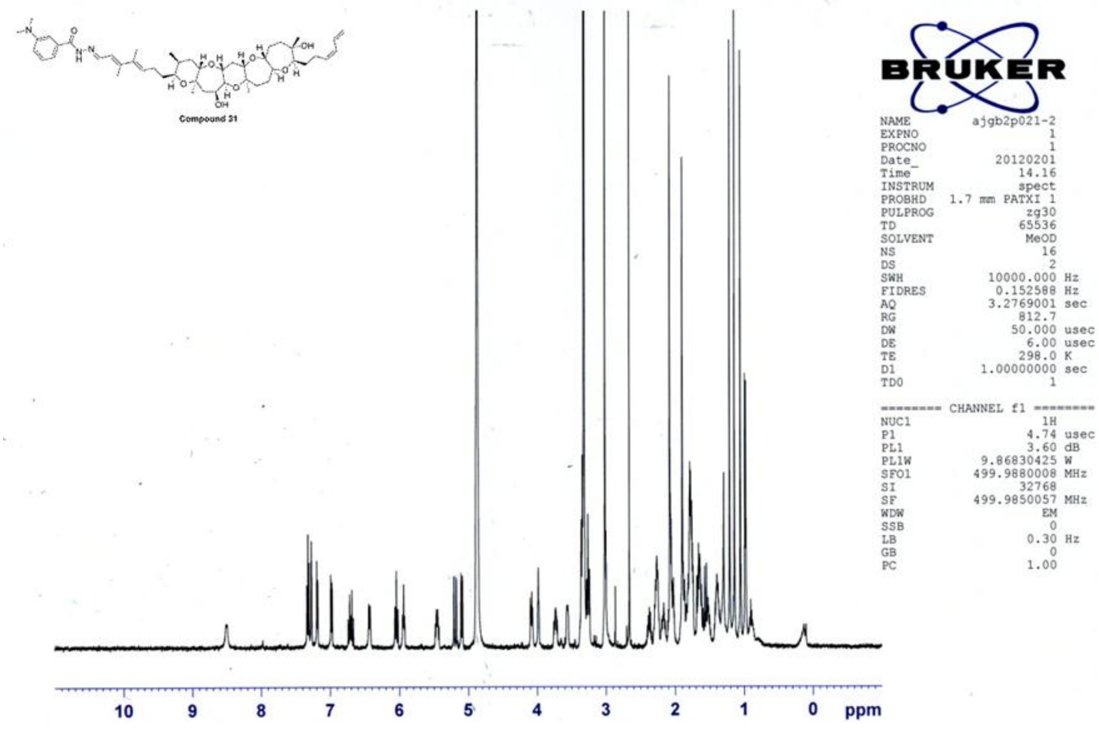

Figure S42. HRMS spectrum of compound **31**.

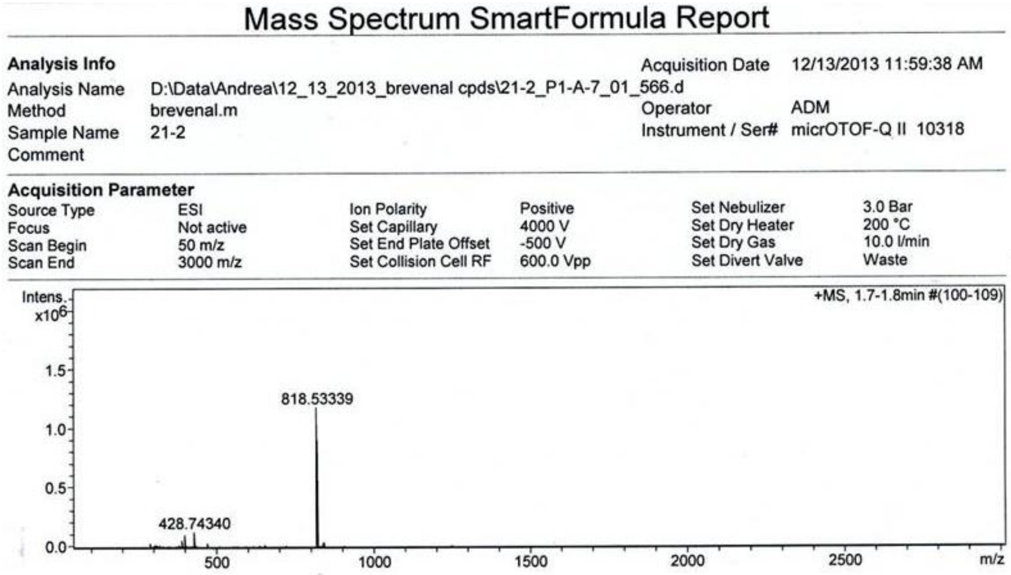

Figure S43. ^{1}H NMR spectrum of compound **32**.

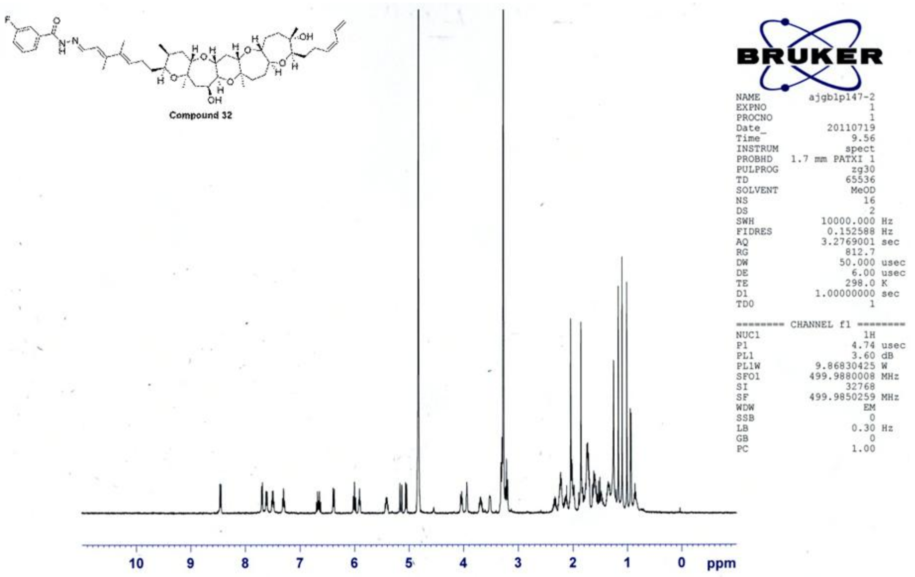

Figure S44. HRMS spectrum of compound **32**.

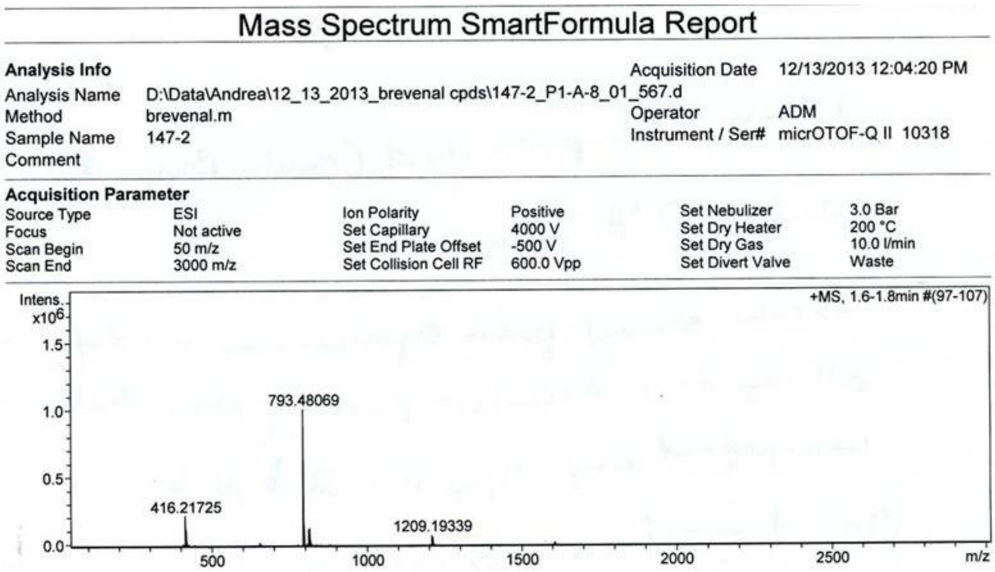

Figure S45. ^{1}H NMR spectrum of compound **33**.

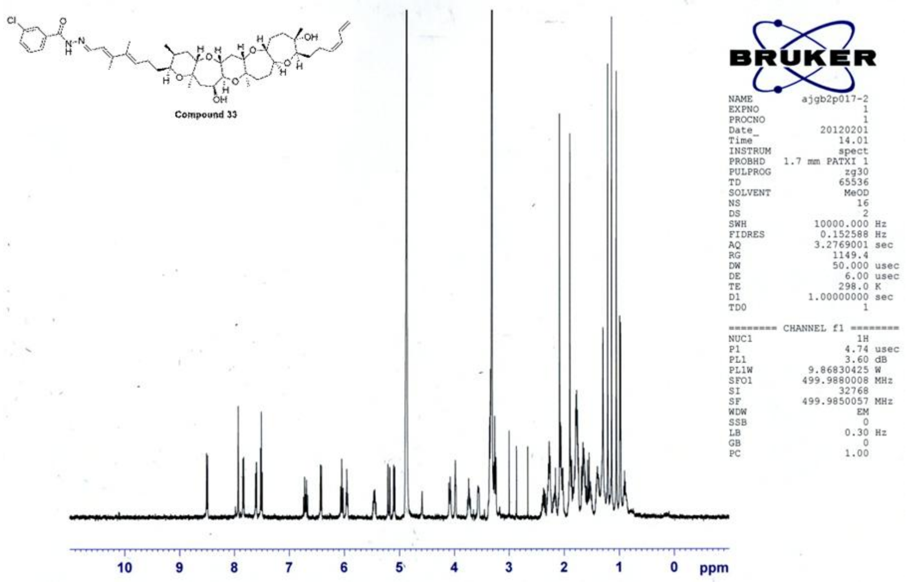

Figure S46. HRMS spectrum of compound **33**.

Mass Spectrum SmartFormula Report

Analysis Info

Analysis Name	D:\Data\Andrea\12_20_2013_brevenal cpds 2\P124-2_P1-B-6_01_588.d	Acquisition Date	12/20/2013 10:34:14 AM
Method	brevenal.m	Operator	ADM
Sample Name	P124-2	Instrument / Ser#	micrOTOF-Q II 10318
Comment			

Acquisition Parameter

Source Type	ESI	Ion Polarity	Positive	Set Nebulizer	3.0 Bar
Focus	Not active	Set Capillary	4000 V	Set Dry Heater	200 °C
Scan Begin	50 m/z	Set End Plate Offset	-500 V	Set Dry Gas	10.0 l/min
Scan End	3000 m/z	Set Collision Cell RF	600.0 Vpp	Set Divert Valve	Waste

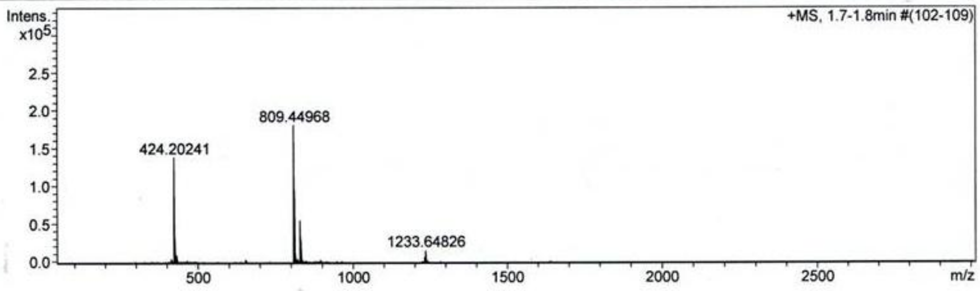

Figure S47. ^{1}H NMR spectrum of compound **34**.

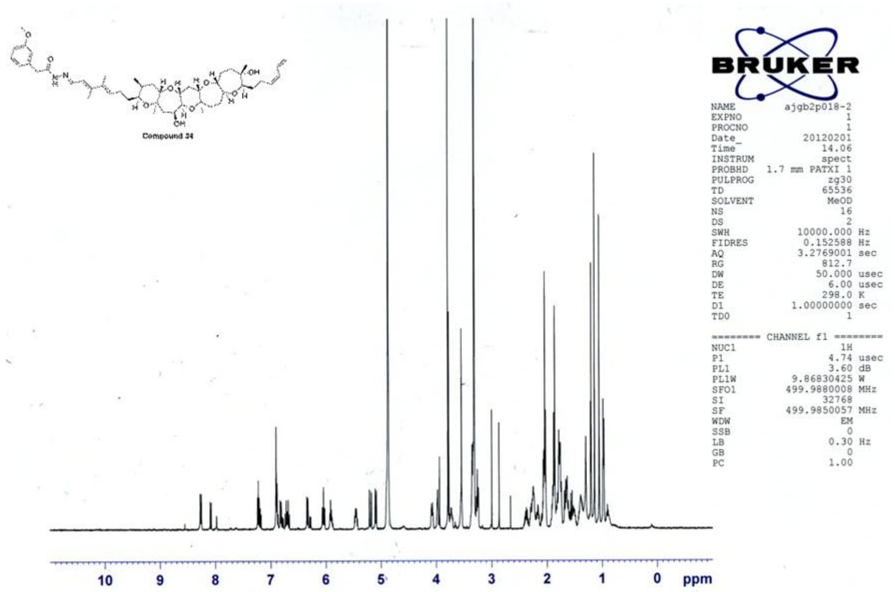

Figure S48. HRMS spectrum of compound **34**.

Mass Spectrum SmartFormula Report

Analysis Info			
Analysis Name	D:\Data\Andrea\12_20_2013_brevenal cpds 2\18-2-5-2_P1-A-9_01_582.d	Acquisition Date	12/20/2013 10:06:12 AM
Method	brevenal.m	Operator	ADM
Sample Name	18-2-5-2	Instrument / Ser#	micrOTOF-Q II 10318
Comment			

Acquisition Parameter

Source Type	ESI	Ion Polarity	Positive	Set Nebulizer	3.0 Bar
Focus	Not active	Set Capillary	4000 V	Set Dry Heater	200 °C
Scan Begin	50 m/z	Set End Plate Offset	-500 V	Set Dry Gas	10.0 l/min
Scan End	3000 m/z	Set Collision Cell RF	600.0 Vpp	Set Divert Valve	Waste

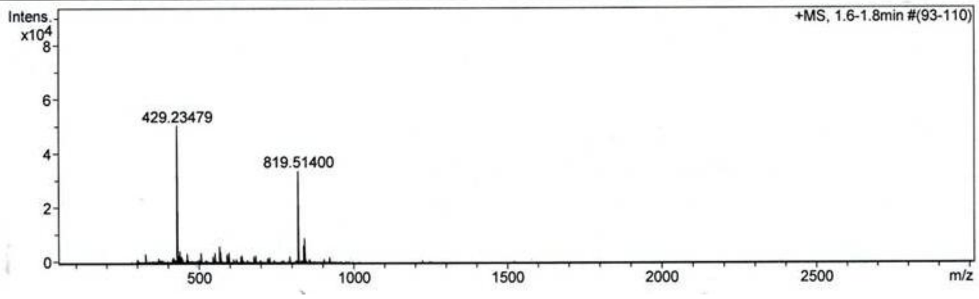

Figure S49. ^{1}H NMR spectrum of compound **35**.

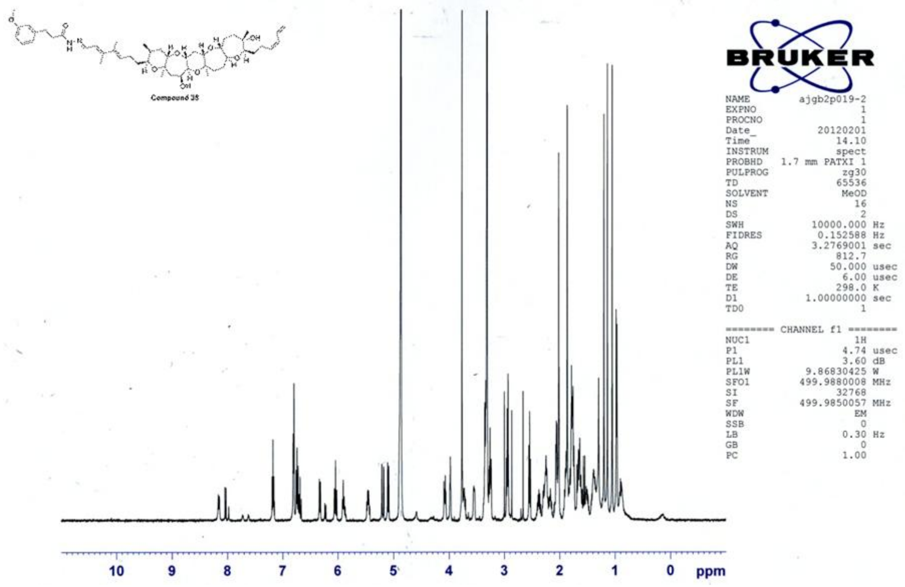

Figure S50. HRMS spectrum of compound **35**.

Mass Spectrum SmartFormula Report

Analysis Info

Analysis Name	D:\Data\Andrea\12_20_2013_brevenal cpds 2\19-2-4-1_P1-B-3_01_585.d	Acquisition Date	12/20/2013 10:20:13 AM
Method	brevenal.m	Operator	ADM
Sample Name	19-2-4-1	Instrument / Ser#	micrOTOF-Q II 10318
Comment			

Acquisition Parameter

Source Type	ESI	Ion Polarity	Positive	Set Nebulizer	3.0 Bar
Focus	Not active	Set Capillary	4000 V	Set Dry Heater	200 °C
Scan Begin	50 m/z	Set End Plate Offset	-500 V	Set Dry Gas	10.0 l/min
Scan End	3000 m/z	Set Collision Cell RF	600.0 Vpp	Set Divert Valve	Waste

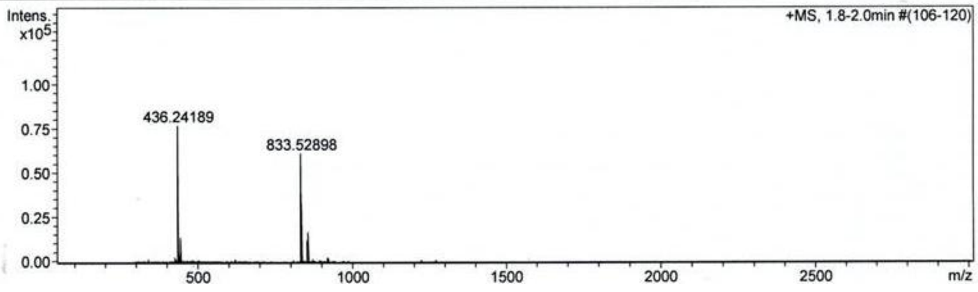

Figure S51. ^{1}H NMR spectrum of compound **37**.

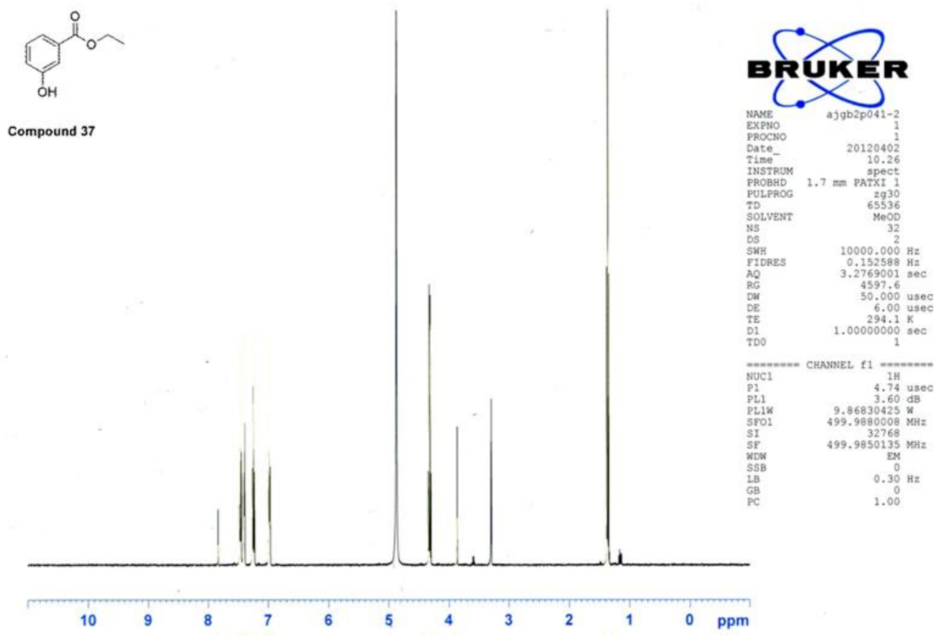

Figure S52. ^{1}H NMR spectrum of compound **38a**.

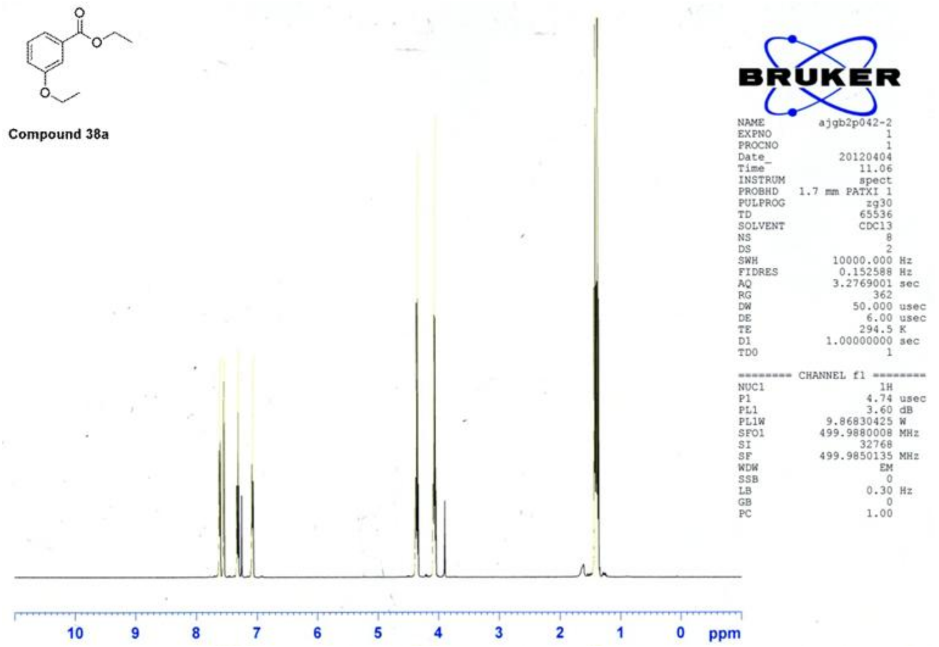

Figure S53. ^{1}H NMR spectrum of compound **38b**.

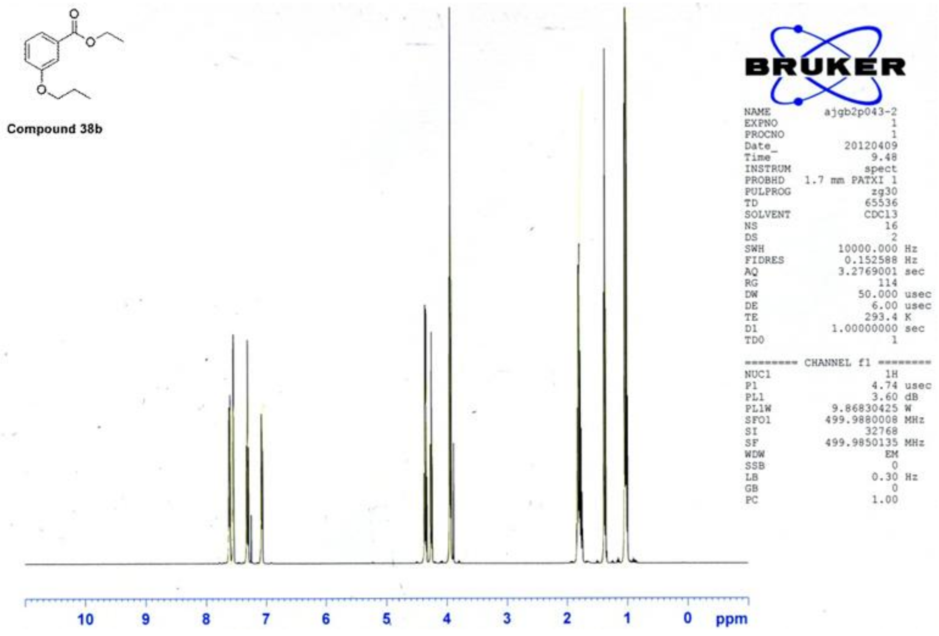

Figure S54. ^{1}H NMR spectrum of compound **38c**.

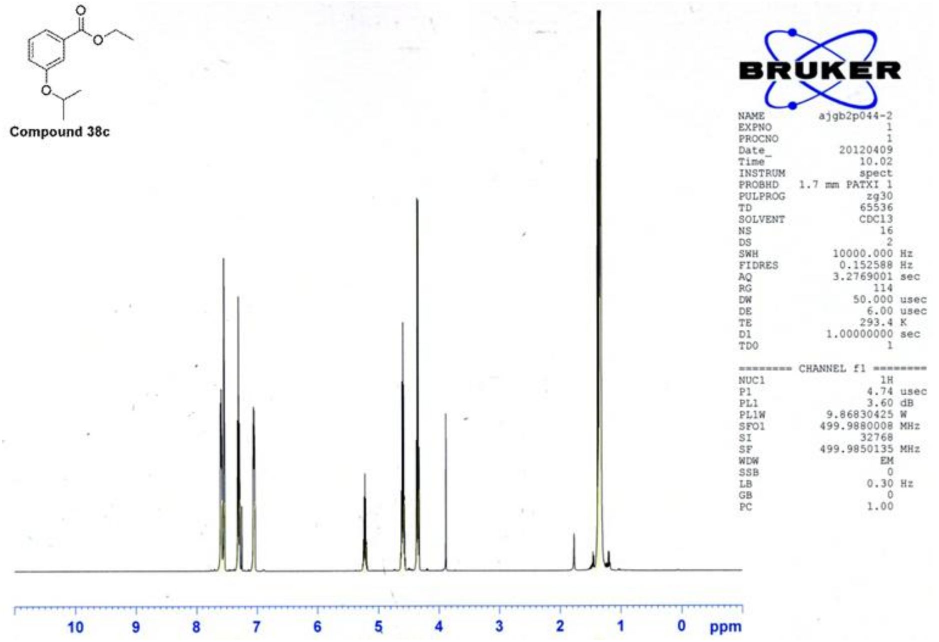

Figure S55. ^{1}H NMR spectrum of compound **38d**.

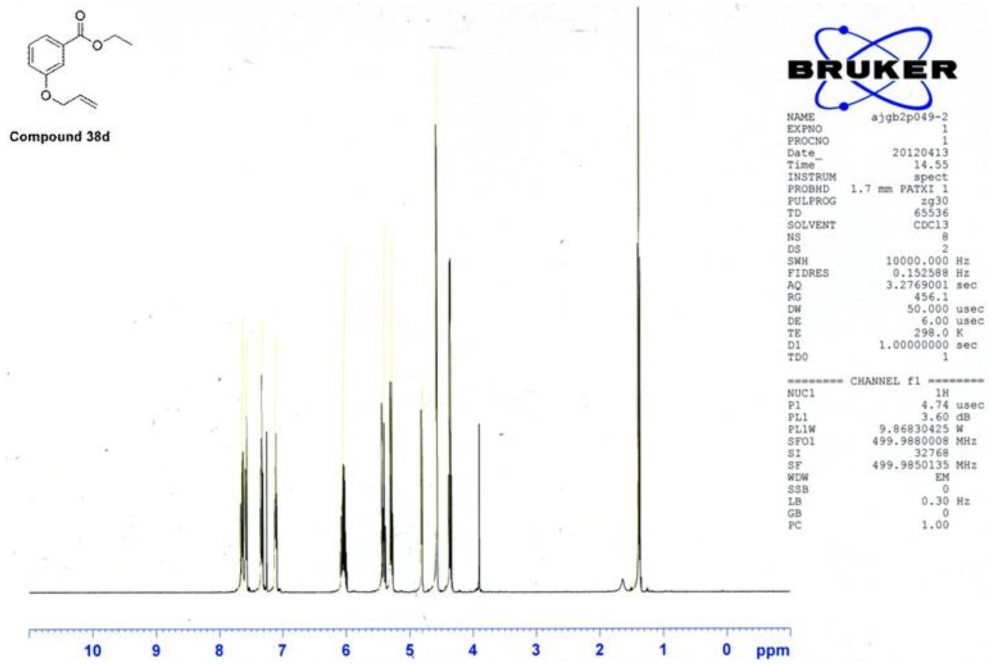

Figure S56. ^{1}H NMR spectrum of compound **39a**.

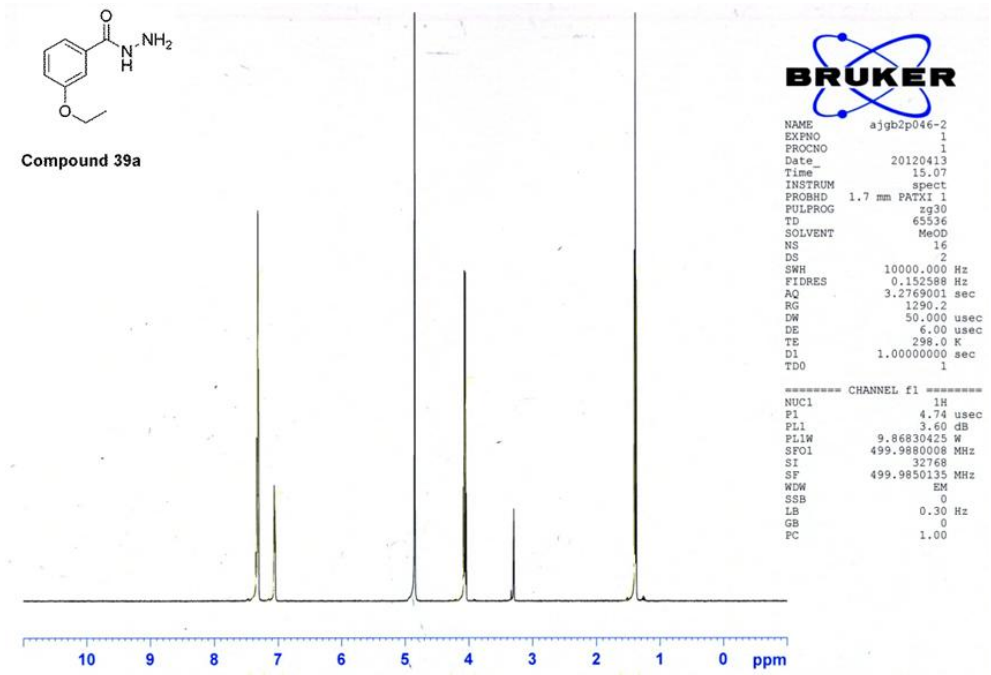

Figure S57. TIC and MS spectrum of compound **39a**.

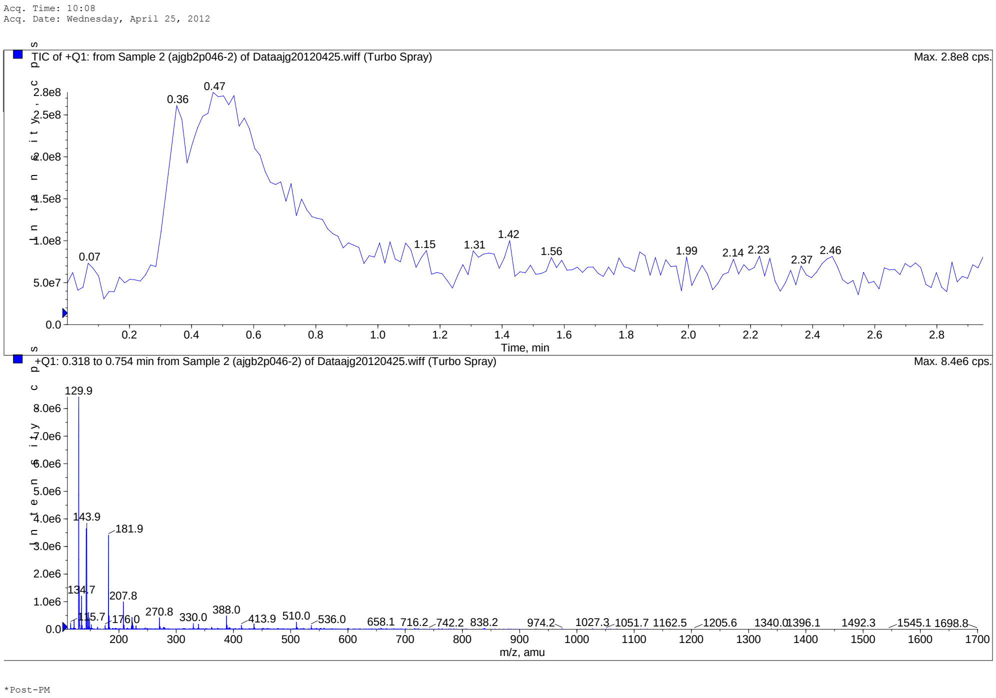

Figure S58. ^{1}H NMR spectrum of compound **39b**.

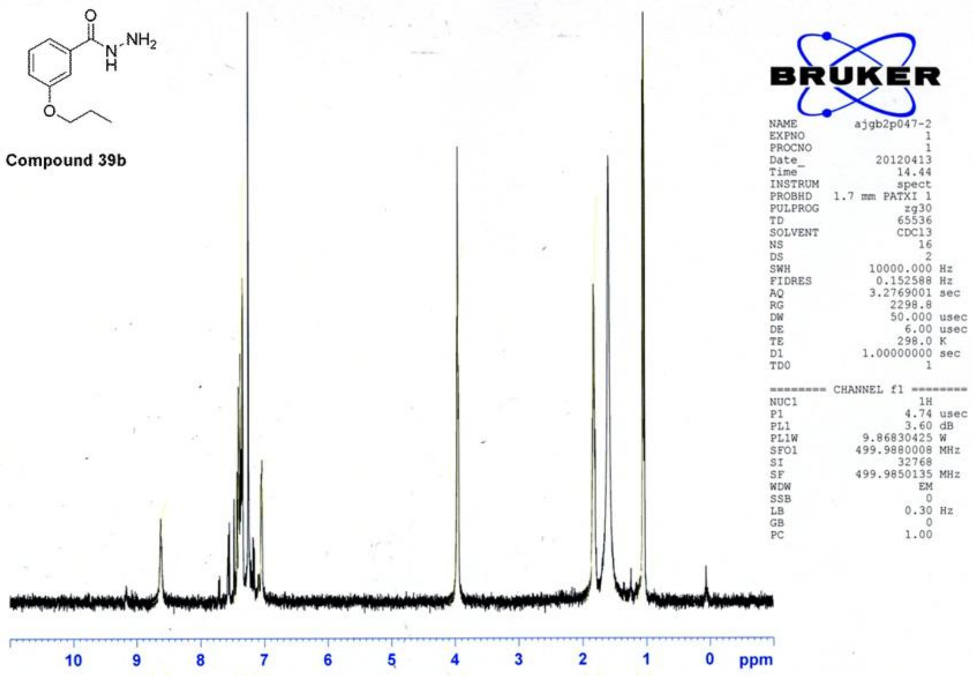

Figure S59. TIC and MS spectrum of compound **39b**.

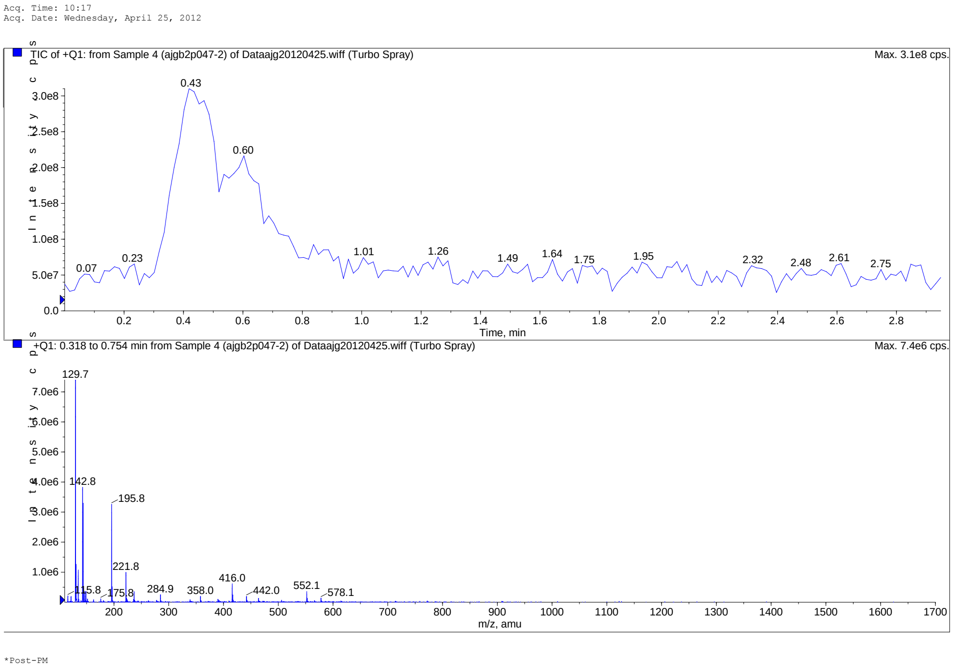

Figure S60. ^{1}H NMR spectrum of compound **39c**.

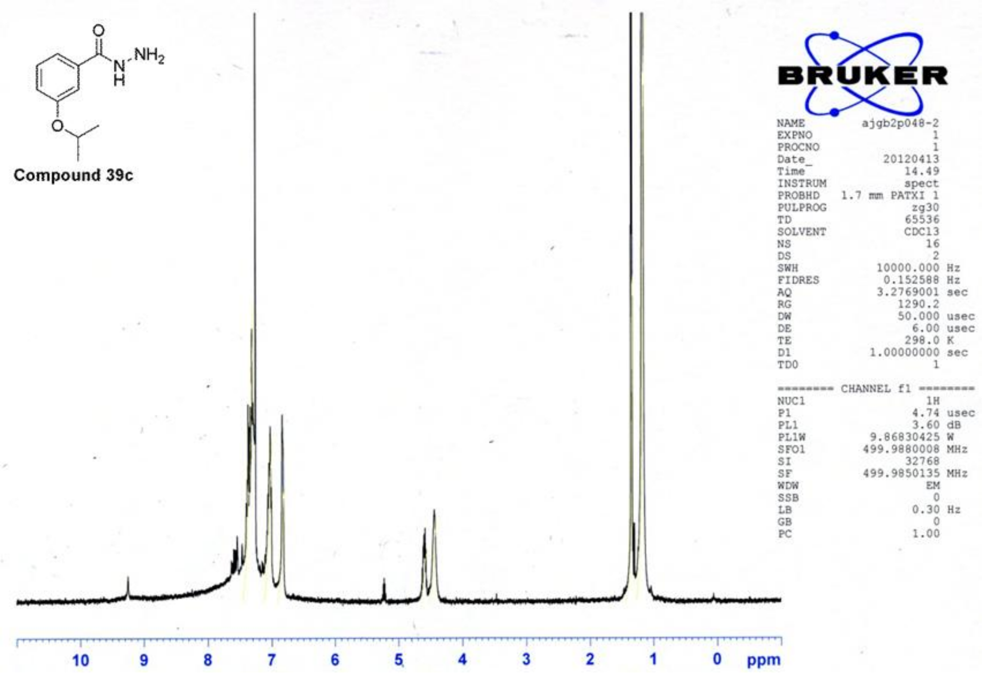

Figure S61. TIC and MS spectrum of compound **39c**.

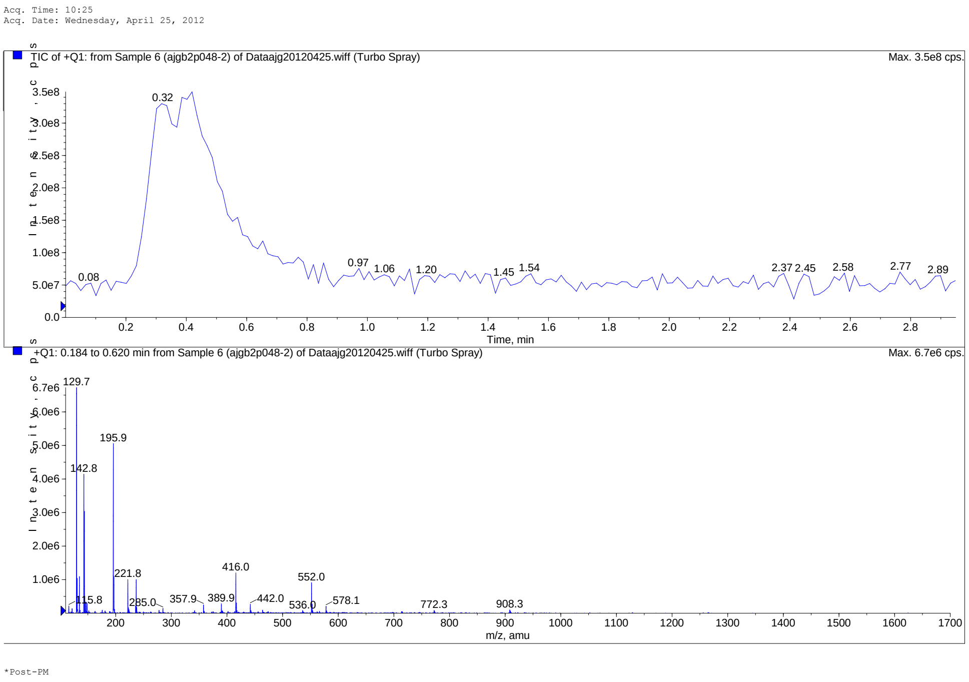

Figure S62. ^{1}H NMR spectrum of compound **39d**.

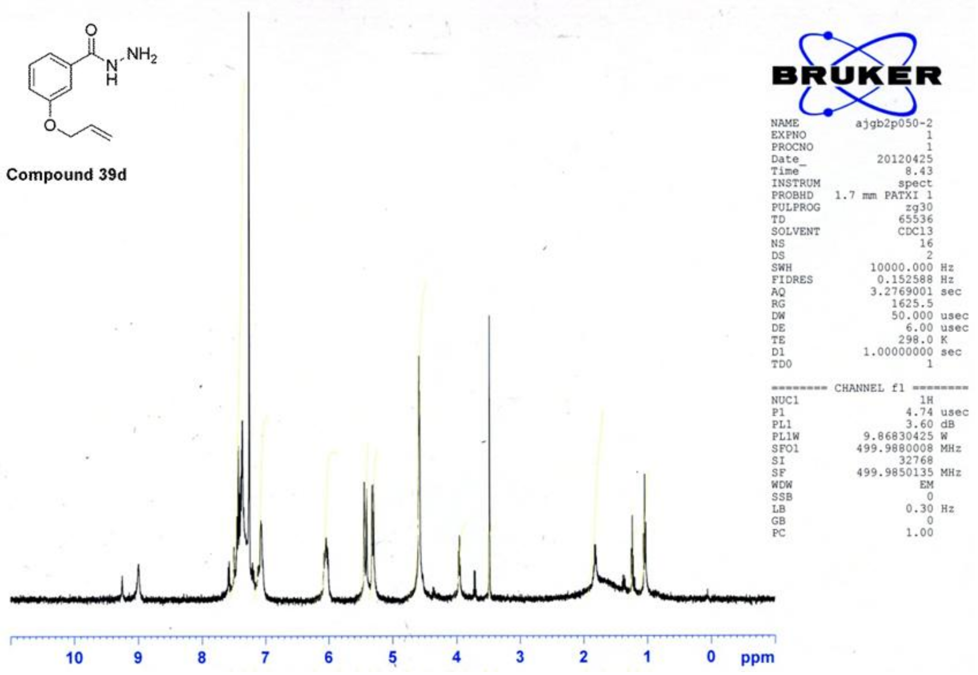

Figure S63. TIC and MS spectrum of compound **39d**.

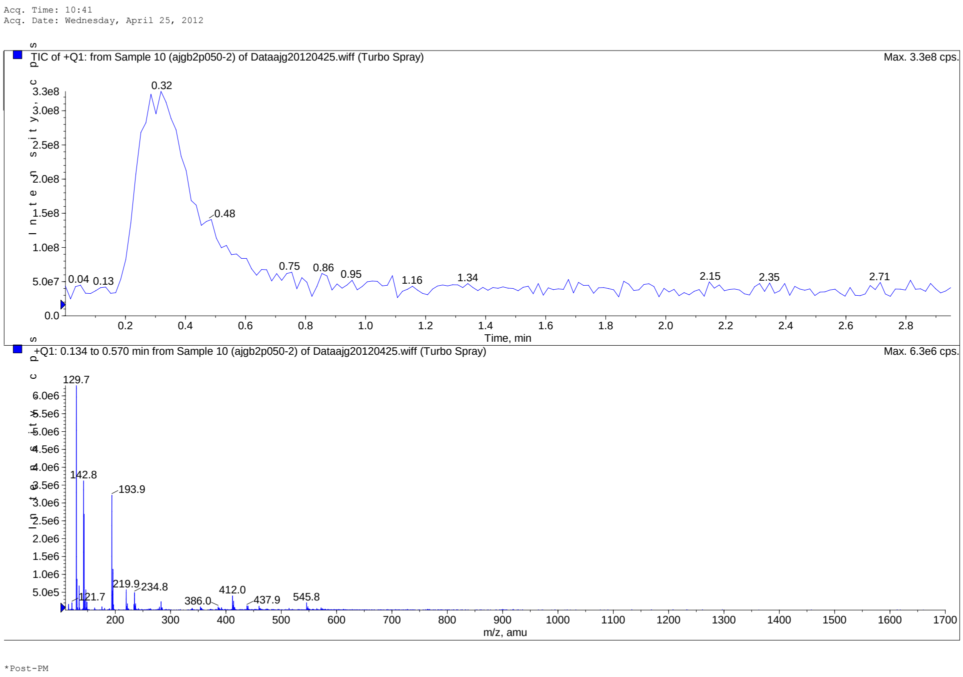

Figure S64. ^{1}H NMR spectrum of compound **41**.

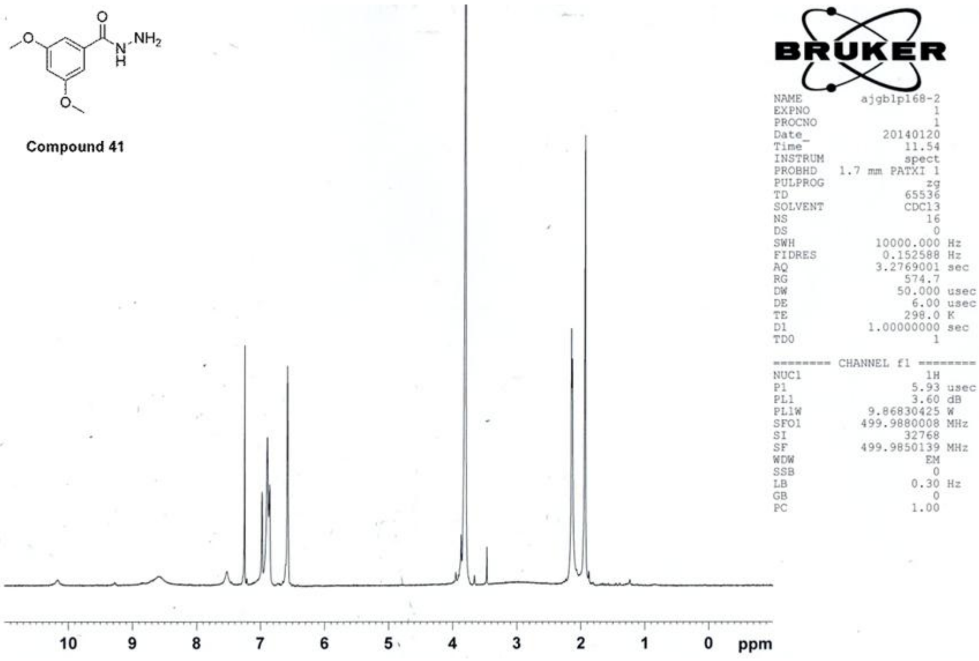

Figure S65. TIC and MS spectrum of compound **41**.

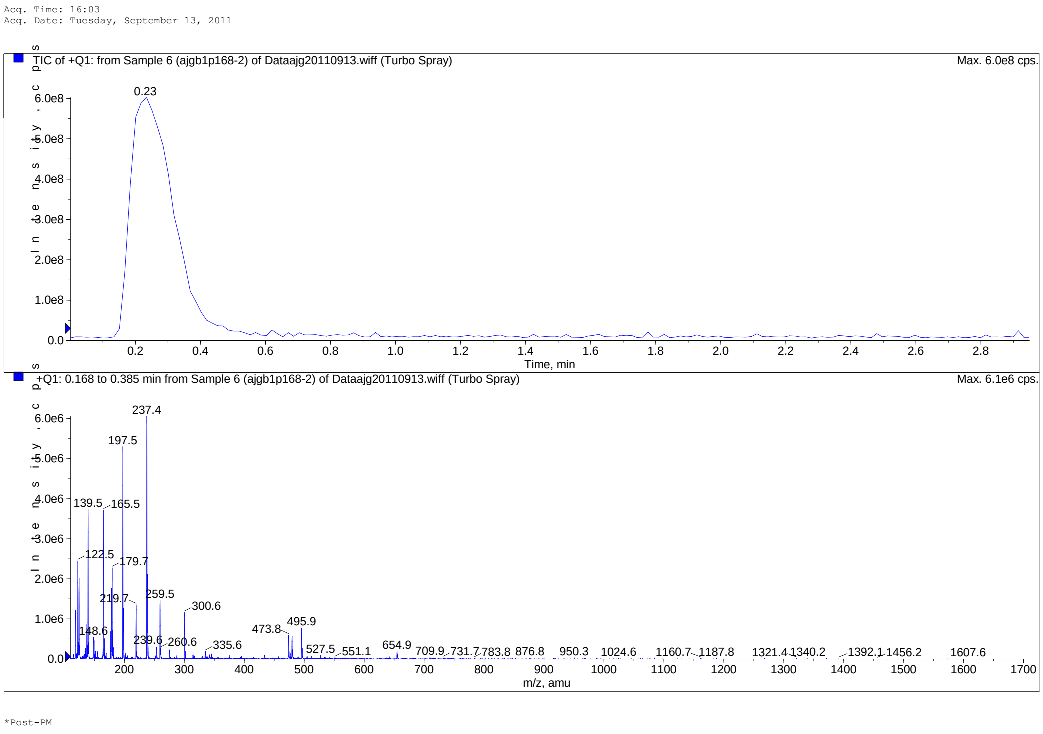

Figure S66. ^{1}H NMR spectrum of compound **44a**.

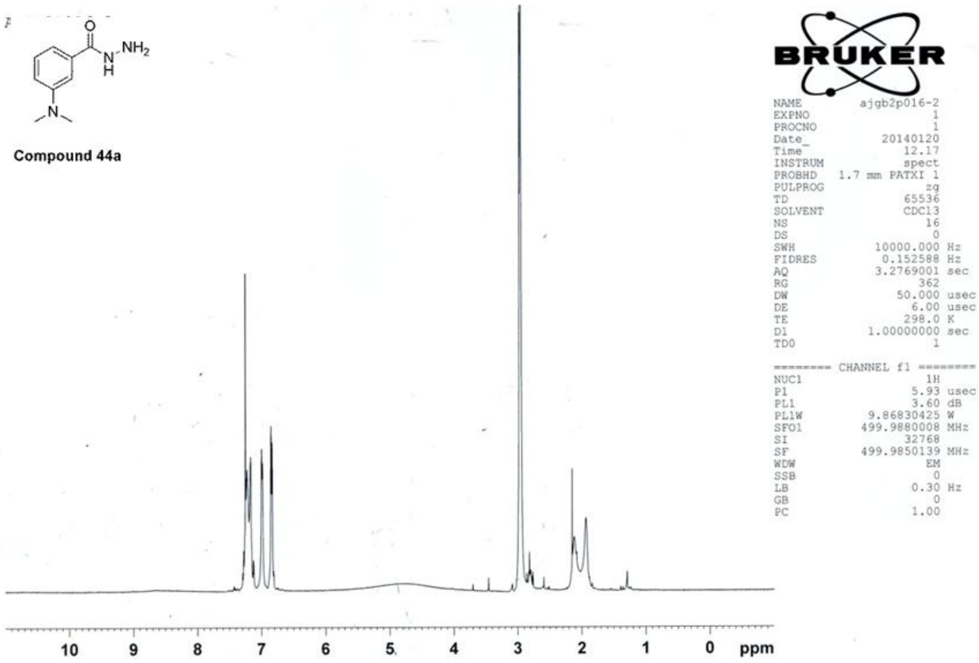

Figure S67. TIC and MS spectrum of compound **44a**.

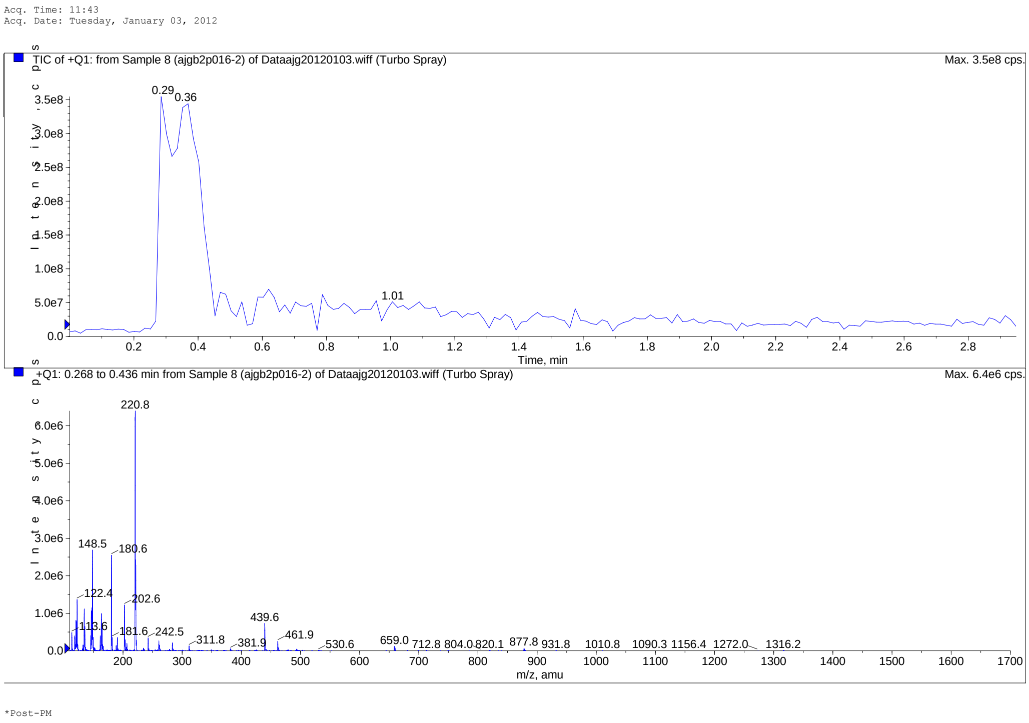

Figure S68. ^{1}H NMR spectrum of compound **44b**.

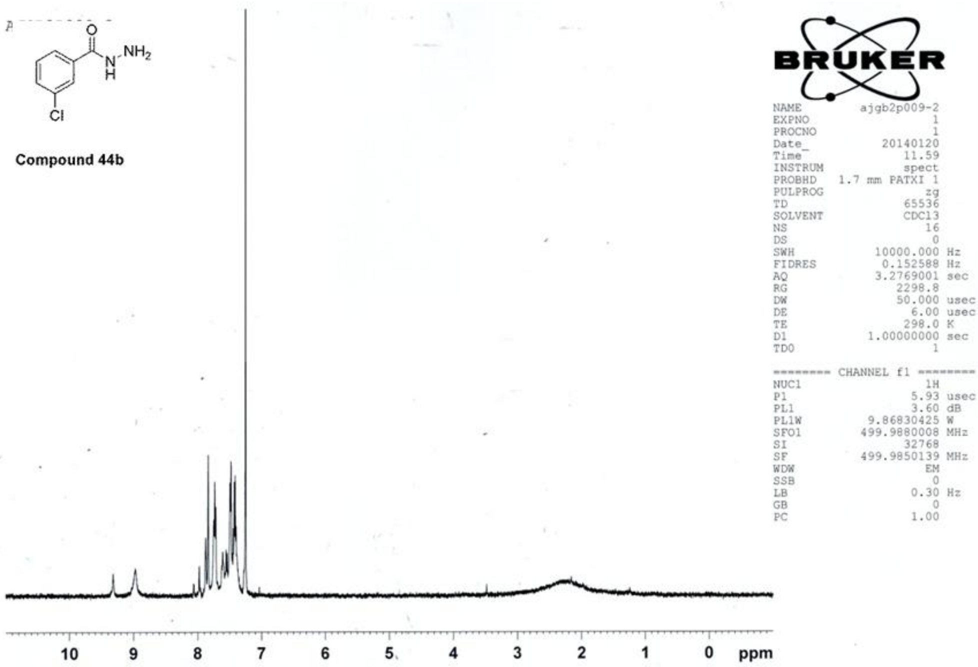

Figure S69. TIC and MS spectrum of compound **44b**.

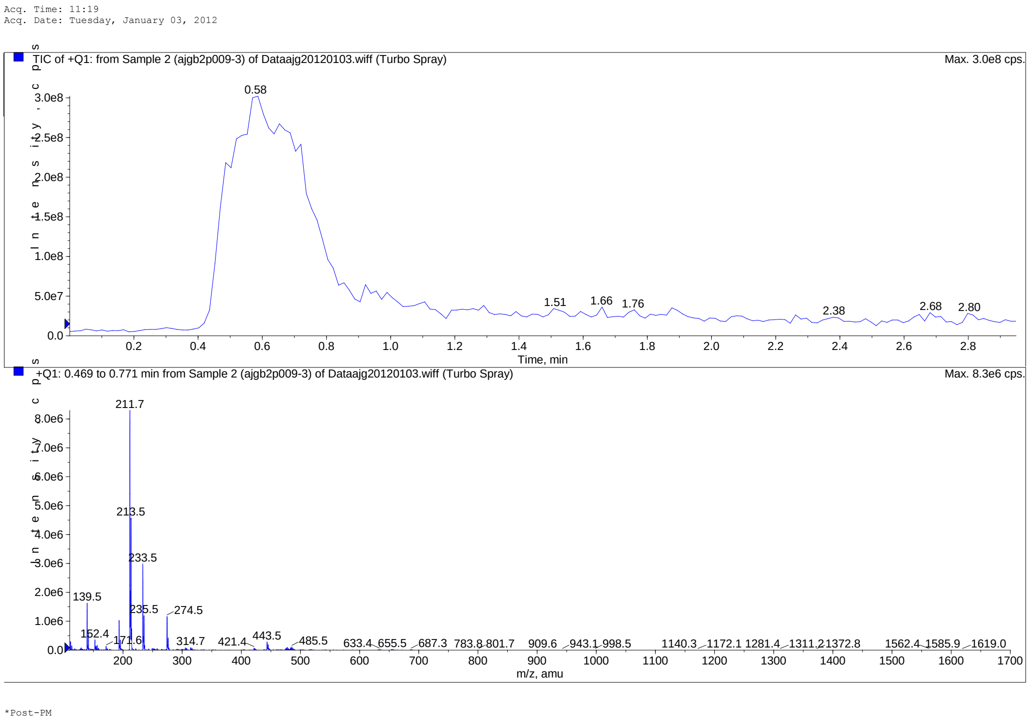

Figure S70. ^{1}H NMR spectrum of compound **44c**.

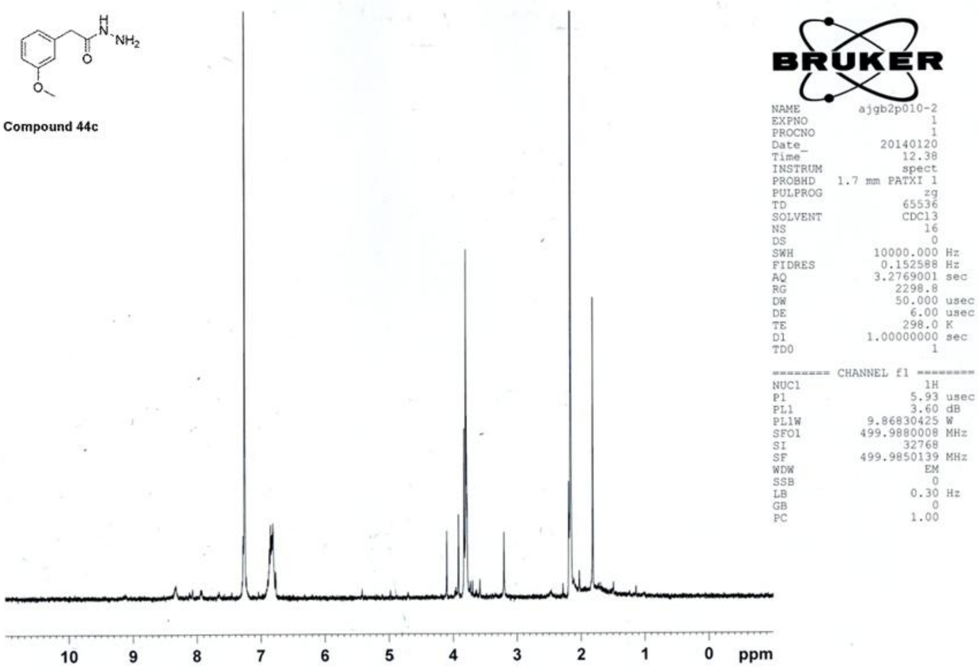

Figure S71. TIC and MS spectrum of compound **44c**.

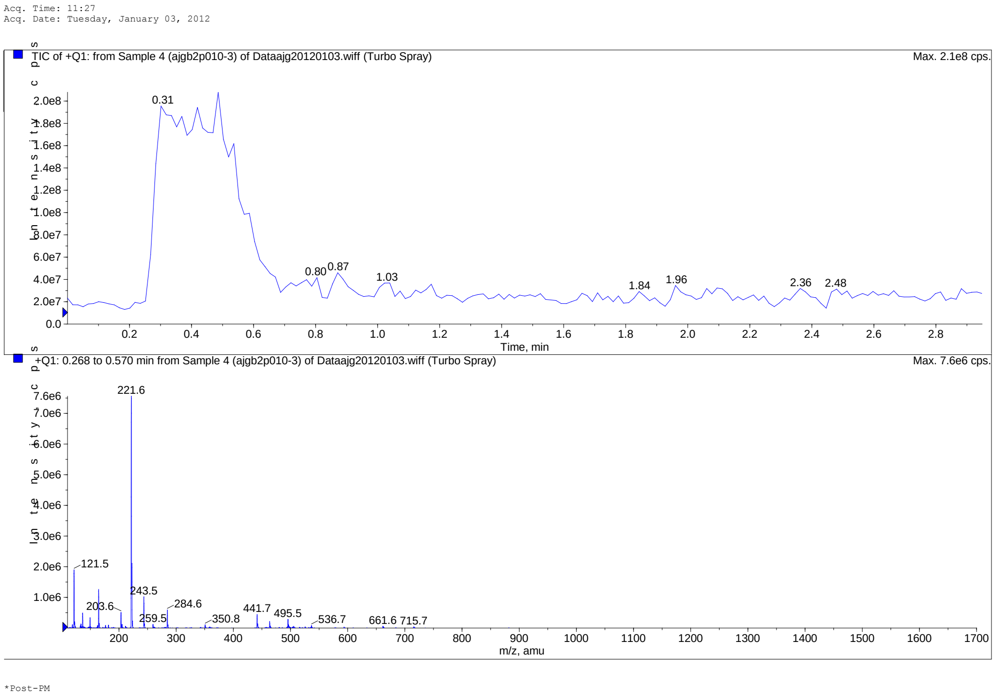

Figure S72. ^{1}H NMR spectrum of compound **44d**.

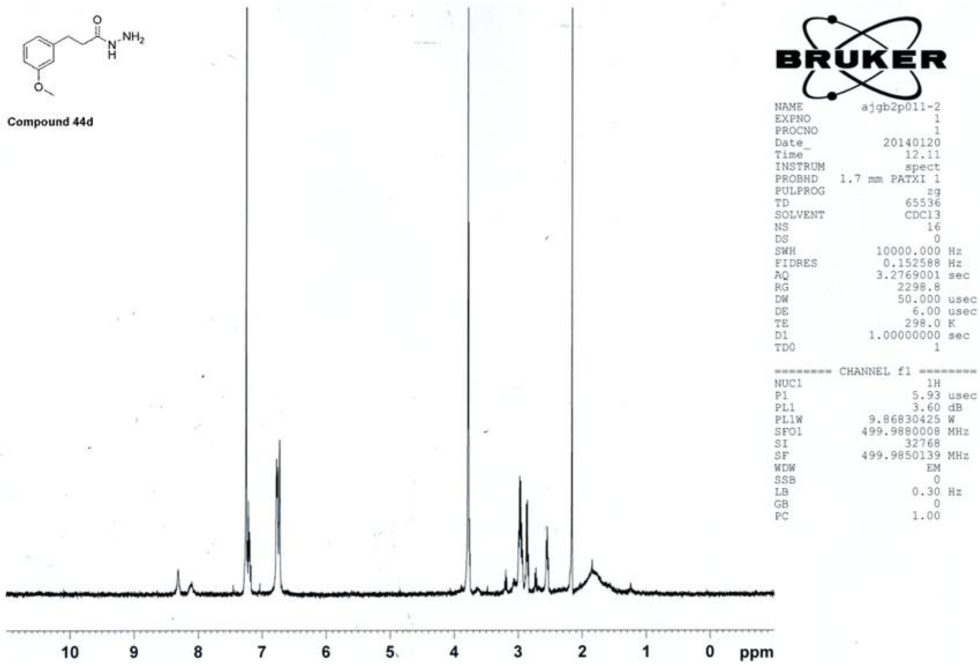

Figure S73. TIC and MS spectrum of compound **44d**.

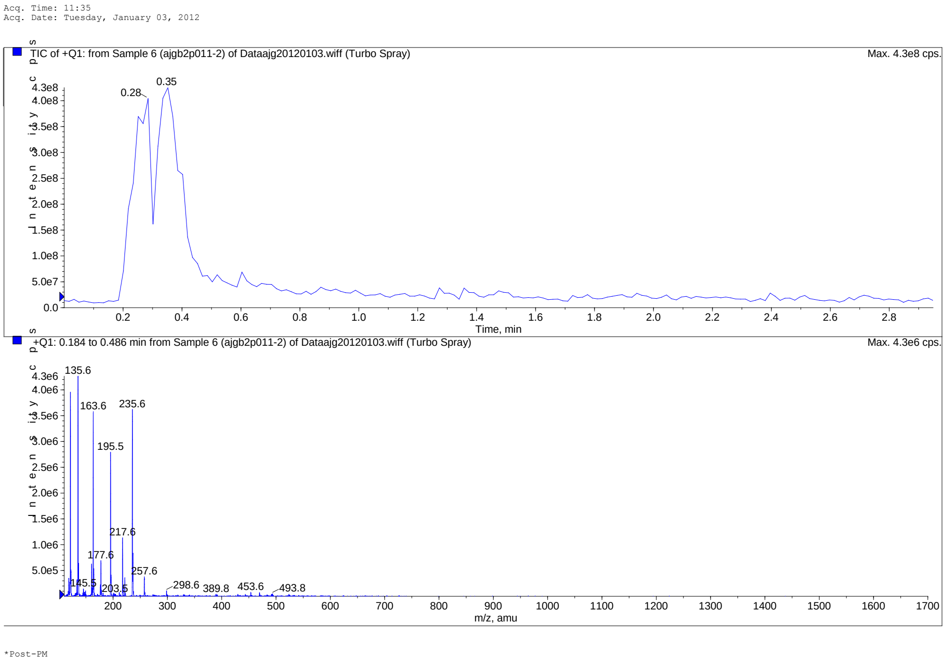

© 2014 by the authors; licensee MDPI, Basel, Switzerland. This article is an open access article distributed under the terms and conditions of the Creative Commons Attribution license (http://creativecommons.org/licenses/by/3.0/).
